# Prevalence, Awareness, Treatment, and Control of Type 2 Diabetes in South Korea (1998 to 2022): Nationwide Cross-Sectional Study

**DOI:** 10.2196/59571

**Published:** 2024-08-27

**Authors:** Wonwoo Jang, Seokjun Kim, Yejun Son, Soeun Kim, Hyeon Jin Kim, Hyesu Jo, Jaeyu Park, Kyeongmin Lee, Hayeon Lee, Mark A Tully, Masoud Rahmati, Lee Smith, Jiseung Kang, Selin Woo, Sunyoung Kim, Jiyoung Hwang, Sang Youl Rhee, Dong Keon Yon

**Affiliations:** 1 Department of Medicine Kyung Hee University College of Medicine Seoul Republic of Korea; 2 Center for Digital Health Medical Science Research Institute Kyung Hee University College of Medicine Seoul Republic of Korea; 3 Department of Precision Medicine Kyung Hee University College of Medicine Seoul Republic of Korea; 4 Department of Regulatory Science Kyung Hee University Seoul Republic of Korea; 5 School of Medicine Ulster University Londonderry United Kingdom; 6 CEReSS-Health Service Research and Quality of Life Center Aix-Marseille University Marseille France; 7 Department of Physical Education and Sport Sciences Faculty of Literature and Human Sciences Lorestan University Khoramabad Iran; 8 Department of Physical Education and Sport Sciences Faculty of Literature and Humanities Vali-E-Asr University of Rafsanjan Rafsanjan Iran; 9 Centre for Health, Performance and Wellbeing Anglia Ruskin University Cambridge United Kingdom; 10 Division of Sleep Medicine Harvard Medical School Boston, MA United States; 11 Department of Anesthesia, Critical Care and Pain Medicine Massachusetts General Hospital Boston, MA United States; 12 Department of Family Medicine Kyung Hee University Medical Center Kyung Hee University College of Medicine Seoul Republic of Korea; 13 Department of Endocrinology and Metabolism Kyung Hee University School of Medicine Seoul Republic of Korea; 14 Department of Pediatrics Kyung Hee University College of Medicine Seoul Republic of Korea

**Keywords:** disease management, epidemiology, prevalence, Republic of Korea, type 2 diabetes mellitus

## Abstract

**Background:**

Type 2 diabetes poses an increasing disease burden in South Korea. The development and management of type 2 diabetes are closely related to lifestyle and socioeconomic factors, which have undergone substantial changes over the past few decades, including during the COVID-19 pandemic.

**Objective:**

This study aimed to investigate long-term trends in type 2 diabetes prevalence, awareness, treatment, and control. It also aimed to determine whether there were substantial alterations in the trends during the pandemic and whether these changes were more pronounced within specific demographic groups.

**Methods:**

This study examined the prevalence, awareness, treatment, and control of type 2 diabetes in a representative sample of 139,786 South Koreans aged >30 years, using data from the National Health and Nutrition Examination Survey and covering the period from 1998 to 2022. Weighted linear regression and binary logistic regression were performed to calculate weighted β coefficients or odds ratios. Stratified analyses were performed based on sex, age, region of residence, obesity status, educational background, household income, and smoking status. β (difference) was calculated to analyze the trend difference between the prepandemic period and the COVID-19 pandemic. To identify groups more susceptible to type 2 diabetes, we estimated interaction terms for each factor and calculated weighted odds ratios.

**Results:**

From 1998 to 2022, a consistent increase in the prevalence of type 2 diabetes was observed among South Koreans, with a notable rise to 15.61% (95% CI 14.83-16.38) during the pandemic. Awareness followed a U-shaped curve, bottoming out at 64.37% (95% CI 61.79-66.96) from 2013 to 2015 before increasing to 72.56% (95% CI 70.39-74.72) during the pandemic. Treatment also increased over time, peaking at 68.33% (95% CI 65.95-70.71) during the pandemic. Control among participants with diabetes showed no substantial change, maintaining a rate of 29.14% (95% CI 26.82-31.47) from 2020 to 2022, while control among treated participants improved to 30.68% (95% CI 27.88-33.48). During the pandemic, there was a steepening of the curves for awareness and treatment. However, while the slope of control among participants being treated increased, the slope of control among participants with diabetes showed no substantial change during the pandemic. Older populations and individuals with lower educational level exhibited less improvement in awareness and control trends than younger populations and more educated individuals. People with lower income experienced a deceleration in prevalence during the pandemic.

**Conclusions:**

Over the recent decade, there has been an increase in type 2 diabetes prevalence, awareness, treatment, and control. During the pandemic, a steeper increase in awareness, treatment, and control among participants being treated was observed. However, there were heterogeneous changes across different population groups, underscoring the need for targeted interventions to address disparities and improve diabetes management for susceptible populations.

## Introduction

### Background

Noncommunicable diseases (NCDs) are conditions caused by external factors or lifestyle habits, rather than infections or contagions, and are difficult to manage and cure once diagnosed [1-3]. According to the World Health Organization, NCDs are responsible for around 41 million deaths annually, representing approximately 74% of all global deaths. Type 2 diabetes is one of the leading NCDs, affecting an estimated 529 million people globally in 2021, representing an increase in age-standardized prevalence from 3.2% in 1990 to 6.1% in 2021 [4]. In South Korea, the prevalence of type 2 diabetes among adults aged ≥30 years was estimated to be 16.7% in 2020, and the prevalence of type 2 diabetes increased from 7.9% in 2009 to 12.4% in 2021 [5]. This prevalence has made type 2 diabetes a chronic disease that needs to be managed at the national level. Although type 2 diabetes is known to be strongly influenced by genetics, it is also closely associated with obesity and other behavioral factors [6].

The COVID-19 pandemic is also an important factor that must be considered in the analysis of recent trends in diabetes. The direct and indirect effects of COVID-19 on diabetes have been consistently emphasized across various studies [7-10]. Therefore, understanding the trends of the prevalence, awareness, treatment, and control rate of diabetes, including during the COVID-19 pandemic, is essential to develop effective prevention and management strategies for type 2 diabetes.

### Prior Work

Previous studies on type 2 diabetes in South Korea have focused primarily on trends in prevalence or differences between sociodemographic groups [11,12]. While these studies have provided important data on type 2 diabetes, they have been limited in addressing complex aspects of diabetes, such as awareness, treatment, and control, or detailed analyses by specific factors, such as socioeconomic factors and lifestyle factors. In addition, studies exploring the impact of a public health crisis, such as the COVID-19 pandemic, on type 2 diabetes management have not been fully extended. Building on the foundation of previous studies, our study aimed to examine the prevalence of type 2 diabetes more comprehensively as well as trends in its awareness, treatment, and control among South Koreans aged ≥30 years from 1998 to 2022. Our study would provide a profound understanding of the epidemiology of type 2 diabetes during the pandemic and contribute to developing tailored public health interventions for more effective prevention and management of type 2 diabetes in high-risk populations.

## Methods

### Survey Design and Participants

We conducted a comprehensive analysis that included a range of socioeconomic variables to examine trends in type 2 diabetes and identify susceptible groups. The data used in this study were from the Korea National Health and Nutrition Examination Survey (KNHANES) conducted by the Korea Disease Control and Prevention Agency (KDCA) from 1998 to 2022 [13]. Data from the KNHANES consisted of a randomly selected, representative sample of the South Korean population using the latest population and housing census data. The selected sample included detailed information on age, sex, area of residence, BMI, educational background, smoking status, and household income. The study population was specifically selected to be individuals aged ≥30 years [14]. This age criterion was chosen based on the markedly low prevalence of type 2 diabetes in individuals aged <30 years, which poses a challenge in conducting statistically significant analyses within this younger population. A total of 139,786 representative participants were selected for the 25-year study period, which was divided into the following subperiods: 44.42% (62,090/139,786) in 1998 to 2005; 10.4% (14,537/139,786) in 2007 to 2009; 11.12% (15,549/139,786) in 2010 to 2012; 9.57% (13,382/139,786) in 2013 to 2015; 14.68% (20,519/139,786) in 2016 to 2019; and 9.81% (13,709/139,786) in 2020 to 2022.

The categorization by year follows the sampling design principles of the KNHANES, with particular attention given to the period between 2020 and 2022 to examine the association of the COVID-19 pandemic in depth.

### Ethical Considerations

The research protocol was approved by the institutional review boards of the KDCA (2007-02CON-04-P, 2008-04EXP-01-C, 2009-01CON-03-2C, 2010-02CON-21-C, 2011-02CON-06-C, 2012-01EXP-01-2C, 2013-07CON-03-4C, and 2013-12EXP-035C). This approval also adhered to the requirements outlined in the Bioethics and Safety Act, specifically article 2, paragraph 1 of the Act and article 2, paragraph 2, item 1 of its Enforcement Regulation, as mandated by Korean law. The secondary analysis in this study was approved by the institutional review board of Kyung Hee University. We obtained written informed consent from all participants before their participation and used anonymized data in this study as there is no need to provide compensation or medical findings reports to participants. The study was conducted in accordance with the principles outlined in the Declaration of Helsinki and pursuant to Article 20 of the National Health Promotion Act.

### Health Outcomes

The independent variables in this study were prevalence, awareness, treatment, control among participants with diabetes, and control among participants being treated. In individuals who are not pregnant, type 2 diabetes was defined as having a fasting blood glucose level of ≥126 mg/dL, receiving a diagnosis from a physician, taking type 2 diabetes medication or insulin injections for blood glucose management, or having a glycated hemoglobin level of ≥6.5%. Awareness was defined as the percentage of participants with type 2 diabetes who responded “yes” to the question, “Have you ever been told by a doctor or other health care professional that you have diabetes?” [15] Treatment was defined as the percentage of people with type 2 diabetes taking diabetes medication or receiving insulin injections. Control among participants with diabetes was defined as the percentage of people with type 2 diabetes with a glycosylated hemoglobin level <6.5%. Control among participants being treated was similarly defined as the proportion of individuals taking diabetes medication or receiving insulin injections who had a glycated hemoglobin level <6.5%.

### Covariates

The covariates included in our analysis were sex, age (30-39, 40-49, 50-59, 60-69, and ≥70 years), region of residence (urban and rural) [16-18], BMI group according to Asian-Pacific guidelines (underweight [<18.5 kg/m^2^], normal [18.5-22.9 kg/m^2^], overweight [23.0-24.9 kg/m^2^], obese [≥25.0 kg/m^2^], and unknown) [19,20], educational background (elementary school or lower, middle school, high school, and college or higher), household income (lowest, second, third, and highest quartiles), smoking status (current, ex-smoker, nonsmoker, and unknown), and type 2 diabetes status (normal, impaired fasting glucose, type 2 diabetes, and unknown). Participants’ region of residence was categorized based on their survey responses, and household income was divided using quartiles of standardized income based on sample household and population data from the KNHANES. These covariates were considered associated factors of type 2 diabetes.

### Statistical Analysis

We conducted a weighted complex sampling analysis to estimate the national prevalence, awareness, treatment, and control rates of type 2 diabetes. The KNHANES uses a default set weight that considers factors such as survey year and region of residence. This weight is initially derived from 2 primary weights, accounting for the sampling rate and the response rate. Subsequently, this weight undergoes detailed calibration against the sum of weights and the population. This calibration enhances the accuracy and representativeness of the estimates concerning the health behaviors and the prevalence of chronic diseases within the population. In this study, 2 types of sample weights were used. The first weight, derived by multiplying the default weight by the proportion corresponding to each survey period annually over the 15-year period, was used to ascertain overall estimates across the entire 15 years. The second weight, derived by multiplying the default weight by the proportion corresponding to the number of households for the study subperiod, was used to adjust observations for each subperiod. Linear regression and binary logistic regression models were used to calculate weighted β coefficients with 95% CIs or weighted odds ratios (wORs) with 95% CIs [21,22]. Stratified analyses were performed in all regression models to ensure accurate estimation, considering sex, age, region of residence, BMI, educational background, household income, and smoking status. During this process, particular attention was given to the period of the COVID-19 pandemic. We calculated prepandemic β as the slope from the 1998-2005 to 2016-2019 subperiods and pandemic β as the slope from the 2016-2019 to 2020-2022 subperiods. The difference between the latter and the former was defined as “β (difference).” In addition, to identify groups more susceptible to type 2 diabetes, we estimated interaction terms for each associated factor and calculated wORs. Furthermore, to examine sex differences in type 2 diabetes, all analyses were divided by sex for additional investigation. For statistical analyses, our study used the SAS software (version 9.4; SAS Institute), with a 2-sided test, and a *P* value <.05 was considered statistically significant [22].

## Results

**Figure 1:**
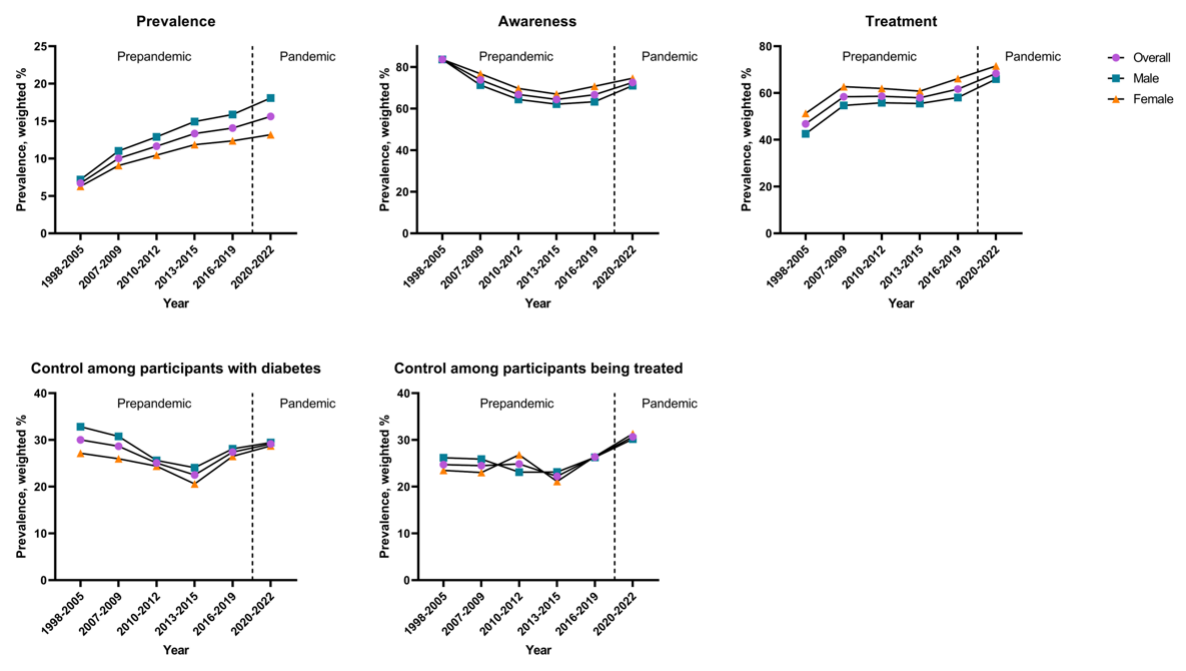
Serial cross-sectional type 2 diabetes prevalence, awareness, treatment, control among participants with diabetes, and control among participants being treated in a population aged >30 years in South Korea during the periods from 1998 to 2005 (62,090/139,786, 44.42%), from 2007 to 2009 (14,537/139,786, 10.4%), from 2010 to 2012 (15,549/139,786, 11.12%), from 2013 to 2015 (13,382/139,786, 9.57%), from 2016 to 2019 (20,519/139,786, 14.68%), and from 2020 to 2022 (13,709/139,786, 9.81%).

The characteristics of the study population are detailed in Table 1. Figure 1 illustrates the trends in the prevalence, awareness, treatment, and control of type 2 diabetes in the South Korean population from 1998 to 2022.

**Table 1:** Weighted characteristics of the South Korean population included in the serial cross-sectional analysis of type 2 diabetes during the periods from 1998 to 2005, from 2007 to 2009, from 2010 to 2012, from 2013 to 2015, from 2016 to 2019, and from 2020 to 2022a.

Characteristics		Total (N=139,786)	1998-2005 (n=62,090)	2007-2009 (n=14,537)	2010-2012 (n=15,549)	2013-2015 (n=13,382)	2016-2019 (n=20,519)	2020-2022 (n=13,709)
**Sex, weighted % (95% CI)**								
	Male	48.94 (48.63-49.26)	48.38 (48.09-48.68)	49.25 (48.52-49.98)	48.85 (48.13-49.57)	48.11 (47.33-48.89)	49.01 (48.37-49.64)	49.49 (48.72-50.26)
	Female	51.06 (50.74-51.37)	51.62 (51.32-51.91)	50.75 (50.02-51.48)	51.15 (50.43-51.87)	51.89 (51.11-52.67)	50.99 (50.36-51.63)	50.51 (49.74-51.28)
**Age group (y), weighted % (95% CI)**								
	30-39	23.29 (22.73-23.85)	30.95 (30.11-31.80)	27.81 (26.34-29.27)	25.62 (24.33-26.92)	23.23 (21.91-24.55)	21.35 (20.24-22.46)	19.81 (18.53-21.08)
	40-49	25.54 (25.06-26.03)	28.96 (28.27-29.64)	28.33 (27.12-29.54)	27.03 (25.81-28.24)	25.63 (24.51-26.74)	24.59 (23.64-25.53)	23.07 (21.88-24.26)
	50-59	23.24 (22.79-23.69)	17.65 (17.13-18.17)	20.55 (19.59-21.52)	22.64 (21.60-23.68)	24.23 (23.16-25.30)	24.32 (23.45-25.19)	24.02 (22.89-25.14)
	60-69	15.35 (15.00-15.70)	13.3 (12.85-13.74)	12.87 (12.20-13.54)	13.19 (12.50-13.88)	14.37 (13.62-15.13)	16.15 (15.40-16.90)	19.03 (18.09-19.97)
	≥70	12.57 (12.23-12.92)	9.15 (8.75-9.54)	10.44 (9.74-11.14)	11.52 (10.80-12.24)	12.54 (11.75-13.32)	13.6 (12.84-14.35)	14.08 (13.13-15.03)
**Region of residence, weighted % (95% CI)**								
	Urban	81.54 (80.23-82.85)	79.52 (78.72-80.32)	79.03 (75.80-82.25)	78.35 (74.90-81.79)	81.46 (78.44-84.49)	83.8 (81.25-86.36)	83.48 (80.46-86.50)
	Rural	18.46 (17.15-19.77)	20.48 (19.68-21.28)	20.97 (17.75-24.20)	21.65 (18.21-25.10)	18.54 (15.51-21.56)	16.2 (13.64-18.75)	16.52 (13.50-19.54)
**BMI group, weighted % (95% CI)^b^**								
	Underweight	3.15 (3.00-3.30)	0.82 (0.70-0.93)	3.38 (3.04-3.72)	3.19 (2.83-3.55)	3.19 (2.85-3.53)	3.11 (2.82-3.39)	3.2 (2.81-3.59)
	Normal	35.84 (35.42-36.25)	8.97 (8.14-9.79)	37.03 (36.09-37.98)	37.51 (36.50-38.52)	37.87 (36.90-38.84)	36.45 (35.63-37.27)	33.86 (32.87-34.86)
	Overweight	23.6 (23.24-23.96)	6.13 (5.55-6.71)	25.01 (24.20-25.83)	24.26 (23.40-25.12)	24.45 (23.60-25.30)	23.82 (23.12-24.53)	22.86 (22.00-23.73)
	Obese	35.18 (34.75-35.61)	8.03 (7.28-8.78)	34 (33.04-34.95)	34.58 (33.56-35.59)	34.4 (33.47-35.33)	36.27 (35.43-37.11)	38.69 (37.60-39.78)
	Unknown	2.23 (2.14-2.33)	76.05 (73.95-78.16)	0.58 (0.41-0.75)	0.46 (0.30-0.61)	0.09 (0.02-0.15)	0.35 (0.25-0.46)	1.39 (1.16-1.62)
**Educational background, weighted % (95% CI)**								
	Elementary school or lower	17.39 (16.95-17.84)	25.18 (24.39-25.98)	23.04 (21.87-24.20)	21.02 (19.90-22.14)	18.2 (17.11- 19.30)	14.96 (14.07-15.84)	11.87 (10.92-12.82)
	Middle school	11.02 (10.72-11.31)	13.76 (13.30-14.23)	13.01 (12.27-13.75)	12.46 (11.74-13.18)	11.29 (10.60-11.98)	10.23 (9.64-10.82)	8.84 (8.18-9.51)
	High school	31.83 (31.33-32.33)	36.8 (36.06-37.54)	33.83 (32.60-35.06)	33.12 (31.93-34.32)	32.17 (30.99-33.35)	30.18 (29.20-31.16)	30.58 (29.42-31.74)
	College or higher	39.76 (39.03-40.49)	24.26 (23.33-25.18)	30.13 (28.55-31.71)	33.39 (31.86-34.93)	38.34 (36.76-39.91)	44.64 (43.07-46.21)	48.71 (46.91-50.51)
**Household income, weighted % (95% CI)**								
	Lowest quartile	16.49 (16.01-16.97)	22.72 (21.82-23.62)	17.29 (16.11-18.47)	17.55 (16.42-18.67)	16.61 (15.44-17.79)	16.18 (15.18-17.17)	14.68 (13.58-15.77)
	Second quartile	24.7 (24.17-25.22)	24.91 (24.15-25.67)	24.76 (23.50-26.01)	27.54 (26.21-28.87)	24.49 (23.25-25.74)	24.27 (23.26-25.29)	22.86 (21.64-24.07)
	Third quartile	28.77 (28.24-29.30)	26.03 (25.31-26.75)	28.51 (27.25-29.78)	28.07 (26.90-29.25)	29.09 (27.72-30.46)	28.72 (27.70-29.73)	29.6 (28.32-30.89)
	Highest quartile	30.05 (29.31-30.78)	26.34 (25.31-27.38)	29.44 (27.59-31.30)	26.84 (25.40-28.28)	29.8 (28.04-31.56)	30.83 (29.33-32.33)	32.86 (30.97-34.76)
**Smoking status, weighted % (95% CI)**								
	Current smoker	21.69 (21.29-22.08)	8.98 (8.16-9.80)	25.72 (24.89-26.56)	25.8 (24.83-26.77)	22.42 (21.48-23.35)	20.42 (19.64-21.20)	17.57 (16.67-18.48)
	Ex-smoker	22.84 (22.50-23.18)	4.52 (4.04-5.00)	21.48 (20.74-22.23)	21.32 (20.53-22.11)	21.3 (20.51-22.09)	24.04 (23.39-24.70)	26.81 (25.96-27.67)
	Nonsmoker	53.95 (53.57- 54.34)	17.33 (15.81-18.85)	52.74 (51.89-53.59)	52.87 (52.00-53.74)	56.28 (55.42-57.14)	55.54 (54.77-56.31)	55.61 (54.60-56.63)
	Unknown	1.52 (1.45-1.59)	69.17 (66.49-71.85)	0.05 (0.00-0.11)	—^c^	—	—	—
**Diabetes, weighted % (95% CI)**								
	Normal	62.04 (61.49-62.59)	86.51 (85.58-87.44)	84.67 (83.88-85.45)	65.39 (63.74-67.04)	53.58 (52.22-54.94)	57.39 (56.36-58.42)	53.62 (52.40-54.84)
	IFG^d^	24.9 (24.43-25.36)	6.78 (5.98-7.58)	5.32 (4.87-5.76)	22.97 (21.49-24.46)	33.09 (31.90-34.27)	28.53 (27.70-29.36)	30.77 (29.76-31.79)
	Type 2 diabetes	13.07 (12.77-13.36)	6.71 (6.42-7.00)	10.02 (9.40-10.63)	11.64 (10.99-12.29)	13.33 (12.61-14.06)	14.07 (13.45-14.70)	15.61 (14.83-16.38)

^a^Data were obtained from the Korea National Health and Nutrition Examination Survey (KNHANES).

^b^BMI was divided into 4 groups according to Asian-Pacific guidelines: underweight (<18.5 kg/m^2^), normal (18.5-22.9 kg/m^2^), overweight (23.0-24.9 kg/m^2^), and obese (≥25 kg/m^2^).

^c^Not applicable.

^d^IFG: impaired fasting glucose.

Table S1 in Multimedia Appendix 1 shows the results of trend analysis concerning each socioeconomic subclassification. Since 1998, the prevalence of type 2 diabetes consistently increased, and it reached 15.61% (95% CI 14.83-16.38) during the pandemic. Awareness exhibits a U-shaped curve, having a nadir of 64.37% (95% CI 61.79-66.96) from 2013 to 2015. Awareness experienced a notable upsurge during the pandemic and stands at 72.56% (95% CI 70.39-74.72). The treatment curve tended to rise over time and reached 68.33% (95% CI 65.95-70.71), marking a notable increase during the pandemic. The control among participants with diabetes was 30.00% (95% CI 26.58-33.42) between 1998 and 2005 and 29.14% (95% CI 26.82-31.47) between 2020 and 2022, suggesting no notable trend change over the study period. From 1998 to 2019, the control among participants being treated showed a marginal increase, consistently hovering around 25%. However, during the pandemic, the control among participants being treated surged to 30.68% (95% CI 27.88-33.48), accompanied by an increased slope (β [difference]=3.92, 95% CI 0.30-7.55).

Table 2 presents the wOR associated with each statistic across socioeconomic factors. Compared to male participants, female participants showed lower prevalence (wOR 0.75, 95% CI 0.72-0.79), higher awareness (wOR 1.28, 95% CI 1.16-1.41), higher treatment (wOR 1.31, 95% CI 1.20-1.44), and lower control participants with diabetes (wOR 0.90, 95% CI 0.81-1.00). Advanced age was associated with relatively high prevalence, awareness, treatment, control among participants with diabetes, and control among participants being treated. People residing in rural regions exhibited higher prevalence than people residing in urban regions (wOR 1.28, 95% CI 1.19-1.37). The population with higher BMI possessed relatively high prevalence. People with obesity showed lower awareness (wOR 0.52, 95% CI 0.31-0.86), control among participants with diabetes (wOR 0.61, 95% CI 0.37-1.00), and control among participants being treated (wOR 0.52, 95% CI, 0.27-0.98) than people in the underweight group. The prevalence diminished with ascending educational background. Conversely, educational background was generally inversely correlated with awareness and treatment. Similarly, the prevalence decreased with increasing household income, and the highest quartile had a wOR of 0.35 (95% CI 0.33-0.38) compared to the lowest quartile. By contrast, higher household income tended to be associated with lower awareness, treatment, and control. People with smoking behavior had higher prevalence (wOR 1.28, 95% CI 1.22-1.34), lower awareness (wOR 0.85, 95% CI 0.78-0.94), and treatment (wOR 0.81, 95% CI 0.74-0.88) than nonsmokers.

From the β (difference) in Table S1 in Multimedia Appendix 1, it can be inferred that the slope of the prevalence trend did not change during the pandemic. A notable rise in the β of prevalence was found only among people with a college education or higher (β [difference]=1.59, 95% CI 0.43-2.76). Conversely, individuals in the lower income group showed a disproportionate decrease in the β of prevalence (β [difference]=–3.22, 95% CI –5.65 to –0.80). During the pandemic, there was an upsurge of β associated with awareness, treatment, and control among participants being treated. Particularly, older populations and people with lower educational backgrounds experienced less increase in the β of control and awareness, compared to younger and more educated groups. A more detailed breakdown of Table S1 in Multimedia Appendix 1 based on sex is available in Tables S1 to S6 in Multimedia Appendix 2. Tables S7 to S12 in Multimedia Appendix 2 display the ratio of odds ratios during the pandemic compared to the prepandemic period in each subgroup.

Change in wORs implies that the relative susceptibility of the subgroup has been modified throughout the pandemic. Table S9 in Multimedia Appendix 2 shows a statistically significantly low ratio of odds ratios in awareness and treatment for male participants with overweight or obesity (all *P*<.05). The wORs of awareness and treatment for male smokers, compared to nonsmokers, increased during the pandemic, while the wORs of awareness and treatment for female smokers, compared to nonsmokers, decreased during the pandemic (Table S12 in Multimedia Appendix 2).

**Table 2:** Weighted odds ratios (wORs) of type 2 diabetes prevalence, awareness, treatment, control among participants with diabetes, and control among participants being treated according to various socioeconomic factors in the South Korean population from 1998 to 2022 (N=139,786).

Socioeconomic factors			Prevalence				Awareness				Treatment				Control among participants with diabetes				Control among participants being treated		
			wORs (95% CI)		*P* value		wORs (95% CI)		*P* value		wORs (95% CI)		*P* value		wORs (95% CI)		*P* value	wORs (95% CI)			*P* value
**Sex**																					
	Male	1.00 (reference)		—^a^		1.00 (reference)		—		1.00 (reference)		—		1.00 (reference)		—		1.00 (reference)		—	
	Female	*0.75 (0.72-0.79)* ^b^		*<.001*		*1.28 (1.16-1.41)*		*<.001*		*1.31 (1.20-1.44)*		*<.001*		*0.90 (0.81-1.00)*		*.04*		1.01 (0.89-1.14)		.88	
**Age (years)**																					
	30-39	1.00 (reference)		—		1.00 (reference)		—		1.00 (reference)		—		1.00 (reference)		—		1.00 (reference)		—	
	40-49	*2.80 (2.45-3.21)*		*<.001*		*1.43 (1.10-1.85)*		*.008*		*1.55 (1.18-2.04)*		*.002*		0.88 (0.65-1.18)		.39		1.01 (0.57-1.79)		.97	
	50-59	*5.94 (5.25-6.72)*		*<.001*		*2.62 (2.06-3.35)*		*<.001*		*2.99 (2.31-3.86)*		*<.001*		1.04 (0.78-1.38)		.81		1.30 (0.77-2.19)		.33	
	60-69	*10.35 (9.17-11.68)*		*<.001*		*4.30 (3.38-5.48)*		*<.001*		*4.91 (3.81-6.31)*		*<.001*		0.99 (0.75-1.31)		.94		1.46 (0.87-2.45)		.15	
	≥70	*12.85 (11.38-14.51)*		*<.001*		*5.69 (4.47-7.25)*		*<.001*		*6.45 (5.02-8.28)*		*<.001*		1.24 (0.95-1.64)		.12		*1.92 (1.14-3.22)*		*.01*	
**Region of residence**																					
	Urban	1.00 (reference)		—		1.00 (reference)		—		1.00 (reference)		—		1.00 (reference)		—		1.00 (reference)		—	
	Rural	*1.28 (1.19-1.37)*		*<.001*		1.03 (0.91-1.16)		0.67		1.05 (0.93-1.17)		0.45		1.06 (0.93-1.21)		0.36		1.08 (0.93-1.26)		0.34	
**BMI group^c^**																					
	Underweight	1.00 (reference)		—		1.00 (reference)		—		1.00 (reference)		—		1.00 (reference)		—		1.00 (reference)		—	
	Normal	*1.87 (1.51-2.32)*		*<.001*		0.97 (0.58-1.63)		.92		1.13 (0.72-1.76)		.60		0.81 (0.49-1.33)		.40		0.61 (0.32-1.15)		.13	
	Overweight	*2.81 (2.27-3.49)*		*<.001*		0.75 (0.45-1.26)		.28		1.00 (0.64-1.56)		.99		0.65 (0.40-1.07)		.09		0.59 (0.31-1.10)		.10	
	Obese	*4.56 (3.68-5.63)*		*<.001*		*0.52 (0.31-0.86)*		*.01*		0.76 (0.49-1.18)		.22		*0.61 (0.37-1.00)*		*.048*		*0.52 (0.27-0.98)*		*.04*	
**Educational background**																					
	Elementary school or lower	1.00 (reference)		—		1.00 (reference)		—		1.00 (reference)		—		1.00 (reference)		—		1.00 (reference)		—	
	Middle school	*0.76 (0.70-0.81)*		*<.001*		*0.74 (0.64-0.86)*		*<.001*		*0.79 (0.69-0.91)*		*.001*		0.93 (0.80-1.08)		.31		0.91 (0.75-1.09)		.28	
	High school	*0.45 (0.42-0.47)*		*<.001*		*0.54 (0.48-0.62)*		*<.001*		*0.58 (0.51-0.65)*		*<.001*		0.92 (0.80-1.05)		0.21		*0.83 (0.70-0.99)*		*0.04*	
	College or higher	*0.27 (0.25-0.29)*		*<.001*		*0.46 (0.40-0.53)*		*<.001*		*0.47 (0.42-0.54)*		*<.001*		0.97 (0.85-1.12)		0.72		1.02 (0.85-1.23)		0.84	
**Household income**																					
	Lowest quartile	1.00 (reference)		—		1.00 (reference)		—		1.00 (reference)		—		1.00 (reference)		—		1.00 (reference)		—	
	Second quartile	*0.55 (0.52-0.59)*		*<.001*		*0.70 (0.61-0.79)*		*<.001*		*0.76 (0.67-0.86)*		*<.001*		*0.84 (0.73-0.95)*		*.01*		*0.84 (0.71-0.98)*		*.03*	
	Third quartile	*0.40 (0.38-0.43)*		*<.001*		*0.60 (0.52-0.69)*		*<.001*		*0.66 (0.58-0.75)*		*<.001*		*0.86 (0.75-0.99)*		*.03*		0.85 (0.71-1.01)		.06	
	Highest quartile	*0.35 (0.33-0.38)*		*<.001*		*0.60 (0.52-0.68)*		*<.001*		*0.65 (0.57-0.74)*		*<.001*		*0.86 (0.74-1.00)*		*.04*		*0.75 (0.62-0.91)*		*.004*	
**Smoking status**																					
	Nonsmoker	1.00 (reference)		—		1.00 (reference)		—		1.00 (reference)		—		1.00 (reference)		—		1.00 (reference)		—	
	Smoker	*1.28 (1.22-1.34)*		*<.001*		*0.85 (0.78-0.94)*		*.001*		*0.81 (0.74-0.88)*		*<.001*		1.09 (0.98-1.21)		.10		1.01 (0.89-1.15)		.89	

^a^Not applicable.

^b^Numbers in italics indicate a significant difference (*P*<.05).

^c^BMI was divided into 4 groups according to Asian-Pacific guidelines: underweight (<18.5 kg/m^2^), normal (18.5-22.9 kg/m^2^), overweight (23.0-24.9 kg/m^2^), and obese (≥25 kg/m^2^).

## Discussion

### Principal Findings

Our study examined the longitudinal trends in type 2 diabetes prevalence, awareness, treatment, control among participants with diabetes, and control among participants being treated using KNHANES data spanning from 1998 to 2022, encompassing 139,786 South Koreans. Male sex, older age, higher BMI, lower educational background, and lower household income, as well as smoking behavior, were associated with a higher prevalence of type 2 diabetes. Notably, an escalating trend in prevalence and control among participants with diabetes was observed, without substantial slope change before and during the pandemic. The trajectory of awareness exhibited a U-shaped curve over the study period, with a general decline from 1998 to 2019, followed by an accelerated increase from 2020 to 2022. Concurrently, both treatment and control among participants being treated showed a steeper increase during the pandemic, displaying a discernible positive trend compared to the prepandemic period. Older adults and female participants had higher awareness and treatment, while people with higher BMI had higher prevalence but lower awareness and control. No substantial differences between rural and urban residents were found. Higher education and income levels were associated with lower awareness, treatment, and controls. These results highlight the need for tailored health policies to address the susceptible populations in managing type 2 diabetes in South Korea.

### Comparison With Previous Studies

There were several previous studies on the prevalence of diabetes in South Korea. They primarily focused on biological factors, such as sex and age, observing trends of prevalence in each population and identifying at-risk groups [11,15,23,24]. While some studies attempted to determine factors associated with prevalence using a broader range of variables, they did not extend their analysis beyond prevalence [11]. By contrast, our research used a variety of sociodemographic variables to identify factors associated with type 2 diabetes and addressed additional indicators such as awareness, treatment, and control, as well as prevalence.

Several previous studies have documented the prevalence, awareness, treatment, and control of type 2 diabetes in countries other than South Korea. In the Chinese population, similar to our result, the female sex was associated with lower prevalence yet higher awareness and treatment [25]. In a nationwide study in Iran, in contrast to our results, a higher prevalence of type 2 diabetes in female participants and urban residents was observed, and similar to our findings, higher awareness in female participants and higher treatment and control in the older adult group were observed [26]. The disparities between foreign studies and our study may stem from differences in race, cultural habit, lifestyle, and accessibility to health care.

Furthermore, it is notable that we tracked the trend and deviation of wORs throughout the pandemic using long-term, nationwide, and large population data, which enhances the reliability of the study, in contrast to the previous studies that did not include the pandemic.

### Plausible Underlying Mechanisms

The increasing trend in prevalence rate can be attributed to dietary shifts toward processed foods, compounded by the inherent vulnerability of pancreatic function in Koreans [27-29]. The higher prevalence of type 2 diabetes among male participants compared to female participants may stem from the protective effect conferred by estrogen in female participants and from the higher accumulation of visceral fat, unfavorable dietary patterns, and higher rates of smoking and harmful alcohol use in male participants [30-35]. The higher awareness and treatment rate in female participants can be attributed to more frequent health care service use, superior health literacy, and greater vigilance to diabetes due to their susceptibility to complications [36-40].

The prevalence exhibits a positive correlation with age, with the most substantial increase observed in people in their 40s and 50s. Given the chronic nature of type 2 diabetes management, this is because onset during middle age is projected into older age groups. Elevated awareness, treatment, and control among participants being treated in older age groups are likely attributable to prolonged exposure to type 2 diabetes, symptomatic presentation, increased health care use, and enhanced treatment adherence [41-43].

The U-shaped trend of awareness can be attributed to the rising prevalence of type 2 diabetes, driven by westernized dietary habits since 1998. Individuals in their 20s or 30s often experience milder symptoms and lower perceived risk, which lead to reduced awareness until the onset of moderate symptoms or diagnosis through screening [24,41]. Awareness increased in the 2010s due to heightened risk perception and a public health campaign under the National Health Plan 2020 [5].

Although people with high BMI display lower awareness, no differences in treatment are observed compared to other groups. This might be because the population with obesity has a relatively lower perceived risk [44]. Higher prevalence, lower control among participants with diabetes, and lower control among participants being treated were observed in the group with obesity, likely attributable to the heightened insulin resistance associated with fat, posing challenges to glycemic control [4,45,46]. Hence, there is a need for health policies aimed at enhancing type 2 diabetes management among the population with obesity.

Contrary to general prejudgment, individuals with higher educational backgrounds and household incomes exhibited lower levels of awareness and treatment adherence [45]. The same pattern was seen in the previous studies on the awareness of hypertension in South Korea [47]. These imply that socially privileged groups are not always more concerned about their health.

There have been remarkable increases in awareness, treatment, and control among participants being treated during the pandemic. A plausible hypothesis is that heightened public awareness of type 2 diabetes may have ensued as attention to noninfectious diseases intensified amidst the COVID-19 pandemic [48]. Moreover, previous studies have indicated that Koreans with NCDs exhibited relatively healthier lifestyle changes compared to those without NCDs during the pandemic, including improvements in physical activity, dietary habits, and sleep patterns, which could have contributed to the enhanced treatment and control outcomes [49]. In addition, unlike many other countries, South Korea adopted a relatively minimal lockdown policy, with health care facility use remaining consistent with prepandemic levels [43].

### Strengths and Limitations

Our study has several strengths. Primarily, we used the nationally representative KNHANES data set to analyze long-term trends in type 2 diabetes prevalence, awareness, treatment, and control in South Korea from 1998 to 2022. This 25-year investigation highlights the impact of the COVID-19 pandemic crisis on chronic NCDs. In addition, our study offers insights into a largely monoethnic Korean population, aiding in the formulation of future national diabetes management strategies.

However, despite these merits, it is imperative to acknowledge inherent limitations, mainly attributable to the character of the KNHANES data set. Notably, the cross-sectional nature of the KNHANES data may impede our ability to delineate causal relationships. Nonetheless, the substantial cohort size comprising 139,786 participants renders our findings compelling. Furthermore, the KNHANES data set contained very few individuals aged 0 to 29 years with diabetes, making it challenging to perform a statistically significant analysis within this age group. Consequently, we excluded individuals aged <30 years, limiting our insights in pediatric and young adult populations. Despite this, our study remains valid, as individuals aged >30 years constitute the primary demographic at risk for type 2 diabetes [14,50]. Finally, owing to the survey-based nature of KNHANES and its reliance on self-reported variables, response bias may ensue. To mitigate this bias, we incorporated objective measures, such as blood tests, to furnish more precise data.

### Clinical Policy Implications

Recent improvements in type 2 diabetes awareness, treatment, and control among participants being treated underscore the importance of sustained health care and social initiatives to uphold this positive trend. The government needs to prioritize efforts for people with type 2 diabetes who have a low educational level or low household income, although it still needs to decrease the prevalence among rural residents. Overall, in the postpandemic period, tailored policies are required to target populations that were susceptible to type 2 diabetes during the pandemic.

### Conclusions

Over the recent decade, there has been an increase in type 2 diabetes prevalence, awareness, treatment, control among participants with diabetes, and control among participants being treated. Particularly noteworthy is the steeper increase in awareness, treatment, and control among participants being treated during the pandemic. However, older populations and people with lower educational backgrounds experienced less improvement in awareness and control trends compared to younger populations and more educated people. Furthermore, individuals in the lower income group showed a disproportionate deceleration in prevalence during the pandemic. Hence, it is imperative to devise customized strategies considering sociodemographic factors specific to each subgroup. These strategies should aim to mitigate the rise in prevalence and enhance awareness, treatment, and control, which are presently suboptimal. Consequently, further investigation is warranted to investigate the specific mechanisms underlying the heterogeneous changes observed in each subgroup.

## Appendix

Multimedia Appendix 1 Serial cross-sectional type 2 diabetes prevalence, awareness, treatment, control among participants with diabetes, and control among participants being treated and their stratified value by socioeconomic factors during the periods from 1998 to 2005, from 2007 to 2009, from 2010 to 2012, from 2013 to 2015, from 2016 to 2019, and from 2020 to 2022 (the COVID-19 pandemic) in South Korea.

Multimedia Appendix 2 National trends of the prevalence, awareness, treatment, control among participants with diabetes, and control among participants being treated by age, region of residence, BMI group, educational background, household income, and smoking status among male and female participants before and during the COVID-19 pandemic based on data obtained from the Korea National Health and Nutrition Examination Survey (KNHANES).

## Abbreviations

KDCA: Korea Disease Control and Prevention Agency
KNHANES: Korea National Health and Nutrition Examination Survey
NCD: noncommunicable disease
wOR: weighted odds ratio

## Data Availability

The data sets generated and/or analyzed during this study are available from the corresponding author on reasonable request.
