# Supplementary material for: Prevalence, Awareness, Treatment, and Control of Type 2 Diabetes in South Korea (1998 to 2022): Nationwide Cross-Sectional Study
Source: JMIR Public Health Surveill. 2024 Aug 27;10:e59571. doi: 10.2196/59571 (PMC11387923; doi:10.2196/59571)
Supplement: Multimedia Appendix 1 [file publichealth_v10i1e59571_app1.docx]

**Table S1.** Serial cross-sectional type 2 diabetes prevalence, awareness, treatment, control among participants with diabetes, and control among participants being treated (weighted % [95% CI]) and their stratified value by socioeconomic factors during the periods 1998–2005, 2007–2009, 2010–2012, 2013–2015, 2016–2019, and the COVID-19 pandemic (2020–2022) in South Korea. The outcomes were derived from the KNHANES. The β coefficient represents the slope of the trend curve over time and β_diff_ is defined as the difference in β before and during the pandemic.

| Variables | Rate | Pre-pandemic | | | | | During the pandemic | Trends in the pre-pandemic,  β (95% CI) ^a^ | Trends in the pandemic,  β (95% CI) ^a^ | Trend differences,  β_diff_ (95% CI) ^a^ |
| --- | --- | --- | --- | --- | --- | --- | --- | --- | --- | --- |
|  |  | 1998–2005 | 2007–2009 | 2010–2012 | 2013–2015 | 2016–2019 | 2020–2022 |  |  |  |
| Overall | Prevalence | 6.71 (6.42 to 7.00) | 10.02 (9.40 to 10.63) | 11.64 (10.99 to 12.29) | 13.33 (12.61 to 14.06) | 14.07 (13.45 to 14.70) | 15.61 (14.83 to 16.38) | **1.74 (1.56 to 1.92)** | **1.53 (0.52 to 2.54)** | -0.21 (-1.22 to 0.80) |
|  | Awareness | 83.62 (81.74 to 85.50) | 73.80 (71.06 to 76.54) | 66.80 (64.10 to 69.50) | 64.37 (61.79 to 66.96) | 66.64 (64.64 to 68.63) | 72.56 (70.39 to 74.72) | **-3.45 (-4.20 to -2.70)** | **5.92 (2.98 to 8.86)** | **9.37 (6.43 to 12.31)** |
|  | Treatment | 46.78 (44.59 to 48.97) | 58.37 (55.58 to 61.16) | 58.63 (55.98 to 61.27) | 57.93 (55.28 to 60.58) | 61.67 (59.56 to 63.79) | 68.33 (65.95 to 70.71) | **2.29 (1.51 to 3.08)** | **6.66 (3.49 to 9.83)** | **4.37 (1.20 to 7.54)** |
|  | Control among participants with diabetes | 30.00 (26.58 to 33.42) | 28.65 (25.83 to 31.48) | 25.06 (22.32 to 27.81) | 22.51 (20.15 to 24.86) | 27.38 (25.50 to 29.25) | 29.14 (26.82 to 31.47) | -0.51 (-1.42 to 0.39) | 1.77 (-1.22 to 4.76) | 2.28 (-0.84 to 5.40) |
|  | Control among participants being treated | 24.70 (19.97 to 29.42) | 24.50 (21.13 to 27.88) | 24.84 (21.63 to 28.04) | 22.19 (19.30 to 25.08) | 26.36 (24.06 to 28.66) | 30.68 (27.88 to 33.48) | **0.41 (-0.73 to 1.54)** | **4.33 (0.70 to 7.95)** | **3.92 (0.30 to 7.55)** |
| Sex | | | | | | | | | | |
| Male | Prevalence | 7.18 (6.78 to 7.57) | 11.01 (10.12 to 11.89) | 12.88 (11.90 to 13.86) | 14.94 (13.86 to 16.02) | 15.87 (14.96 to 16.78) | 18.08 (16.90 to 19.26) | **2.06 (1.80 to 2.31)** | **2.22 (0.73 to 3.70)** | 0.16 (-1.33 to 1.65) |
|  | Awareness | 83.61 (81.36 to 85.87) | 71.29 (67.30 to 75.28) | 64.38 (60.31 to 68.45) | 62.15 (58.38 to 65.92) | 63.31 (60.52 to 66.11) | 71.04 (67.93 to 74.14) | **-3.90 (-4.96 to -2.84)** | **7.72 (3.55 to 11.89)** | **11.62 (7.45 to 15.79)** |
|  | Treatment | 42.56 (39.77 to 45.35) | 54.68 (50.45 to 58.90) | 55.82 (51.94 to 59.70) | 55.50 (51.67 to 59.33) | 58.05 (55.12 to 60.98) | 65.94 (62.50 to 69.38) | **2.39 (1.26 to 3.51)** | **7.89 (3.38 to 12.39)** | **5.50 (0.99 to 10.01)** |
|  | Control among participants with diabetes | 32.84 (27.83 to 37.84) | 30.76 (26.86 to 34.67) | 25.61 (21.76 to 29.47) | 24.06 (20.93 to 27.19) | 28.09 (25.47 to 30.72) | 29.44 (26.33 to 32.56) | -0.84 (-2.11 to 0.43) | 1.35 (-2.74 to 5.44) | 2.19 (-2.09 to 6.48) |
|  | Control among participants being treated | 26.18 (18.80 to 33.56) | 25.90 (20.95 to 30.84) | 23.10 (18.98 to 27.21) | 23.12 (19.11 to 27.13) | 26.28 (23.11 to 29.46) | 30.17 (26.43 to 33.90) | 0.27 (-1.36 to 1.90) | 3.88 (-1.03 to 8.79) | 3.61 (-1.30 to 8.52) |
| Female | Prevalence | 6.28 (5.91 to 6.64) | 9.06 (8.29 to 9.82) | 10.45 (9.65 to 11.26) | 11.84 (11.00 to 12.68) | 12.35 (11.61 to 13.09) | 13.18 (12.23 to 14.12) | **1.44 (1.23 to 1.65)** | 0.83 (-0.39 to 2.04) | -0.61 (-1.83 to 0.61) |
|  | Awareness | 83.63 (81.30 to 85.97) | 76.76 (73.25 to 80.26) | 69.66 (66.05 to 73.26) | 66.97 (63.70 to 70.25) | 70.74 (68.08 to 73.39) | 74.60 (71.79 to 77.41) | **-2.85 (-3.81 to -1.89)** | **3.86 (0.01 to 7.73)** | **6.71 (2.85 to 10.57)** |
|  | Treatment | 51.31 (48.38 to 54.24) | 62.73 (58.81 to 66.64) | 61.93 (58.14 to 65.72) | 60.77 (57.43 to 64.11) | 66.14 (63.32 to 68.97) | 71.55 (68.61 to 74.48) | **2.27 (1.22 to 3.32)** | **5.40 (1.33 to 9.47)** | 3.13 (-0.94 to 7.20) |
|  | Control among participants with diabetes | 27.14 (22.96 to 31.32) | 25.98 (22.07 to 29.89) | 24.39 (20.75 to 28.02) | 20.58 (17.45 to 23.71) | 26.48 (23.94 to 29.01) | 28.74 (25.62 to 31.85) | -0.14 (-1.34 to 1.07) | 2.26 (-1.75 to 6.28) | 2.40 (-1.80 to 6.59) |
|  | Control among participants being treated | 23.49 (17.41 to 29.57) | 23.01 (18.70 to 27.32) | 26.81 (21.96 to 31.65) | 21.11 (17.05 to 25.18) | 26.44 (23.40 to 29.48) | 31.33 (27.48 to 35.18) | 0.55 (-0.92 to 2.03) | 4.89 (-0.02 to 9.81) | 4.34 (-0.58 to 9.26) |
| Age, years | | | | | | | | | | |
| 30–39 | Prevalence | 1.36 (1.12 to 1.59) | 2.57 (1.93 to 3.22) | 2.89 (2.21 to 3.58) | 2.59 (1.91 to 3.28) | 2.82 (2.19 to 3.45) | 3.62 (2.67 to 4.57) | **0.29 (0.12 to 0.45)** | 0.80 (-0.35 to 1.94) | 0.51 (-0.64 to 1.66) |
|  | Awareness | 63.56 (55.08 to 72.05) | 43.43 (31.74 to 55.12) | 36.94 (25.00 to 48.87) | 39.68 (26.79 to 52.56) | 32.25 (22.48 to 42.02) | 55.47 (42.06 to 68.89) | **-5.50 (-8.99 to -2.01)** | **23.23 (6.64 to 39.81)** | **28.73 (12.15 to 45.32)** |
|  | Treatment | 24.25 (16.52 to 31.99) | 22.77 (13.04 to 32.49) | 27.50 (16.38 to 38.63) | 29.58 (17.57 to 41.58) | 27.00 (17.65 to 36.35) | 47.59 (34.63 to 60.55) | 1.30 (-1.85 to 4.44) | **20.59 (4.61 to 36.57)** | **19.29 (3.31 to 35.27)** |
|  | Control among participants with diabetes | 50.80 (38.60 to 63.00) | 30.35 (17.72 to 42.98) | 19.32 (10.11 to 28.52) | 22.29 (10.71 to 33.88) | 30.81 (20.32 to 41.31) | 25.53 (13.65 to 37.41) | -1.37 (-5.66 to 2.92) | -5.29 (-21.15 to 10.58) | -3.92 (-20.35 to 12.52) |
|  | Control among participants being treated | 44.98 (3.38 to 86.59) | 23.46 (2.55 to 44.36) | 9.91 (0.00 to 23.19) | 12.81 (0.00 to 29.32) | 34.02 (13.67 to 54.37) | 17.58 (2.45 to 32.70) | 2.66 (-5.75 to 11.08) | -16.44 (-41.81 to 8.93) | -19.10 (-44.47 to 6.27) |
| 40–49 | Prevalence | 4.23 (3.83 to 4.63) | 6.55 (5.57 to 7.52) | 6.95 (5.78 to 8.13) | 8.36 (7.20 to 9.53) | 8.05 (7.00 to 9.09) | 8.21 (6.99 to 9.43) | **0.90 (0.62 to 1.19)** | 0.16 (-1.45 to 1.77) | -0.74 (-2.35 to 0.87) |
|  | Awareness | 75.78 (71.78 to 79.79) | 58.68 (51.09 to 66.27) | 46.83 (39.32 to 54.33) | 45.85 (38.25 to 53.45) | 45.75 (39.45 to 52.05) | 57.61 (49.95 to 65.27) | **-6.15 (-8.29 to -4.01)** | **11.86 (1.96 to 21.76)** | **18.01 (8.11 to 27.91)** |
|  | Treatment | 35.52 (31.02 to 40.02) | 41.74 (34.77 to 48.71) | 37.92 (30.53 to 45.31) | 35.71 (28.31 to 43.10) | 40.05 (33.82 to 46.27) | 52.78 (45.18 to 60.38) | 0.03 (-2.05 to 2.12) | **12.74 (2.93 to 22.54)** | **12.71 (2.91 to 22.52)** |
|  | Control among participants with diabetes | 30.95 (24.20 to 37.71) | 26.31 (19.36 to 33.25) | 22.88 (16.01 to 29.74) | 23.65 (16.92 to 30.37) | 22.62 (17.70 to 27.54) | 22.54 (16.33 to 28.75) | -1.31 (-3.55 to 0.93) | -0.08 (-8.01 to 7.85) | 1.23 (-7.01 to 9.47) |
|  | Control among participants being treated | 9.18 (0.00 to 19.52) | 21.73 (11.29 to 32.17) | 19.45 (9.81 to 29.09) | 19.93 (9.88 to 29.97) | 13.08 (6.95 to 19.21) | 25.73 (15.84 to 35.62) | -2.02 (-5.44 to 1.41) | **12.65 (1.00 to 24.31)** | **14.67 (3.02 to 26.33)** |
| 50–59 | Prevalence | 9.74 (9.00 to 10.47) | 12.74 (11.37 to 14.12) | 14.32 (12.90 to 15.74) | 13.51 (12.09 to 14.92) | 15.12 (13.91 to 16.33) | 17.69 (16.03 to 19.34) | **1.03 (0.65 to 1.42)** | **2.57 (0.52 to 4.62)** | 1.54 (-0.51 to 3.59) |
|  | Awareness | 87.12 (84.32 to 89.93) | 73.98 (68.53 to 79.44) | 67.22 (61.75 to 72.69) | 57.35 (51.94 to 62.77) | 62.79 (58.54 to 67.05) | 67.58 (62.93 to 72.22) | **-5.43 (-6.95 to -3.91)** | 4.78 (-1.52 to 11.09) | **10.21 (3.91 to 16.52)** |
|  | Treatment | 48.43 (44.46 to 52.41) | 57.12 (51.23 to 63.00) | 58.98 (53.36 to 64.61) | 51.63 (46.13 to 57.13) | 55.93 (51.53 to 60.32) | 63.78 (58.71 to 68.84) | 0.27 (-1.35 to 1.89) | **7.85 (1.14 to 14.57)** | **7.58 (0.87 to 14.30)** |
|  | Control among participants with diabetes | 25.67 (19.39 to 31.95) | 25.54 (19.75 to 31.34) | 25.19 (19.56 to 30.82) | 22.98 (17.88 to 28.09) | 27.65 (23.89 to 31.42) | 28.99 (24.22 to 33.75) | 0.51 (-1.35 to 2.38) | 1.33 (-4.75 to 7.42) | 0.82 (-5.55 to 7.19) |
|  | Control among participants being treated | 22.42 (14.07 to 30.77) | 18.66 (12.07 to 25.25) | 23.27 (16.26 to 30.28) | 20.99 (14.06 to 27.93) | 23.56 (18.69 to 28.44) | 28.44 (22.31 to 34.56) | 1.01 (-1.31 to 3.34) | 4.87 (-2.97 to 12.72) | 3.86 (-3.99 to 11.71) |
| 60–69 | Prevalence | 15.11 (14.11 to 16.12) | 21.56 (19.76 to 23.36) | 22.03 (20.40 to 23.67) | 26.13 (24.12 to 28.14) | 23.26 (21.73 to 24.79) | 23.63 (21.93 to 25.33) | **1.82 (1.34 to 2.30)** | 0.37 (-1.92 to 2.66) | -1.45 (-3.74 to 0.84) |
|  | Awareness | 86.60 (83.94 to 89.27) | 86.22 (82.77 to 89.67) | 76.31 (72.41 to 80.21) | 70.99 (67.09 to 74.89) | 73.05 (69.85 to 76.25) | 75.93 (72.26 to 79.60) | **-4.11 (-5.19 to -3.02)** | 2.87 (-1.99 to 7.74) | **6.98 (2.12 to 11.85)** |
|  | Treatment | 49.28 (45.64 to 52.92) | 72.29 (68.18 to 76.41) | 68.96 (64.71 to 73.21) | 65.41 (61.32 to 69.51) | 69.37 (66.08 to 72.66) | 71.40 (67.48 to 75.32) | **2.01 (0.80 to 3.23)** | 2.04 (-3.08 to 7.16) | 0.03 (-5.09 to 5.15) |
|  | Control among participants with diabetes | 24.59 (19.05 to 30.13) | 30.45 (25.46 to 35.44) | 21.86 (17.77 to 25.95) | 20.17 (16.58 to 23.76) | 26.48 (23.32 to 29.64) | 28.82 (24.91 to 32.74) | -0.75 (-2.28 to 0.79) | 2.35 (-2.67 to 7.36) | 3.09 (-2.16 to 8.34) |
|  | Control among participants being treated | 25.53 (17.95 to 33.11) | 28.23 (22.91 to 33.56) | 22.37 (17.07 to 27.67) | 21.70 (17.16 to 26.23) | 25.90 (21.98 to 29.83) | 30.39 (25.80 to 34.98) | -0.48 (-2.32 to 1.36) | 4.48 (-1.60 to 10.57) | 4.96 (-1.13 to 11.05) |
| ≥70 | Prevalence | 14.65 (13.48 to 15.83) | 19.66 (17.66 to 21.66) | 24.92 (22.97 to 26.87) | 28.38 (26.15 to 30.62) | 29.86 (28.22 to 31.50) | 30.18 (28.37 to 32.00) | **3.73 (3.18 to 4.27)** | 0.32 (-2.12 to 2.77) | -3.41 (-5.86 to -0.97) |
|  | Awareness | 88.11 (85.10 to 91.12) | 81.01 (76.58 to 85.44) | 77.49 (73.56 to 81.43) | 79.19 (74.99 to 83.39) | 79.45 (76.81 to 82.10) | 83.51 (80.75 to 86.28) | -1.09 (-2.22 to 0.03) | **4.06 (0.22 to 7.89)** | **5.15 (1.32 to 8.99)** |
|  | Treatment | 58.27 (53.88 to 62.66) | 68.59 (63.50 to 73.68) | 69.36 (65.37 to 73.36) | 74.02 (69.74 to 78.31) | 75.44 (72.54 to 78.34) | 80.07 (76.96 to 83.17) | **3.39 (2.11 to 4.67)** | **4.63 (0.38 to 8.87)** | 1.24 (-3.01 to 5.49) |
|  | Control among participants with diabetes | 33.74 (26.69 to 40.79) | 32.00 (26.38 to 37.62) | 31.67 (26.94 to 36.40) | 24.03 (20.15 to 27.91) | 29.88 (26.72 to 33.03) | 33.23 (29.41 to 37.04) | -1.07 (-2.75 to 0.60) | 3.35 (-1.61 to 8.31) | 4.42 (-0.81 to 9.66) |
|  | Control among participants being treated | 31.40 (21.03 to 41.76) | 27.43 (21.45 to 33.41) | 33.19 (27.48 to 38.90) | 25.26 (20.81 to 29.71) | 31.96 (28.31 to 35.61) | 35.54 (31.23 to 39.85) | 0.58 (-1.38 to 2.54) | 3.58 (-2.08 to 9.24) | 3.00 (-2.66 to 8.66) |
| Region of residence | | | | | | | | | | |
| Urban | Prevalence | 6.33 (6.02 to 6.65) | 9.87 (9.15 to 10.60) | 11.02 (10.31 to 11.72) | 13.05 (12.26 to 13.84) | 13.14 (12.49 to 13.80) | 14.90 (14.04 to 15.76) | **1.59 (1.40 to 1.79)** | **1.76 (0.67 to 2.85)** | 0.17 (-0.92 to 1.26) |
|  | Awareness | 84.19 (82.08 to 86.30) | 73.35 (70.09 to 76.61) | 66.77 (63.66 to 69.88) | 65.13 (62.32 to 67.93) | 66.04 (63.74 to 68.33) | 72.36 (69.88 to 74.84) | **-3.53 (-4.40 to -2.66)** | **6.32 (2.95 to 9.69)** | **9.85 (6.48 to 13.22)** |
|  | Treatment | 46.37 (43.86 to 48.88) | 57.90 (54.64 to 61.17) | 57.79 (54.64 to 60.93) | 58.60 (55.69 to 61.51) | 61.23 (58.86 to 63.60) | 67.99 (65.21 to 70.77) | **2.37 (1.47 to 3.28)** | **6.76 (3.11 to 10.40)** | **4.39 (0.74 to 8.04)** |
|  | Control among participants with diabetes | 30.05 (25.99 to 34.10) | 28.12 (24.82 to 31.42) | 25.05 (21.77 to 28.33) | 22.84 (20.34 to 25.33) | 27.03 (24.99 to 29.07) | 28.51 (25.83 to 31.19) | -0.48 (-1.52 to 0.56) | 1.48 (-1.89 to 4.85) | 1.96 (-1.56 to 5.49) |
|  | Control among participants being treated | 26.29 (20.54 to 32.05) | 23.56 (19.65 to 27.47) | 25.02 (21.16 to 28.88) | 22.63 (19.38 to 25.89) | 25.88 (23.38 to 28.37) | 30.00 (26.69 to 33.31) | 0.42 (-0.87 to 1.72) | 4.13 (-0.02 to 8.27) | 3.71 (-0.44 to 7.86) |
| Rural | Prevalence | 8.19 (7.47 to 8.90) | 10.55 (9.39 to 11.72) | 13.89 (12.21 to 15.58) | 14.57 (12.61 to 16.53) | 18.89 (17.04 to 20.74) | 19.16 (17.35 to 20.97) | **2.54 (2.08 to 3.01)** | 0.27 (-2.34 to 2.88) | -2.27 (-4.88 to 0.34) |
|  | Awareness | 81.91 (77.88 to 85.94) | 75.37 (70.66 to 80.07) | 66.90 (61.57 to 72.24) | 61.41 (55.08 to 67.74) | 68.78 (64.76 to 72.81) | 73.33 (68.94 to 77.73) | **-3.18 (-4.66 to -1.71)** | 4.55 (-1.41 to 10.51) | **7.73 (1.77 to 13.69)** |
|  | Treatment | 48.03 (43.56 to 52.50) | 60.01 (54.67 to 65.34) | 61.04 (56.24 to 65.84) | 55.30 (48.94 to 61.66) | 63.26 (58.64 to 67.88) | 69.68 (65.38 to 73.98) | **2.06 (0.43 to 3.68)** | **6.42 (0.12 to 12.72)** | 4.36 (-1.94 to 10.66) |
|  | Control among participants with diabetes | 29.87 (23.52 to 36.23) | 30.58 (25.25 to 35.91) | 25.10 (20.19 to 30.00) | 21.19 (14.99 to 27.40) | 28.66 (24.16 to 33.16) | 31.69 (27.22 to 36.15) | -0.60 (-2.48 to 1.28) | 3.03 (-3.35 to 9.40) | 3.63 (-3.01 to 10.27) |
|  | Control among participants being treated | 21.01 (12.97 to 29.06) | 27.74 (21.27 to 34.21) | 24.35 (18.82 to 29.88) | 20.26 (14.08 to 26.45) | 28.09 (22.61 to 33.57) | 33.36 (28.37 to 38.35) | 0.41 (-1.95 to 2.78) | 5.27 (-2.16 to 12.71) | 4.86 (-2.58 to 12.30) |
| BMI group ^b^ | | | | | | | | | | |
| Underweight | Prevalence | 8.15 (5.27 to 11.03) | 4.41 (2.42 to 6.39) | 5.59 (3.12 to 8.05) | 4.74 (2.79 to 6.69) | 3.91 (2.28 to 5.53) | 5.63 (2.93 to 8.33) | -0.44 (-1.15 to 0.26) | 1.73 (-1.43 to 4.88) | 2.17 (-0.99 to 5.33) |
|  | Awareness | 56.01 (37.13 to 74.89) | 83.93 (66.23 to 100.00) | 83.43 (62.78 to 100.00) | 61.30 (38.29 to 84.31) | 67.47 (46.11 to 88.83) | 86.71 (73.65 to 99.78) | -3.25 (-10.43 to 3.93) | 19.24 (-5.80 to 44.27) | 22.49 (-2.55 to 47.53) |
|  | Treatment | 30.71 (14.33 to 47.09) | 62.76 (40.90 to 84.61) | 60.40 (36.37 to 84.43) | 49.57 (27.33 to 71.81) | 62.61 (40.95 to 84.26) | 81.76 (66.72 to 96.80) | 2.23 (-5.25 to 9.70) | 19.15 (-7.21 to 45.51) | 16.92 (-9.44 to 43.28) |
|  | Control among participants with diabetes | 47.80 (23.07 to 72.53) | 32.99 (10.35 to 55.63) | 37.71 (15.89 to 59.53) | 20.79 (3.44 to 38.13) | 42.68 (20.93 to 64.42) | 37.02 (8.85 to 65.19) | -0.44 (-8.32 to 7.43) | -5.66 (-41.25 to 29.94) | -5.21 (-41.67 to 31.25) |
|  | Control among participants being treated | 20.94 (0.00 to 57.25) | 25.49 (0.00 to 54.36) | 44.18 (14.52 to 73.84) | 32.82 (3.88 to 61.76) | 47.24 (20.03 to 74.44) | 38.91 (5.79 to 72.02) | 5.64 (-5.47 to 16.75) | -8.33 (-51.21 to 34.55) | -13.97 (-56.85 to 28.91) |
| Normal | Prevalence | 6.36 (5.52 to 7.20) | 6.00 (5.25 to 6.74) | 8.23 (7.35 to 9.10) | 9.00 (8.11 to 9.88) | 8.94 (8.21 to 9.67) | 10.66 (9.66 to 11.66) | **0.89 (0.61 to 1.18)** | **1.72 (0.48 to 2.97)** | 0.83 (-0.42 to 2.08) |
|  | Awareness | 57.54 (51.06 to 64.03) | 79.50 (74.23 to 84.77) | 76.95 (72.22 to 81.67) | 73.00 (67.85 to 78.15) | 72.74 (68.88 to 76.59) | 81.60 (78.02 to 85.18) | -0.84 (-2.58 to 0.91) | **8.87 (3.60 to 14.13)** | **9.71 (4.45 to 14.98)** |
|  | Treatment | 47.34 (40.62 to 54.05) | 59.77 (54.09 to 65.46) | 66.07 (60.81 to 71.33) | 62.01 (56.52 to 67.50) | 65.63 (61.58 to 69.69) | 76.74 (72.78 to 80.70) | **2.02 (0.15 to 3.88)** | **11.11 (5.44 to 16.78)** | **9.09 (3.42 to 14.76)** |
|  | Control among participants with diabetes | 37.17 (30.45 to 43.90) | 28.19 (22.66 to 33.73) | 31.12 (25.48 to 36.75) | 26.06 (21.51 to 30.60) | 31.05 (27.06 to 35.03) | 33.48 (28.82 to 38.15) | -0.19 (-2.03 to 1.65) | 2.44 (-3.69 to 8.56) | 2.63 (-3.77 to 9.02) |
|  | Control among participants being treated | 28.12 (19.02 to 37.22) | 24.67 (18.51 to 30.83) | 25.60 (19.59 to 31.61) | 23.77 (18.15 to 29.39) | 27.40 (22.89 to 31.90) | 33.35 (27.94 to 38.77) | 0.52 (-1.62 to 2.65) | 5.95 (-1.08 to 12.99) | 5.43 (-1.61 to 12.47) |
| Overweight | Prevalence | 10.68 (9.46 to 11.91) | 9.72 (8.64 to 10.80) | 10.79 (9.63 to 11.95) | 13.80 (12.49 to 15.12) | 13.39 (12.32 to 14.46) | 13.93 (12.56 to 15.31) | **1.23 (0.82 to 1.64)** | 0.54 (-1.21 to 2.29) | -0.69 (-2.44 to 1.06) |
|  | Awareness | 57.16 (50.62 to 63.70) | 77.29 (71.84 to 82.75) | 68.96 (63.94 to 73.98) | 64.21 (59.24 to 69.18) | 70.42 (66.44 to 74.39) | 78.01 (73.78 to 82.25) | -0.90 (-2.68 to 0.88) | **7.60 (1.79 to 13.40)** | **8.50 (2.70 to 14.31)** |
|  | Treatment | 42.46 (35.90 to 49.01) | 57.95 (51.95 to 63.95) | 59.44 (54.22 to 64.66) | 58.38 (53.59 to 63.17) | 66.40 (62.20 to 70.61) | 74.61 (70.24 to 78.98) | **3.40 (1.50 to 5.30)** | **8.20 (2.15 to 14.26)** | 4.80 (-1.26 to 10.86) |
|  | Control among participants with diabetes | 30.25 (23.52 to 36.98) | 29.64 (23.66 to 35.62) | 26.12 (20.69 to 31.54) | 19.04 (14.88 to 23.20) | 24.75 (21.00 to 28.50) | 32.43 (27.27 to 37.59) | **-1.92 (-3.75 to -0.09)** | **7.68 (1.30 to 14.06)** | **9.60 (2.96 to 16.24)** |
|  | Control among participants being treated | 18.25 (9.37 to 27.12) | 24.53 (17.46 to 31.60) | 27.16 (20.55 to 33.77) | 19.77 (14.72 to 24.82) | 25.39 (20.82 to 29.96) | 35.05 (29.12 to 40.98) | -0.02 (-2.35 to 2.31) | **9.66 (2.18 to 17.14)** | **9.68 (2.20 to 17.16)** |
| Obese | Prevalence | 14.48 (13.18 to 15.78) | 15.01 (13.84 to 16.18) | 16.51 (15.24 to 17.77) | 18.59 (17.27 to 19.90) | 20.51 (19.44 to 21.58) | 21.15 (19.76 to 22.53) | **1.76 (1.33 to 2.19)** | 0.64 (-1.11 to 2.40) | -1.12 (-2.88 to 0.64) |
|  | Awareness | 54.04 (49.19 to 58.89) | 69.64 (65.69 to 73.60) | 59.52 (55.53 to 63.51) | 59.91 (56.11 to 63.70) | 62.26 (59.45 to 65.07) | 65.57 (62.26 to 68.87) | -0.98 (-2.29 to 0.33) | 3.31 (-1.02 to 7.63) | 4.29 (-0.04 to 8.62) |
|  | Treatment | 41.17 (36.39 to 45.95) | 58.15 (53.98 to 62.32) | 53.82 (49.85 to 57.79) | 55.72 (51.84 to 59.61) | 57.78 (54.88 to 60.69) | 61.41 (57.84 to 64.98) | 1.15 (-0.21 to 2.51) | 3.63 (-0.97 to 8.22) | 2.48 (-2.12 to 7.08) |
|  | Control among participants with diabetes | 25.05 (20.47 to 29.63) | 27.59 (23.74 to 31.45) | 20.96 (17.36 to 24.56) | 22.43 (18.82 to 26.05) | 26.55 (24.06 to 29.05) | 25.36 (22.46 to 28.26) | 0.16 (-1.07 to 1.40) | -1.20 (-5.03 to 2.64) | -1.36 (-5.39 to 2.67) |
|  | Control among participants being treated | 25.02 (17.75 to 32.30) | 24.32 (19.24 to 29.41) | 22.33 (17.89 to 26.77) | 22.34 (18.00 to 26.67) | 25.96 (22.80 to 29.12) | 26.47 (22.87 to 30.06) | 0.58 (-1.07 to 2.23) | 0.50 (-4.30 to 5.31) | -0.08 (-4.89 to 4.73) |
| Educational background | | | | | | | | | | |
| Elementary school or lower | Prevalence | 12.25 (11.56 to 12.93) | 18.10 (16.67 to 19.53) | 21.16 (19.54 to 22.78) | 25.43 (23.45 to 27.40) | 27.27 (25.61 to 28.92) | 28.95 (26.99 to 30.91) | **3.74 (3.30 to 4.18)** | 1.68 (-0.89 to 4.25) | -2.06 (-4.63 to 0.51) |
|  | Awareness | 85.64 (83.21 to 88.07) | 79.26 (75.68 to 82.84) | 75.08 (71.51 to 78.65) | 72.57 (68.69 to 76.44) | 76.15 (73.30 to 79.01) | 83.17 (79.95 to 86.39) | **-2.18 (-3.19 to -1.18)** | **7.02 (2.69 to 11.34)** | **9.20 (4.88 to 13.53)** |
|  | Treatment | 49.81 (46.73 to 52.90) | 65.53 (61.62 to 69.44) | 68.28 (64.72 to 71.85) | 66.75 (62.74 to 70.75) | 71.94 (68.78 to 75.11) | 79.60 (76.16 to 83.05) | **3.82 (2.72 to 4.93)** | **7.66 (2.97 to 12.35)** | 3.84 (-0.85 to 8.53) |
|  | Control among participants with diabetes | 28.13 (23.45 to 32.81) | 28.10 (24.00 to 32.20) | 27.81 (23.90 to 31.73) | 20.80 (16.86 to 24.75) | 29.25 (25.89 to 32.60) | 31.54 (27.39 to 35.69) | -0.32 (-1.73 to 1.09) | 2.29 (-3.07 to 7.66) | 2.61 (-2.93 to 8.16) |
|  | Control among participants being treated | 24.79 (18.60 to 30.99) | 23.85 (19.48 to 28.23) | 29.71 (24.86 to 34.56) | 20.63 (16.09 to 25.16) | 28.86 (24.88 to 32.83) | 32.86 (28.16 to 37.57) | 0.66 (-1.02 to 2.33) | 4.01 (-2.17 to 10.19) | 3.35 (-2.83 to 9.53) |
| Middle school | Prevalence | 8.05 (7.30 to 8.79) | 12.30 (10.74 to 13.86) | 16.22 (14.27 to 18.17) | 18.86 (16.72 to 21.00) | 22.94 (21.06 to 24.82) | 24.93 (22.53 to 27.33) | **3.62 (3.13 to 4.12)** | 1.99 (-1.06 to 5.04) | -1.63 (-4.68 to 1.42) |
|  | Awareness | 84.38 (80.62 to 88.13) | 80.17 (74.29 to 86.06) | 67.67 (60.88 to 74.47) | 65.52 (59.41 to 71.62) | 71.64 (67.15 to 76.13) | 73.83 (68.48 to 79.17) | **-3.12 (-4.78 to -1.46)** | 2.19 (-4.82 to 9.19) | 5.31 (-1.70 to 12.32) |
|  | Treatment | 47.98 (43.00 to 52.96) | 63.93 (56.47 to 71.39) | 61.08 (54.18 to 67.98) | 61.05 (54.90 to 67.21) | 66.55 (61.96 to 71.13) | 70.83 (65.33 to 76.33) | **2.69 (0.83 to 4.56)** | 4.28 (-2.91 to 11.47) | 1.59 (-5.60 to 8.78) |
|  | Control among participants with diabetes | 32.26 (24.32 to 40.21) | 33.76 (26.51 to 41.01) | 23.37 (16.85 to 29.90) | 23.32 (17.66 to 28.99) | 27.80 (23.57 to 32.03) | 24.28 (19.24 to 29.32) | -1.31 (-3.50 to 0.89) | -3.53 (-10.11 to 3.06) | -2.22 (-9.16 to 4.72) |
|  | Control among participants being treated | 26.58 (14.96 to 38.21) | 29.43 (20.34 to 38.52) | 24.57 (16.47 to 32.66) | 23.10 (16.09 to 30.11) | 26.80 (21.87 to 31.72) | 25.28 (19.33 to 31.23) | -0.55 (-3.36 to 2.26) | -1.52 (-9.24 to 6.21) | -0.97 (-8.70 to 6.76) |
| High school | Prevalence | 4.57 (4.18 to 4.96) | 7.37 (6.49 to 8.25) | 9.47 (8.41 to 10.54) | 11.63 (10.50 to 12.76) | 14.34 (13.28 to 15.41) | 16.84 (15.52 to 18.16) | **2.38 (2.10 to 2.65)** | **2.50 (0.78 to 4.21)** | 0.12 (-1.60 to 1.84) |
|  | Awareness | 80.67 (77.04 to 84.29) | 66.88 (61.53 to 72.23) | 60.63 (55.19 to 66.06) | 59.62 (54.67 to 64.57) | 64.07 (60.33 to 67.82) | 69.28 (65.23 to 73.34) | **-2.51 (-3.96 to -1.06)** | 5.21 (-0.28 to 10.70) | **7.72 (2.23 to 13.21)** |
|  | Treatment | 41.95 (37.91 to 45.98) | 51.85 (46.12 to 57.58) | 51.09 (45.80 to 56.37) | 53.90 (48.84 to 58.96) | 58.25 (54.44 to 62.06) | 65.43 (61.22 to 69.64) | **3.14 (1.64 to 4.64)** | **7.18 (1.54 to 12.83)** | 4.04 (-1.61 to 9.69) |
|  | Control among participants with diabetes | 33.30 (26.34 to 40.26) | 26.32 (20.80 to 31.84) | 23.76 (18.52 to 29.00) | 23.21 (18.65 to 27.78) | 25.25 (22.06 to 28.44) | 29.33 (24.97 to 33.68) | -0.60 (-2.28 to 1.08) | 4.08 (-1.34 to 9.49) | 4.68 (-0.99 to 10.34) |
|  | Control among participants being treated | 26.23 (14.42 to 38.03) | 20.82 (13.64 to 28.00) | 19.67 (13.85 to 25.49) | 22.45 (16.90 to 28.00) | 21.85 (17.97 to 25.72) | 30.34 (24.90 to 35.78) | 0.36 (-1.84 to 2.56) | **8.49 (1.79 to 15.20)** | **8.13 (1.43 to 14.84)** |
| College or higher | Prevalence | 3.46 (3.06 to 3.86) | 5.82 (4.96 to 6.68) | 6.09 (5.23 to 6.96) | 7.39 (6.57 to 8.22) | 7.44 (6.79 to 8.09) | 9.89 (8.92 to 10.86) | **0.85 (0.62 to 1.07)** | **2.44 (1.28 to 3.61)** | **1.59 (0.43 to 2.76)** |
|  | Awareness | 81.13 (76.74 to 85.52) | 64.83 (57.12 to 72.54) | 57.36 (49.98 to 64.75) | 56.42 (50.42 to 62.41) | 54.76 (50.42 to 59.10) | 67.90 (63.50 to 72.30) | **-4.38 (-6.36 to -2.40)** | **13.14 (6.97 to 19.32)** | **17.52 (11.35 to 23.70)** |
|  | Treatment | 43.76 (38.00 to 49.53) | 45.53 (38.13 to 52.94) | 46.71 (39.40 to 54.03) | 46.51 (40.70 to 52.31) | 50.09 (45.74 to 54.44) | 62.25 (57.32 to 67.18) | 1.46 (-0.52 to 3.43) | **12.16 (5.60 to 18.73)** | **10.70 (4.14 to 17.27)** |
|  | Control among participants with diabetes | 27.20 (17.93 to 36.48) | 28.55 (20.96 to 36.13) | 23.12 (16.55 to 29.69) | 23.48 (18.61 to 28.34) | 27.64 (23.60 to 31.69) | 29.49 (25.12 to 33.87) | 0.18 (-2.10 to 2.45) | 1.85 (-4.11 to 7.81) | 1.67 (-4.70 to 8.05) |
|  | Control among participants being treated | 19.20 (6.48 to 31.91) | 25.92 (15.92 to 35.92) | 19.72 (11.97 to 27.46) | 24.10 (16.81 to 31.38) | 28.67 (22.77 to 34.56) | 31.91 (26.31 to 37.51) | 1.86 (-1.31 to 5.02) | 3.24 (-4.86 to 11.35) | 1.38 (-6.73 to 9.49) |
| Household income | | | | | | | | | | |
| Lowest quartile | Prevalence | 10.39 (9.70 to 11.08) | 17.38 (15.81 to 18.96) | 20.13 (18.37 to 21.89) | 23.78 (21.80 to 25.76) | 25.91 (24.33 to 27.49) | 26.41 (24.57 to 28.25) | **3.73 (3.30 to 4.16)** | 0.51 (-1.92 to 2.93) | **-3.22 (-5.65 to -0.80)** |
|  | Awareness | 86.17 (83.35 to 88.99) | 81.10 (76.74 to 85.45) | 73.78 (69.86 to 77.71) | 72.64 (68.00 to 77.28) | 73.16 (70.08 to 76.24) | 78.54 (74.99 to 82.09) | **-3.01 (-4.14 to -1.88)** | **5.38 (0.69 to 10.07)** | **8.39 (3.70 to 13.08)** |
|  | Treatment | 47.92 (44.30 to 51.54) | 65.81 (60.96 to 70.67) | 66.21 (62.29 to 70.12) | 66.75 (61.96 to 71.54) | 66.60 (63.20 to 70.00) | 73.61 (69.79 to 77.42) | **2.78 (1.50 to 4.05)** | **7.01 (1.90 to 12.11)** | 4.23 (-0.87 to 9.34) |
|  | Control among participants with diabetes | 28.27 (22.92 to 33.63) | 32.03 (27.07 to 36.99) | 29.66 (24.98 to 34.34) | 23.25 (18.81 to 27.69) | 29.82 (26.54 to 33.10) | 30.68 (26.67 to 34.68) | -0.77 (-2.33 to 0.78) | 0.86 (-4.33 to 6.04) | 1.63 (-3.79 to 7.04) |
|  | Control among participants being treated | 25.75 (18.30 to 33.20) | 27.81 (22.45 to 33.16) | 29.58 (23.85 to 35.31) | 23.56 (18.63 to 28.50) | 30.39 (26.24 to 34.55) | 32.47 (27.98 to 36.97) | 0.46 (-1.45 to 2.37) | 2.08 (-4.06 to 8.22) | 1.62 (-4.52 to 7.76) |
| Second quartile | Prevalence | 6.51 (5.97 to 7.05) | 11.10 (9.87 to 12.33) | 10.99 (9.91 to 12.08) | 15.26 (13.83 to 16.68) | 14.98 (13.85 to 16.10) | 17.69 (16.23 to 19.15) | **2.03 (1.70 to 2.36)** | **2.71 (0.86 to 4.57)** | 0.68 (-1.18 to 2.54) |
|  | Awareness | 84.47 (81.33 to 87.61) | 73.44 (68.17 to 78.72) | 67.32 (62.39 to 72.25) | 65.11 (60.31 to 69.90) | 65.52 (61.66 to 69.38) | 70.88 (66.78 to 74.98) | **-3.71 (-5.16 to -2.26)** | 5.37 (-0.27 to 11.01) | **9.08 (3.44 to 14.72)** |
|  | Treatment | 47.31 (43.10 to 51.51) | 57.79 (52.67 to 62.91) | 58.81 (53.88 to 63.75) | 58.72 (53.68 to 63.76) | 61.54 (57.45 to 65.62) | 67.61 (63.37 to 71.86) | **2.25 (0.73 to 3.77)** | **6.08 (0.17 to 11.98)** | 3.83 (-2.08 to 9.74) |
|  | Control among participants with diabetes | 32.58 (25.96 to 39.21) | 27.78 (22.75 to 32.82) | 24.80 (19.56 to 30.04) | 17.99 (14.16 to 21.82) | 26.58 (23.09 to 30.08) | 29.35 (25.04 to 33.66) | -1.06 (-2.72 to 0.61) | 2.77 (-2.80 to 8.33) | 3.82 (-1.99 to 9.63) |
|  | Control among participants being treated | 25.08 (15.45 to 34.71) | 25.75 (19.33 to 32.17) | 24.25 (18.08 to 30.41) | 17.28 (12.98 to 21.59) | 26.53 (22.25 to 30.81) | 31.26 (26.01 to 36.51) | -0.13 (-2.27 to 2.01) | 4.73 (-2.04 to 11.50) | 4.86 (-1.91 to 11.63) |
| Third quartile | Prevalence | 5.46 (5.00 to 5.91) | 7.56 (6.57 to 8.55) | 9.72 (8.54 to 10.91) | 10.02 (8.87 to 11.17) | 11.45 (10.44 to 12.46) | 13.37 (12.04 to 14.70) | **1.40 (1.11 to 1.69)** | **1.92 (0.24 to 3.59)** | 0.52 (-1.16 to 2.20) |
|  | Awareness | 81.39 (77.94 to 84.84) | 67.43 (60.88 to 73.98) | 61.59 (55.26 to 67.93) | 59.17 (53.47 to 64.88) | 62.53 (58.33 to 66.74) | 71.18 (66.79 to 75.58) | **-3.26 (-4.92 to -1.60)** | **8.65 (2.56 to 14.75)** | **11.91 (5.82 to 18.01)** |
|  | Treatment | 47.79 (43.30 to 52.29) | 53.52 (46.78 to 60.26) | 52.53 (45.91 to 59.15) | 52.31 (46.55 to 58.08) | 59.05 (54.71 to 63.40) | 65.67 (60.80 to 70.53) | **2.15 (0.42 to 3.89)** | **6.61 (0.08 to 13.14)** | 4.46 (-2.07 to 10.99) |
|  | Control among participants with diabetes | 27.95 (21.18 to 34.73) | 26.05 (19.66 to 32.45) | 22.26 (16.59 to 27.92) | 26.09 (20.84 to 31.35) | 25.70 (21.98 to 29.42) | 28.59 (24.26 to 32.92) | 0.21 (-1.73 to 2.14) | 2.89 (-2.82 to 8.60) | 2.68 (-3.35 to 8.71) |
|  | Control among participants being treated | 29.40 (18.30 to 40.50) | 20.48 (13.25 to 27.70) | 19.25 (12.81 to 25.69) | 29.53 (22.15 to 36.92) | 24.00 (19.40 to 28.61) | 31.05 (25.41 to 36.70) | 1.41 (-1.00 to 3.81) | 7.05 (-0.26 to 14.36) | 5.64 (-1.67 to 12.95) |
| Highest quartile | Prevalence | 4.98 (4.53 to 5.42) | 7.16 (6.18 to 8.15) | 8.76 (7.64 to 9.88) | 9.17 (8.15 to 10.18) | 9.60 (8.80 to 10.40) | 11.35 (10.20 to 12.49) | **1.05 (0.80 to 1.30)** | **1.74 (0.36 to 3.13)** | 0.69 (-0.70 to 2.08) |
|  | Awareness | 80.42 (76.71 to 84.12) | 70.36 (64.55 to 76.17) | 61.69 (55.44 to 67.95) | 56.97 (51.06 to 62.88) | 63.34 (58.77 to 67.90) | 69.61 (65.25 to 73.97) | **-3.59 (-5.25 to -1.92)** | 6.27 (-0.03 to 12.58) | **9.86 (3.56 to 16.17)** |
|  | Treatment | 42.98 (38.49 to 47.47) | 53.47 (47.15 to 59.79) | 54.08 (47.84 to 60.31) | 50.09 (44.09 to 56.10) | 57.78 (53.12 to 62.45) | 66.46 (62.04 to 70.88) | **2.13 (0.37 to 3.89)** | **8.68 (2.28 to 15.07)** | **6.55 (0.15 to 12.95)** |
|  | Control among participants with diabetes | 31.63 (23.89 to 39.37) | 27.65 (20.92 to 34.38) | 22.30 (16.90 to 27.70) | 23.73 (18.47 to 28.99) | 26.86 (22.86 to 30.86) | 27.94 (23.00 to 32.88) | -0.18 (-2.20 to 1.84) | 1.08 (-5.28 to 7.45) | 1.26 (-5.42 to 7.94) |
|  | Control among participants being treated | 17.72 (7.99 to 27.45) | 21.11 (12.93 to 29.29) | 24.06 (16.69 to 31.43) | 19.31 (12.88 to 25.74) | 22.48 (17.64 to 27.32) | 27.63 (21.59 to 33.66) | 0.16 (-2.42 to 2.74) | 5.15 (-2.61 to 12.90) | 4.99 (-2.77 to 12.75) |
| Smoking status | | | | | | | | | | |
| Smoker | Prevalence | 10.32 (9.42 to 11.23) | 10.85 (9.95 to 11.75) | 12.81 (11.79 to 13.83) | 14.71 (13.59 to 15.83) | 15.84 (14.90 to 16.77) | 18.09 (16.90 to 19.28) | **1.60 (1.26 to 1.95)** | **2.26 (0.74 to 3.77)** | 0.66 (-0.86 to 2.18) |
|  | Awareness | 63.61 (59.40 to 67.82) | 70.38 (66.38 to 74.38) | 66.57 (62.73 to 70.41) | 61.71 (57.89 to 65.53) | 64.01 (61.17 to 66.86) | 72.09 (68.96 to 75.22) | **-1.68 (-2.96 to -0.39)** | **8.08 (3.86 to 12.29)** | **9.76 (5.55 to 13.98)** |
|  | Treatment | 38.45 (34.05 to 42.85) | 53.99 (49.71 to 58.27) | 57.80 (54.11 to 61.48) | 54.19 (50.23 to 58.16) | 58.65 (55.68 to 61.62) | 66.95 (63.52 to 70.37) | **2.11 (0.76 to 3.45)** | **8.30 (3.78 to 12.82)** | **6.19 (1.67 to 10.71)** |
|  | Control among participants with diabetes | 33.75 (28.66 to 38.84) | 29.47 (25.51 to 33.44) | 25.82 (21.89 to 29.75) | 23.51 (20.19 to 26.83) | 28.68 (26.03 to 31.32) | 29.22 (26.00 to 32.45) | -0.50 (-1.79 to 0.80) | 0.55 (-3.63 to 4.72) | 1.04 (-3.33 to 5.42) |
|  | Control among participants being treated | 25.53 (18.10 to 32.97) | 26.60 (21.59 to 31.60) | 23.68 (19.49 to 27.87) | 22.88 (18.63 to 27.12) | 26.64 (23.28 to 30.01) | 30.02 (26.06 to 33.98) | 0.18 (-1.50 to 1.86) | 3.38 (-1.83 to 8.58) | 3.20 (-2.01 to 8.41) |
| Non-smoker | Prevalence | 7.88 (7.21 to 8.55) | 9.27 (8.51 to 10.02) | 10.60 (9.78 to 11.41) | 12.27 (11.44 to 13.09) | 12.66 (11.92 to 13.41) | 13.62 (12.69 to 14.55) | **1.21 (0.93 to 1.48)** | 0.96 (-0.26 to 2.17) | -0.25 (-1.47 to 0.97) |
|  | Awareness | 64.13 (59.82 to 68.45) | 77.48 (73.95 to 81.01) | 67.05 (63.33 to 70.77) | 66.85 (63.69 to 70.02) | 69.26 (66.55 to 71.97) | 73.05 (70.24 to 75.86) | **-1.30 (-2.49 to -0.11)** | 3.79 (-0.11 to 7.69) | **5.09 (1.19 to 8.99)** |
|  | Treatment | 48.87 (44.40 to 53.34) | 63.04 (59.29 to 66.80) | 59.52 (55.69 to 63.35) | 61.41 (58.18 to 64.65) | 64.71 (61.86 to 67.55) | 69.80 (66.86 to 72.75) | **1.67 (0.43 to 2.92)** | **5.10 (1.01 to 9.18)** | 3.43 (-0.66 to 7.52) |
|  | Control among participants with diabetes | 26.25 (21.83 to 30.67) | 27.76 (23.71 to 31.81) | 24.22 (20.63 to 27.81) | 21.53 (18.50 to 24.57) | 26.06 (23.59 to 28.53) | 29.06 (25.86 to 32.26) | -0.47 (-1.71 to 0.76) | 3.00 (-1.07 to 7.06) | 3.47 (-0.78 to 7.71) |
|  | Control among participants being treated | 23.81 (17.76 to 29.86) | 22.59 (18.16 to 27.01) | 26.10 (21.31 to 30.88) | 21.59 (17.66 to 25.52) | 26.09 (23.15 to 29.03) | 31.36 (27.52 to 35.20) | 0.64 (-0.85 to 2.12) | **5.27 (0.42 to 10.12)** | 4.63 (-0.22 to 9.48) |

Abbreviations: BMI, body mass index (calculated as weight in kilograms divided by height in meters squared); CI, confidence interval; KNHANES, Korea National Health and Nutrition Examination Survey.

Numbers in bold indicate a significant difference (P < 0.05).

^a^ All βs and β_diff_s were expressed by multiplying 100.

^b^ BMI was divided into four groups according to Asian-Pacific guidelines: underweight (<18.5 kg/m^2^), normal (18.5–22.9 kg/m^2^), overweight (23.0–24.9 kg/m^2^), and obese (≥25 kg/m^2^).
