# Supplementary material for: Prevalence, Awareness, Treatment, and Control of Type 2 Diabetes in South Korea (1998 to 2022): Nationwide Cross-Sectional Study
Source: JMIR Public Health Surveill. 2024 Aug 27;10:e59571. doi: 10.2196/59571 (PMC11387923; doi:10.2196/59571)
Supplement: Multimedia Appendix 2 [file publichealth_v10i1e59571_app2.docx]

Contents of Multimedia Appendix 2.

| **Supplementary Material** | Supplementary Material |
| --- | --- |
| **Table S1** | National trends of the prevalence, awareness, treatment, control among participants with diabetes, and control among participants being treated by age among males and females before and during the COVID-19 pandemic (weighted % [95% CI]) based on data obtained from the KNHANES. |
| **Table S2** | National trends of the prevalence, awareness, treatment, control among participants with diabetes, and control among participants being treated by region of residence among males and females before and during the COVID-19 pandemic (weighted % [95% CI]) based on data obtained from the KNHANES. |
| **Table S3** | National trends of the prevalence, awareness, treatment, e participants with diabetes, and control among participants being treated by BMI group among males and females before and during the COVID-19 pandemic (weighted % [95% CI]) based on data obtained from the KNHANES. |
| **Table S4** | National trends of the prevalence, awareness, treatment, control among participants with diabetes, and control among participants being treated by educational background among males and females before and during the COVID-19 pandemic (weighted % [95% CI]) based on data obtained from the KNHANES. |
| **Table S5** | National trends of the prevalence, awareness, treatment, control among participants with diabetes, and control among participants being treated by household income among males and females before and during the COVID-19 pandemic (weighted % [95% CI]) based on data obtained from the KNHANES. |
| **Table S6** | National trends of the prevalence, awareness, treatment, control among participants with diabetes, and control among participants being treated by smoking status among males and females before and during the COVID-19 pandemic (weighted % [95% CI]) based on data obtained from the KNHANES. |
| **Table S7** | Weighted odds ratios of prevalence, awareness, treatment, control among participants with diabetes, and control among participants being treated by age among males and females based on data obtained from the KNHANES. |
| **Table S8** | Weighted odds ratios of prevalence, awareness, treatment, control among participants with diabetes, and control among participants being treated by region of residence among males and females based on data obtained from the KNHANES. |
| **Table S9** | Weighted odds ratios of prevalence, awareness, treatment, control among participants with diabetes, and control among participants being treated by BMI group among males and females based on data obtained from the KNHANES. |
| **Table S10** | Weighted odds ratios of prevalence, awareness, treatment, control among participants with diabetes, and control among participants being treated by educational background among males and females based on data obtained from the KNHANES. |
| **Table S11** | Weighted odds ratios of prevalence, awareness, treatment, control among participants with diabetes, and control among participants being treated by household income among males and females based on data obtained from the KNHANES. |
| **Table S12** | Weighted odds ratios of prevalence, awareness, treatment, control among participants with diabetes, and control among participants being treated by smoking status among males and females based on data obtained from the KNHANES. |

**Table S1.** National trends of the prevalence, awareness, treatment, control among participants with diabetes, and control among participants being treated by age among males and females before and during the COVID-19 pandemic (weighted % [95% CI]) based on data obtained from the KNHANES.

| Sex | Rate | Age, years | Before the pandemic | | | | | During the pandemic | Trends in the pre-pandemic,  β (95% CI) ^a^ | Trends in the pandemic,  β (95% CI) ^a^ | Trend differences, βdiff (95% CI) ^a^ |
| --- | --- | --- | --- | --- | --- | --- | --- | --- | --- | --- | --- |
|  |  |  | 1998-2005 | 2007-2009 | 2010-2012 | 2013-2015 | 2016-2019 | 2020-2022 |  |  |  |
| Male | Prevalence | 30 to 39 | 1.84 (1.46 to 2.22) | 3.23 (2.13 to 4.33) | 3.54 (2.39 to 4.69) | 2.87 (1.79 to 3.96) | 3.52 (2.48 to 4.56) | 4.46 (2.96 to 5.97) | **0.29 (0.01 to 0.57)** | 0.95 (-0.88 to 2.78) | 0.66 (-1.19 to 2.52) |
|  |  | 40 to 49 | 5.65 (5.03 to 6.27) | 8.24 (6.60 to 9.87) | 8.33 (6.49 to 10.16) | 11.45 (9.44 to 13.46) | 10.97 (9.26 to 12.68) | 11.60 (9.55 to 13.65) | **1.35 (0.88 to 1.82)** | 0.63 (-2.05 to 3.31) | -0.72 (-3.44 to 2.00) |
|  |  | 50 to 59 | 11.10 (9.99 to 12.20) | 15.99 (13.75 to 18.22) | 18.34 (15.98 to 20.71) | 17.14 (14.82 to 19.46) | 19.40 (17.46 to 21.33) | 23.49 (20.74 to 26.23) | **1.57 (0.96 to 2.18)** | **4.09 (0.74 to 7.44)** | 2.52 (-0.89 to 5.92) |
|  |  | 60 to 69 | 15.36 (13.92 to 16.80) | 23.80 (20.92 to 26.69) | 24.15 (21.70 to 26.60) | 30.68 (27.62 to 33.74) | 26.69 (24.31 to 29.06) | 27.29 (24.51 to 30.06) | **2.58 (1.83 to 3.34)** | 0.60 (-3.05 to 4.25) | -1.98 (-5.71 to 1.74) |
|  |  | ≥70 | 13.82 (11.93 to 15.70) | 16.72 (13.91 to 19.53) | 25.04 (22.20 to 27.89) | 25.58 (22.47 to 28.69) | 29.30 (26.74 to 31.87) | 29.94 (27.12 to 32.76) | **3.86 (3.03 to 4.70)** | 0.64 (-3.17 to 4.45) | -3.22 (-7.12 to 0.67) |
|  | Awareness | 30 to 39 | 72.68 (63.91 to 81.45) | 30.39 (15.83 to 44.95) | 30.22 (15.19 to 45.25) | 41.83 (23.67 to 59.98) | 25.55 (14.28 to 36.81) | 56.26 (39.01 to 73.51) | **-6.05 (-10.29 to -1.80)** | **30.72 (10.13 to 51.31)** | **36.76 (15.74 to 57.78)** |
|  |  | 40 to 49 | 77.45 (72.81 to 82.08) | 56.61 (47.15 to 66.06) | 42.79 (32.47 to 53.10) | 43.79 (34.27 to 53.30) | 42.76 (35.10 to 50.42) | 60.22 (51.10 to 69.33) | **-6.84 (-9.45 to -4.22)** | **17.46 (5.57 to 29.35)** | **24.29 (12.12 to 36.46)** |
|  |  | 50 to 59 | 86.30 (82.49 to 90.10) | 76.68 (69.64 to 83.71) | 69.34 (62.54 to 76.14) | 57.43 (50.01 to 64.84) | 62.44 (57.09 to 67.79) | 69.07 (63.38 to 74.76) | **-5.87 (-7.87 to -3.87)** | 6.63 (-1.18 to 14.45) | **12.50 (4.43 to 20.57)** |
|  |  | 60 to 69 | 87.14 (83.75 to 90.54) | 88.30 (84.15 to 92.45) | 77.84 (72.48 to 83.19) | 72.20 (66.69 to 77.71) | 73.84 (69.36 to 78.31) | 74.13 (69.09 to 79.17) | **-4.27 (-5.76 to -2.78)** | 0.29 (-6.44 to 7.02) | 4.56 (-2.33 to 11.45) |
|  |  | ≥70 | 88.81 (84.01 to 93.61) | 75.51 (67.83 to 83.19) | 73.38 (66.56 to 80.20) | 79.47 (73.10 to 85.83) | 77.71 (73.62 to 81.80) | 81.94 (77.38 to 86.49) | -0.43 (-2.28 to 1.42) | 4.23 (-1.90 to 10.35) | 4.66 (-1.74 to 11.06) |
|  | Treatment | 30 to 39 | 28.03 (18.44 to 37.63) | 19.69 (7.08 to 32.31) | 28.80 (13.86 to 43.74) | 33.90 (16.70 to 51.11) | 23.64 (12.46 to 34.83) | 46.33 (29.29 to 63.37) | 0.75 (-3.15 to 4.65) | **22.69 (2.31 to 43.06)** | **21.93 (1.19 to 42.68)** |
|  |  | 40 to 49 | 33.41 (27.94 to 38.89) | 38.20 (29.37 to 47.03) | 32.75 (23.64 to 41.87) | 32.28 (23.27 to 41.29) | 38.67 (31.03 to 46.31) | 55.70 (46.78 to 64.62) | 0.46 (-2.10 to 3.02) | **17.03 (5.31 to 28.75)** | **16.57 (4.58 to 28.57)** |
|  |  | 50 to 59 | 45.36 (40.08 to 50.65) | 56.41 (48.68 to 64.15) | 60.32 (53.39 to 67.25) | 52.39 (44.93 to 59.85) | 54.68 (49.10 to 60.26) | 64.35 (57.93 to 70.77) | 0.30 (-1.84 to 2.44) | **9.67 (1.15 to 18.19)** | **9.37 (0.58 to 18.16)** |
|  |  | 60 to 69 | 46.24 (41.14 to 51.34) | 73.30 (67.22 to 79.38) | 67.97 (61.77 to 74.18) | 66.22 (60.43 to 72.00) | 69.33 (64.75 to 73.92) | 68.12 (62.69 to 73.54) | **2.13 (0.36 to 3.90)** | -1.22 (-8.31 to 5.87) | -3.35 (-10.65 to 3.96) |
|  |  | ≥70 | 54.96 (47.50 to 62.43) | 63.41 (55.28 to 71.54) | 65.93 (59.06 to 72.81) | 75.02 (68.55 to 81.48) | 73.26 (68.83 to 77.68) | 78.39 (73.51 to 83.28) | **4.19 (2.14 to 6.24)** | 5.14 (-1.45 to 11.73) | 0.95 (-5.95 to 7.85) |
|  | Control among participants with diabetes | 30 to 39 | 48.94 (33.25 to 64.63) | 32.15 (15.39 to 48.92) | 13.46 (2.84 to 24.08) | 25.98 (9.29 to 42.67) | 26.52 (13.50 to 39.55) | 18.14 (4.25 to 32.03) | -1.91 (-7.62 to 3.80) | -8.38 (-27.45 to 10.68) | -6.47 (-26.38 to 13.43) |
|  |  | 40 to 49 | 33.10 (24.13 to 42.07) | 29.69 (21.67 to 37.70) | 26.14 (16.23 to 36.06) | 25.34 (16.53 to 34.15) | 22.88 (16.58 to 29.19) | 22.64 (14.94 to 30.35) | -2.23 (-5.00 to 0.54) | -0.24 (-10.21 to 9.73) | 1.99 (-8.36 to 12.34) |
|  |  | 50 to 59 | 32.03 (23.34 to 40.73) | 27.54 (19.96 to 35.12) | 28.07 (21.09 to 35.06) | 22.52 (16.09 to 28.95) | 28.44 (23.54 to 33.33) | 28.68 (22.57 to 34.79) | -0.32 (-2.77 to 2.13) | 0.25 (-7.59 to 8.09) | 0.56 (-7.65 to 8.78) |
|  |  | 60 to 69 | 24.94 (16.39 to 33.48) | 32.51 (25.71 to 39.32) | 23.02 (17.18 to 28.87) | 21.34 (16.70 to 25.98) | 28.11 (23.44 to 32.77) | 32.57 (26.98 to 38.16) | -0.75 (-2.97 to 1.46) | 4.46 (-2.78 to 11.71) | 5.22 (-2.36 to 12.79) |
|  |  | ≥70 | 39.31 (25.18 to 53.43) | 36.01 (26.67 to 45.35) | 29.57 (21.87 to 37.27) | 29.21 (22.58 to 35.84) | 32.57 (27.68 to 37.46) | 34.55 (28.93 to 40.16) | -0.62 (-3.37 to 2.13) | 1.98 (-5.49 to 9.45) | 2.60 (-5.36 to 10.56) |
|  | Control among participants being treated | 30 to 39 | 56.74 (9.05 to 100.00) | 31.94 (0.00 to 65.52) | 8.30 (0.00 to 24.06) | 9.35 (0.00 to 26.83) | 27.87 (1.90 to 53.84) | N/A | -2.00 (-13.60 to 9.60) | **-27.87 (-53.86 to -1.88)** | -25.87 (-54.33 to 2.59) |
|  |  | 40 to 49 | 7.53 (0.00 to 17.77) | 27.40 (12.51 to 42.29) | 22.42 (7.51 to 37.33) | 24.57 (9.78 to 39.35) | 16.55 (8.18 to 24.92) | 26.67 (15.09 to 38.24) | -2.24 (-7.00 to 2.52) | 10.11 (-4.20 to 24.43) | 12.35 (-2.73 to 27.43) |
|  |  | 50 to 59 | 27.01 (14.29 to 39.74) | 18.02 (9.58 to 26.46) | 23.39 (15.14 to 31.65) | 19.79 (10.60 to 28.98) | 22.23 (15.89 to 28.57) | 26.30 (19.04 to 33.55) | 0.49 (-2.55 to 3.53) | 4.07 (-5.57 to 13.71) | 3.58 (-6.53 to 13.69) |
|  |  | 60 to 69 | 21.34 (10.19 to 32.49) | 31.40 (23.77 to 39.02) | 21.59 (14.79 to 28.39) | 21.13 (15.45 to 26.81) | 27.50 (21.73 to 33.28) | 34.32 (27.87 to 40.77) | -0.56 (-3.26 to 2.15) | 6.82 (-1.85 to 15.49) | 7.37 (-1.71 to 16.46) |
|  |  | ≥70 | 48.76 (27.20 to 70.32) | 26.81 (16.91 to 36.70) | 28.41 (20.50 to 36.32) | 31.30 (23.46 to 39.15) | 33.81 (28.20 to 39.42) | 36.81 (30.31 to 43.31) | 1.68 (-1.48 to 4.84) | 2.99 (-5.61 to 11.60) | 1.31 (-7.85 to 10.48) |
| Female | Prevalence | 30 to 39 | 0.86 (0.61 to 1.12) | 1.86 (1.25 to 2.47) | 2.20 (1.43 to 2.97) | 2.31 (1.47 to 3.15) | 2.03 (1.35 to 2.72) | 2.64 (1.60 to 3.69) | **0.28 (0.09 to 0.46)** | 0.61 (-0.64 to 1.86) | 0.33 (-0.93 to 1.60) |
|  |  | 40 to 49 | 2.77 (2.33 to 3.21) | 4.81 (3.72 to 5.90) | 5.54 (4.22 to 6.86) | 5.34 (4.18 to 6.51) | 5.06 (4.02 to 6.10) | 4.70 (3.50 to 5.89) | **0.46 (0.17 to 0.76)** | -0.36 (-1.95 to 1.23) | -0.83 (-2.44 to 0.79) |
|  |  | 50 to 59 | 8.41 (7.47 to 9.35) | 9.49 (7.89 to 11.10) | 10.40 (8.77 to 12.02) | 10.03 (8.45 to 11.61) | 10.97 (9.57 to 12.37) | 11.94 (10.03 to 13.85) | **0.53 (0.09 to 0.98)** | 0.97 (-1.40 to 3.34) | 0.44 (-1.97 to 2.85) |
|  |  | 60 to 69 | 14.90 (13.54 to 16.26) | 19.52 (17.19 to 21.86) | 20.13 (17.82 to 22.43) | 22.02 (19.50 to 24.55) | 20.04 (18.14 to 21.93) | 20.17 (18.00 to 22.33) | **1.09 (0.49 to 1.70)** | 0.13 (-2.74 to 3.00) | -0.96 (-3.90 to 1.97) |
|  |  | ≥70 | 15.12 (13.66 to 16.58) | 21.48 (18.80 to 24.16) | 24.84 (22.24 to 27.44) | 30.24 (27.24 to 33.25) | 30.24 (28.09 to 32.38) | 30.36 (27.90 to 32.82) | **3.68 (2.97 to 4.39)** | 0.13 (-3.14 to 3.40) | **-3.55 (-6.90 to -0.21)** |
|  | Awareness | 30 to 39 | 43.81 (29.05 to 58.57) | 68.01 (53.56 to 82.46) | 48.49 (30.34 to 66.63) | 36.92 (19.23 to 54.61) | 45.29 (27.95 to 62.62) | 53.94 (33.78 to 74.10) | -4.87 (-10.56 to 0.83) | 8.66 (-17.98 to 35.29) | 13.52 (-13.71 to 40.76) |
|  |  | 40 to 49 | 72.30 (64.90 to 79.70) | 62.33 (50.82 to 73.84) | 53.05 (40.92 to 65.18) | 50.18 (38.81 to 61.56) | 52.37 (42.09 to 62.66) | 50.94 (38.23 to 63.65) | **-4.51 (-8.00 to -1.03)** | -1.44 (-17.85 to 14.98) | 3.08 (-13.70 to 19.86) |
|  |  | 50 to 59 | 88.19 (84.40 to 91.98) | 69.43 (60.78 to 78.09) | 63.59 (55.38 to 71.79) | 57.23 (49.17 to 65.30) | 63.40 (56.70 to 70.09) | 64.66 (56.77 to 72.55) | **-4.79 (-7.07 to -2.51)** | 1.26 (-9.12 to 11.65) | 6.05 (-4.57 to 16.68) |
|  |  | 60 to 69 | 86.13 (82.69 to 89.57) | 83.92 (78.45 to 89.39) | 74.66 (68.91 to 80.40) | 69.47 (63.50 to 75.43) | 72.07 (67.42 to 76.72) | 78.23 (73.69 to 82.77) | **-4.04 (-5.59 to -2.49)** | 6.16 (-0.32 to 12.64) | **10.19 (3.53 to 16.86)** |
|  |  | ≥70 | 87.76 (84.20 to 91.32) | 83.67 (78.39 to 88.95) | 80.14 (75.28 to 85.00) | 79.04 (74.49 to 83.58) | 80.59 (77.16 to 84.02) | 84.65 (81.12 to 88.18) | **-1.38 (-2.76 to -0.01)** | 4.06 (-0.87 to 8.98) | **5.44 (0.33 to 10.55)** |
|  | Treatment | 30 to 39 | 16.07 (5.54 to 26.60) | 28.56 (12.55 to 44.57) | 25.27 (8.66 to 41.89) | 24.03 (8.60 to 39.47) | 33.52 (16.74 to 50.31) | 50.03 (29.63 to 70.43) | 2.34 (-2.99 to 7.67) | 16.51 (-9.96 to 42.97) | 14.17 (-12.83 to 41.16) |
|  |  | 40 to 49 | 39.94 (32.03 to 47.85) | 47.97 (36.24 to 59.71) | 45.87 (33.72 to 58.02) | 42.89 (31.67 to 54.12) | 43.10 (32.32 to 53.88) | 45.31 (32.30 to 58.31) | -0.52 (-4.11 to 3.07) | 2.20 (-14.75 to 19.15) | 2.72 (-14.60 to 20.05) |
|  |  | 50 to 59 | 52.38 (46.50 to 58.26) | 58.30 (49.12 to 67.48) | 56.68 (48.31 to 65.06) | 50.39 (42.31 to 58.47) | 58.06 (51.21 to 64.91) | 62.66 (54.77 to 70.54) | 0.23 (-2.19 to 2.66) | 4.60 (-5.87 to 15.06) | 4.37 (-6.38 to 15.11) |
|  |  | 60 to 69 | 51.95 (47.09 to 56.80) | 71.18 (64.92 to 77.43) | 70.03 (63.83 to 76.24) | 64.40 (58.27 to 70.53) | 69.41 (64.66 to 74.17) | 75.61 (70.94 to 80.29) | **1.89 (0.18 to 3.59)** | 6.20 (-0.48 to 12.87) | 4.31 (-2.58 to 11.20) |
|  |  | ≥70 | 59.95 (54.67 to 65.23) | 71.10 (64.74 to 77.45) | 71.56 (66.37 to 76.76) | 73.46 (68.72 to 78.20) | 76.87 (73.04 to 80.69) | 81.27 (77.45 to 85.09) | **3.02 (1.41 to 4.64)** | 4.41 (-0.99 to 9.80) | 1.38 (-4.25 to 7.01) |
|  | Control among participants with diabetes | 30 to 39 | 53.07 (35.62 to 70.53) | 25.61 (10.02 to 41.21) | 30.52 (12.90 to 48.13) | 17.44 (3.80 to 31.08) | 39.45 (22.05 to 56.85) | 39.85 (20.19 to 59.52) | -0.48 (-6.64 to 5.67) | 0.40 (-25.87 to 26.68) | 0.89 (-26.10 to 27.87) |
|  |  | 40 to 49 | 26.93 (15.56 to 38.30) | 19.65 (9.00 to 30.29) | 17.59 (9.03 to 26.14) | 20.11 (10.76 to 29.46) | 22.03 (13.54 to 30.51) | 22.27 (11.92 to 32.62) | 0.34 (-3.22 to 3.89) | 0.24 (-13.19 to 13.67) | -0.09 (-13.99 to 13.80) |
|  |  | 50 to 59 | 17.13 (9.01 to 25.26) | 22.13 (14.01 to 30.26) | 19.99 (12.55 to 27.44) | 23.72 (16.23 to 31.20) | 26.30 (20.50 to 32.10) | 29.57 (21.65 to 37.48) | 1.88 (-0.77 to 4.53) | 3.27 (-6.55 to 13.09) | 1.39 (-8.78 to 11.56) |
|  |  | 60 to 69 | 24.31 (17.02 to 31.60) | 28.09 (21.31 to 34.88) | 20.57 (14.44 to 26.70) | 18.68 (13.33 to 24.02) | 24.41 (20.01 to 28.80) | 23.89 (19.32 to 28.46) | -0.85 (-2.91 to 1.22) | -0.51 (-6.85 to 5.82) | 0.33 (-6.33 to 7.00) |
|  |  | ≥70 | 31.20 (23.36 to 39.04) | 29.96 (23.32 to 36.60) | 33.04 (27.09 to 38.99) | 20.74 (16.07 to 25.40) | 28.08 (23.99 to 32.17) | 32.27 (27.38 to 37.17) | -1.44 (-3.49 to 0.60) | 4.20 (-2.20 to 10.60) | 5.64 (-1.08 to 12.36) |
|  | Control among participants being treated | 30 to 39 | 0.04 (0.00 to 0.11) | 12.43 (0.00 to 29.58) | 13.05 (0.00 to 37.27) | 19.88 (0.00 to 54.02) | 43.40 (11.00 to 75.81) | 49.14 (18.62 to 79.67) | 10.05 (-1.18 to 21.28) | 5.74 (-38.80 to 50.28) | -4.31 (-50.24 to 41.63) |
|  |  | 40 to 49 | 12.25 (0.00 to 34.41) | 13.65 (1.28 to 26.01) | 16.12 (3.45 to 28.78) | 12.91 (1.50 to 24.32) | 5.94 (0.00 to 12.43) | 22.55 (5.53 to 39.57) | -2.41 (-6.57 to 1.74) | 16.61 (-1.62 to 34.85) | **19.02 (0.32 to 37.73)** |
|  |  | 50 to 59 | 17.42 (6.99 to 27.84) | 19.75 (9.25 to 30.25) | 23.02 (11.76 to 34.27) | 22.92 (12.52 to 33.32) | 25.77 (18.50 to 33.05) | 32.62 (21.77 to 43.46) | 1.85 (-1.61 to 5.32) | 6.84 (-6.22 to 19.90) | 4.99 (-8.53 to 18.50) |
|  |  | 60 to 69 | 28.62 (18.23 to 39.00) | 24.44 (16.94 to 31.94) | 23.22 (15.02 to 31.42) | 22.45 (15.11 to 29.80) | 23.87 (18.70 to 29.05) | 25.67 (20.01 to 31.32) | -0.44 (-2.93 to 2.05) | 1.79 (-5.90 to 9.49) | 2.23 (-5.86 to 10.32) |
|  |  | ≥70 | 24.80 (13.84 to 35.76) | 27.70 (20.42 to 34.97) | 36.06 (28.56 to 43.56) | 21.19 (15.56 to 26.82) | 30.77 (26.06 to 35.49) | 34.66 (29.18 to 40.15) | -0.08 (-2.51 to 2.35) | 3.89 (-3.36 to 11.14) | 3.97 (-3.68 to 11.62) |

Abbreviations: CI, confidence interval; KNHANES, Korea National Health and Nutrition Examination Survey.

Numbers in bold indicate a significant difference (P < 0.05).

^a^ All βs and β_diff_s were expressed by multiplying 100.

**Table S2.** National trends of the prevalence, awareness, treatment, control among participants with diabetes, and control among participants being treated by region of residence among males and females before and during the COVID-19 pandemic (weighted % [95% CI]) based on data obtained from the KNHANES.

| Sex | Rate | Region of residence | Before the pandemic | | | | | During the pandemic | Trends in the pre-pandemic,  β (95% CI) ^a^ | Trends in the pandemic,  β (95% CI) ^a^ | Trend differences, βdiff (95% CI) ^a^ |
| --- | --- | --- | --- | --- | --- | --- | --- | --- | --- | --- | --- |
|  |  |  | 1998-2005 | 2007-2009 | 2010-2012 | 2013-2015 | 2016-2019 | 2020-2022 |  |  |  |
| Male | Prevalence | Urban | 6.82 (6.38 to 7.26) | 10.90 (9.86 to 11.94) | 12.29 (11.24 to 13.34) | 14.80 (13.60 to 16.00) | 14.72 (13.77 to 15.68) | 17.66 (16.32 to 19.01) | **1.87 (1.58 to 2.15)** | 2.94 (1.29 to 4.59) | 1.07 (-0.60 to 2.75) |
|  |  | Rural | 8.63 (7.75 to 9.52) | 11.41 (9.76 to 13.07) | 15.00 (12.54 to 17.47) | 15.54 (12.90 to 18.18) | 21.79 (19.35 to 24.24) | 20.06 (17.73 to 22.39) | **3.04 (2.41 to 3.68)** | -1.73 (-5.12 to 1.65) | **-4.77 (-8.22 to -1.33)** |
|  | Awareness | Urban | 84.40 (81.83 to 86.96) | 71.88 (67.21 to 76.56) | 63.31 (58.65 to 67.98) | 62.34 (58.20 to 66.49) | 62.61 (59.42 to 65.81) | 70.78 (67.21 to 74.34) | **-4.19 (-5.42 to -2.97)** | **8.17 (3.39 to 12.95)** | **12.36 (7.43 to 17.30)** |
|  |  | Rural | 81.10 (76.41 to 85.78) | 69.15 (61.66 to 76.64) | 67.49 (59.21 to 75.78) | 61.41 (52.39 to 70.43) | 65.77 (59.96 to 71.59) | 72.11 (66.04 to 78.17) | **-2.89 (-5.03 to -0.75)** | 6.33 (-2.10 to 14.76) | **9.22 (0.53 to 17.92)** |
|  | Treatment | Urban | 42.00 (38.77 to 45.23) | 55.25 (50.32 to 60.18) | 53.94 (49.50 to 58.39) | 55.68 (51.44 to 59.93) | 57.55 (54.28 to 60.82) | 65.57 (61.53 to 69.60) | **2.34 (1.05 to 3.63)** | **8.02 (2.83 to 13.21)** | **5.68 (0.34 to 11.02)** |
|  |  | Rural | 44.35 (38.79 to 49.91) | 52.61 (44.61 to 60.62) | 61.32 (53.57 to 69.07) | 54.80 (45.73 to 63.87) | 59.83 (53.29 to 66.36) | 67.50 (61.72 to 73.28) | **2.59 (0.25 to 4.93)** | 7.67 (-1.06 to 16.40) | 5.08 (-3.96 to 14.12) |
|  | Control among participants with diabetes | Urban | 32.56 (26.55 to 38.58) | 29.86 (25.45 to 34.28) | 25.70 (21.11 to 30.30) | 23.89 (20.45 to 27.33) | 27.73 (24.78 to 30.69) | 28.60 (25.00 to 32.20) | -0.76 (-2.22 to 0.70) | 0.87 (-3.80 to 5.53) | 1.62 (-3.27 to 6.52) |
|  |  | Rural | 33.52 (24.56 to 42.48) | 34.06 (25.58 to 42.54) | 25.35 (18.50 to 32.19) | 24.72 (17.36 to 32.07) | 29.34 (23.62 to 35.06) | 33.14 (27.41 to 38.86) | -1.07 (-3.70 to 1.55) | 3.80 (-4.33 to 11.92) | 4.87 (-3.66 to 13.41) |
|  | Control among participants being treated | Urban | 27.02 (18.15 to 35.89) | 24.11 (18.54 to 29.68) | 23.62 (18.58 to 28.67) | 23.33 (18.79 to 27.87) | 25.56 (22.02 to 29.11) | 29.55 (25.15 to 33.95) | 0.37 (-1.49 to 2.24) | 3.99 (-1.66 to 9.63) | 3.61 (-2.34 to 9.56) |
|  |  | Rural | 24.41 (11.12 to 37.70) | 32.71 (22.19 to 43.24) | 21.75 (14.97 to 28.54) | 22.30 (13.86 to 30.74) | 28.70 (21.55 to 35.84) | 32.80 (26.49 to 39.11) | -0.03 (-3.37 to 3.32) | 4.10 (-5.46 to 13.67) | 4.13 (-6.00 to 14.26) |
| Female | Prevalence | Urban | 5.87 (5.48 to 6.26) | 8.88 (7.98 to 9.78) | 9.81 (8.94 to 10.67) | 11.47 (10.55 to 12.40) | 11.63 (10.84 to 12.42) | 12.26 (11.23 to 13.29) | **1.34 (1.11 to 1.58)** | 0.64 (-0.67 to 1.94) | -0.71 (-2.03 to 0.62) |
|  |  | Rural | 7.79 (6.85 to 8.74) | 9.72 (8.25 to 11.19) | 12.82 (10.77 to 14.87) | 13.57 (11.35 to 15.79) | 16.10 (13.97 to 18.23) | 18.16 (15.73 to 20.60) | **2.05 (1.50 to 2.60)** | 2.06 (-1.20 to 5.33) | 0.02 (-3.30 to 3.33) |
|  | Awareness | Urban | 83.97 (81.35 to 86.58) | 75.10 (70.87 to 79.33) | 70.88 (66.72 to 75.05) | 68.37 (64.77 to 71.98) | 70.21 (67.18 to 73.25) | 74.54 (71.31 to 77.76) | **-2.70 (-3.83 to -1.58)** | 4.32 (-0.10 to 8.75) | **7.03 (2.46 to 11.59)** |
|  |  | Rural | 82.70 (77.70 to 87.70) | 82.46 (77.49 to 87.44) | 66.23 (59.51 to 72.95) | 61.41 (53.83 to 69.00) | 72.70 (67.53 to 77.88) | 74.84 (69.15 to 80.54) | **-3.39 (-5.21 to -1.58)** | 2.14 (-5.49 to 9.78) | 5.54 (-2.31 to 13.38) |
|  | Treatment | Urban | 51.21 (47.81 to 54.60) | 61.07 (56.41 to 65.72) | 62.37 (57.82 to 66.91) | 62.00 (58.27 to 65.73) | 65.72 (62.55 to 68.89) | 71.33 (67.94 to 74.72) | **2.47 (1.25 to 3.69)** | **5.61 (0.97 to 10.25)** | 3.14 (-1.66 to 7.94) |
|  |  | Rural | 51.60 (45.82 to 57.38) | 68.45 (61.76 to 75.15) | 60.72 (54.31 to 67.13) | 55.89 (48.46 to 63.32) | 67.73 (61.82 to 73.65) | 72.36 (66.66 to 78.05) | 1.62 (-0.40 to 3.64) | 4.62 (-3.51 to 12.76) | 3.00 (-5.38 to 11.38) |
|  | Control among participants with diabetes | Urban | 27.50 (22.36 to 32.63) | 25.89 (21.26 to 30.52) | 24.23 (19.99 to 28.47) | 21.54 (18.05 to 25.03) | 26.16 (23.33 to 28.98) | 28.39 (24.70 to 32.07) | -0.14 (-1.55 to 1.27) | 2.23 (-2.41 to 6.87) | 2.37 (-2.48 to 7.22) |
|  |  | Rural | 26.29 (19.25 to 33.33) | 26.30 (19.43 to 33.17) | 24.80 (17.97 to 31.64) | 16.59 (9.44 to 23.75) | 27.72 (22.12 to 33.32) | 30.00 (24.49 to 35.52) | -0.15 (-2.49 to 2.18) | 2.28 (-5.63 to 10.19) | 2.43 (-5.82 to 10.68) |
|  | Control among participants being treated | Urban | 25.73 (17.98 to 33.48) | 22.93 (17.80 to 28.05) | 26.54 (20.79 to 32.29) | 21.86 (17.29 to 26.42) | 26.21 (22.82 to 29.61) | 30.59 (26.01 to 35.17) | 0.47 (-1.25 to 2.20) | 4.37 (-1.33 to 10.08) | 3.90 (-2.06 to 9.86) |
|  |  | Rural | 17.91 (9.34 to 26.48) | 23.25 (15.40 to 31.10) | 27.55 (18.74 to 36.36) | 17.53 (8.87 to 26.19) | 27.32 (20.50 to 34.14) | 33.96 (26.95 to 40.97) | 0.81 (-2.10 to 3.72) | 6.64 (-3.17 to 16.45) | 5.83 (-4.41 to 16.06) |

Abbreviations: CI, confidence interval; KNHANES, Korea National Health and Nutrition Examination Survey.

Numbers in bold indicate a significant difference (P < 0.05).

^a^ All βs and β_diff_s were expressed by multiplying 100.

**Table S3.** National trends of the prevalence, awareness, treatment, control among participants with diabetes, and control among participants being treated by BMI group among males and females before and during the COVID-19 pandemic (weighted % [95% CI]) based on data obtained from the KNHANES.

| Sex | Rate | BMI group ^a^ | Before the pandemic | | | | | During the pandemic | Trends in the pre-pandemic,  β (95% CI) ^b^ | Trends in the pandemic,  β (95% CI) ^b^ | Trend differences, βdiff (95% CI) ^b^ |
| --- | --- | --- | --- | --- | --- | --- | --- | --- | --- | --- | --- |
|  |  |  | 1998-2005 | 2007-2009 | 2010-2012 | 2013-2015 | 2016-2019 | 2020-2022 |  |  |  |
| Male | Prevalence | Underweight | 10.91 (5.39 to 16.43) | 6.80 (2.89 to 10.71) | 9.20 (3.49 to 14.91) | 8.53 (3.32 to 13.74) | 7.32 (3.27 to 11.37) | 7.11 (0.03 to 14.20) | -0.12 (-1.68 to 1.45) | -0.21 (-8.38 to 7.96) | -0.09 (-8.41 to 8.23) |
|  |  | Normal | 8.74 (7.21 to 10.26) | 7.90 (6.59 to 9.21) | 11.46 (9.88 to 13.04) | 12.63 (10.90 to 14.37) | 12.93 (11.50 to 14.35) | 14.75 (12.80 to 16.70) | **1.51 (0.97 to 2.05)** | 1.82 (-0.59 to 4.23) | 0.31 (-2.16 to 2.78) |
|  |  | Overweight | 11.71 (9.86 to 13.56) | 10.14 (8.63 to 11.66) | 12.56 (10.72 to 14.41) | 13.79 (11.91 to 15.68) | 14.17 (12.62 to 15.72) | 15.60 (13.61 to 17.59) | **1.17 (0.56 to 1.77)** | 1.43 (-1.09 to 3.94) | 0.26 (-2.33 to 2.85) |
|  |  | Obese | 14.32 (12.54 to 16.10) | 14.51 (12.87 to 16.15) | 14.63 (12.94 to 16.32) | 17.89 (16.08 to 19.71) | 19.32 (17.83 to 20.82) | 21.20 (19.31 to 23.09) | **1.65 (1.04 to 2.26)** | 1.87 (-0.53 to 4.28) | 0.22 (-2.26 to 2.71) |
|  | Awareness | Underweight | 58.78 (30.76 to 86.79) | 75.37 (49.11 to 100.00) | 72.48 (40.74 to 100.00) | 71.80 (36.29 to 100.00) | 46.27 (17.98 to 74.56) | 94.42 (85.22 to 100.00) | -5.66 (-16.00 to 4.69) | **48.16 (18.40 to 77.91)** | **53.81 (22.31 to 85.32)** |
|  |  | Normal | 61.03 (52.77 to 69.30) | 72.48 (64.34 to 80.62) | 73.83 (66.89 to 80.78) | 69.67 (62.84 to 76.50) | 70.82 (65.32 to 76.33) | 81.51 (76.16 to 86.87) | -0.15 (-2.70 to 2.41) | **10.69 (3.00 to 18.38)** | **10.84 (2.73 to 18.94)** |
|  |  | Overweight | 48.32 (39.64 to 56.99) | 77.43 (70.34 to 84.52) | 65.45 (58.44 to 72.45) | 63.89 (56.93 to 70.86) | 69.71 (64.14 to 75.29) | 74.02 (67.83 to 80.21) | -0.27 (-2.73 to 2.19) | 4.31 (-4.04 to 12.66) | 4.58 (-4.13 to 13.28) |
|  |  | Obese | 53.34 (46.35 to 60.34) | 68.02 (62.24 to 73.80) | 56.56 (50.52 to 62.61) | 56.87 (51.14 to 62.60) | 57.53 (53.66 to 61.40) | 65.07 (60.69 to 69.44) | **-2.05 (-3.95 to -0.15)** | **7.54 (1.71 to 13.37)** | **9.59 (3.46 to 15.73)** |
|  | Treatment | Underweight | 28.78 (8.53 to 49.03) | 56.77 (27.99 to 85.55) | 43.15 (11.77 to 74.53) | 57.98 (24.11 to 91.86) | 38.23 (10.61 to 65.85) | 89.60 (74.79 to 100.00) | -0.89 (-11.06 to 9.27) | **51.37 (20.05 to 82.70)** | **52.27 (19.33 to 85.20)** |
|  |  | Normal | 46.61 (37.76 to 55.47) | 52.20 (43.99 to 60.41) | 62.78 (55.21 to 70.35) | 57.84 (50.51 to 65.16) | 64.30 (58.60 to 70.01) | 75.65 (69.64 to 81.66) | **3.19 (0.54 to 5.85)** | **11.35 (3.06 to 19.63)** | 8.15 (-0.55 to 16.86) |
|  |  | Overweight | 31.90 (24.16 to 39.63) | 57.18 (48.87 to 65.49) | 57.61 (50.67 to 64.55) | 58.72 (51.83 to 65.62) | 64.27 (58.56 to 69.99) | 69.89 (63.61 to 76.17) | **3.79 (1.18 to 6.39)** | 5.62 (-2.90 to 14.13) | 1.83 (-7.07 to 10.73) |
|  |  | Obese | 36.41 (29.74 to 43.07) | 54.75 (48.64 to 60.87) | 50.00 (44.04 to 55.97) | 52.49 (46.81 to 58.17) | 52.94 (48.89 to 56.99) | 60.20 (55.29 to 65.12) | 0.73 (-1.25 to 2.71) | **7.27 (0.91 to 13.63)** | 6.54 (-0.12 to 13.20) |
|  | Control among participants with diabetes | Underweight | 49.20 (13.81 to 84.60) | 35.57 (6.82 to 64.31) | 36.57 (12.20 to 60.95) | 10.84 (0.00 to 26.30) | 48.86 (19.86 to 77.85) | 62.02 (19.65 to 100.00) | 0.14 (-10.50 to 10.78) | 13.16 (-38.14 to 64.47) | 13.02 (-39.37 to 65.42) |
|  |  | Normal | 41.05 (31.67 to 50.44) | 29.34 (21.73 to 36.94) | 31.47 (24.02 to 38.93) | 27.32 (21.37 to 33.26) | 30.35 (25.01 to 35.69) | 34.38 (27.91 to 40.85) | -0.81 (-3.31 to 1.70) | 4.03 (-4.36 to 12.42) | 4.83 (-3.92 to 13.59) |
|  |  | Overweight | 29.62 (20.49 to 38.75) | 31.10 (23.20 to 39.00) | 25.90 (18.98 to 32.82) | 17.42 (11.76 to 23.07) | 24.98 (19.71 to 30.25) | 30.70 (24.15 to 37.24) | -2.19 (-4.70 to 0.33) | 5.72 (-2.70 to 14.14) | 7.90 (-0.88 to 16.69) |
|  |  | Obese | 29.05 (21.95 to 36.15) | 30.50 (25.15 to 35.84) | 21.25 (15.48 to 27.01) | 25.94 (21.02 to 30.85) | 27.94 (24.37 to 31.50) | 26.01 (22.09 to 29.93) | -0.11 (-1.89 to 1.68) | -1.93 (-7.25 to 3.39) | -1.82 (-7.43 to 3.79) |
|  | Control among participants being treated | Underweight | 35.67 (0.00 to 90.84) | 31.26 (0.00 to 73.52) | 40.93 (1.82 to 80.04) | 19.44 (0.00 to 45.83) | 70.07 (28.17 to 100.00) | 63.83 (18.53 to 100.00) | 7.29 (-9.38 to 23.95) | -6.23 (-67.97 to 55.51) | -13.52 (-77.46 to 50.43) |
|  |  | Normal | 35.69 (21.51 to 49.87) | 26.75 (18.04 to 35.46) | 23.41 (15.80 to 31.01) | 25.32 (17.59 to 33.05) | 27.35 (21.00 to 33.70) | 33.74 (26.31 to 41.16) | 0.17 (-2.84 to 3.17) | 6.39 (-3.39 to 16.17) | 6.23 (-4.01 to 16.46) |
|  |  | Overweight | 12.87 (0.00 to 27.42) | 23.28 (13.56 to 32.99) | 26.57 (18.02 to 35.12) | 16.88 (10.69 to 23.06) | 23.17 (16.81 to 29.54) | 34.25 (26.11 to 42.39) | -0.48 (-3.78 to 2.81) | **11.08 (0.73 to 21.43)** | **11.56 (0.70 to 22.42)** |
|  |  | Obese | 23.87 (12.01 to 35.73) | 26.77 (19.52 to 34.02) | 20.02 (13.48 to 26.55) | 25.42 (19.07 to 31.77) | 26.92 (22.36 to 31.47) | 25.49 (20.79 to 30.19) | 0.70 (-1.72 to 3.12) | -1.43 (-7.99 to 5.14) | -2.13 (-9.12 to 4.87) |
| Female | Prevalence | Underweight | 6.27 (3.16 to 9.38) | 2.72 (0.76 to 4.69) | 3.51 (1.41 to 5.60) | 3.12 (1.35 to 4.89) | 2.28 (0.85 to 3.70) | 5.02 (2.36 to 7.67) | -0.39 (-1.05 to 0.27) | 2.74 (-0.28 to 5.76) | **3.13 (0.04 to 6.22)** |
|  |  | Normal | 4.73 (3.82 to 5.64) | 4.59 (3.82 to 5.37) | 5.80 (4.93 to 6.67) | 6.61 (5.69 to 7.52) | 6.45 (5.72 to 7.18) | 8.32 (7.26 to 9.38) | **0.59 (0.30 to 0.88)** | **1.86 (0.56 to 3.16)** | 1.27 (-0.06 to 2.60) |
|  |  | Overweight | 9.78 (8.08 to 11.48) | 9.24 (7.75 to 10.73) | 8.82 (7.40 to 10.24) | 13.81 (12.04 to 15.58) | 12.46 (10.98 to 13.93) | 11.92 (10.16 to 13.68) | **1.27 (0.71 to 1.82)** | -0.53 (-2.84 to 1.77) | -1.80 (-4.17 to 0.57) |
|  |  | Obese | 14.62 (12.95 to 16.30) | 15.60 (13.98 to 17.23) | 18.66 (16.90 to 20.42) | 19.46 (17.76 to 21.17) | 22.10 (20.53 to 23.66) | 21.07 (19.05 to 23.10) | **1.97 (1.37 to 2.57)** | -1.02 (-3.59 to 1.54) | **-2.99 (-5.63 to -0.36)** |
|  | Awareness | Underweight | 52.73 (26.44 to 79.03) | 98.95 (96.77 to 100.00) | 100.00 (100.00 to 100.00) | 49.04 (19.93 to 78.14) | 100.00 (100.00 to 100.00) | 82.18 (63.24 to 100.00) | -0.02 (-6.79 to 6.75) | -17.82 (-36.77 to 1.14) | -17.80 (-37.93 to 2.33) |
|  |  | Normal | 53.13 (43.38 to 62.88) | 88.37 (83.63 to 93.12) | 81.56 (75.69 to 87.44) | 77.19 (70.57 to 83.81) | 75.12 (69.68 to 80.57) | 81.69 (76.78 to 86.60) | -1.80 (-3.98 to 0.38) | 6.57 (-0.76 to 13.89) | **8.37 (0.72 to 16.01)** |
|  |  | Overweight | 66.42 (57.71 to 75.13) | 77.12 (69.48 to 84.76) | 74.53 (67.17 to 81.89) | 64.56 (57.88 to 71.23) | 71.39 (65.75 to 77.02) | 84.32 (79.22 to 89.43) | -1.67 (-4.12 to 0.79) | **12.94 (5.32 to 20.55)** | **14.60 (6.60 to 22.61)** |
|  |  | Obese | 54.66 (48.69 to 60.64) | 71.48 (66.02 to 76.95) | 62.18 (57.10 to 67.27) | 63.43 (58.74 to 68.13) | 67.83 (64.12 to 71.54) | 66.31 (61.74 to 70.89) | 0.36 (-1.37 to 2.08) | -1.52 (-7.42 to 4.38) | -1.87 (-8.02 to 4.27) |
|  | Treatment | Underweight | 33.00 (7.09 to 58.90) | 73.28 (39.76 to 100.00) | 86.52 (72.09 to 100.00) | 39.75 (11.50 to 68.00) | 100.00 (100.00 to 100.00) | 77.15 (56.40 to 97.91) | 6.41 (-2.11 to 14.92) | **-22.85 (-43.62 to -2.08)** | **-29.25 (-51.70 to -6.81)** |
|  |  | Normal | 48.25 (38.83 to 57.68) | 69.35 (61.84 to 76.85) | 70.95 (64.17 to 77.73) | 67.27 (60.12 to 74.43) | 67.29 (61.50 to 73.09) | 77.85 (72.74 to 82.97) | 0.43 (-2.12 to 2.98) | **10.56 (2.85 to 18.27)** | **10.13 (2.00 to 18.25)** |
|  |  | Overweight | 53.51 (44.14 to 62.89) | 58.90 (50.50 to 67.30) | 62.35 (54.18 to 70.52) | 58.01 (51.39 to 64.62) | 69.34 (63.59 to 75.09) | 82.06 (76.72 to 87.40) | **2.93 (0.31 to 5.55)** | **12.72 (4.87 to 20.58)** | **9.79 (1.51 to 18.07)** |
|  |  | Obese | 45.38 (39.13 to 51.64) | 62.00 (56.20 to 67.79) | 57.25 (52.14 to 62.36) | 59.47 (54.56 to 64.39) | 63.49 (59.62 to 67.36) | 63.22 (58.57 to 67.88) | **1.81 (0.01 to 3.62)** | -0.27 (-6.34 to 5.80) | -2.08 (-8.42 to 4.25) |
|  | Control among participants with diabetes | Underweight | 46.28 (14.81 to 77.74) | 26.27 (0.00 to 58.79) | 38.97 (2.33 to 75.62) | 33.37 (2.46 to 64.29) | 31.63 (0.43 to 62.84) | 21.67 (1.50 to 41.83) | -1.43 (-12.48 to 9.62) | -9.97 (-47.14 to 27.21) | -8.54 (-47.32 to 30.25) |
|  |  | Normal | 32.59 (23.50 to 41.68) | 26.63 (19.16 to 34.09) | 30.55 (23.00 to 38.10) | 24.35 (17.53 to 31.16) | 31.91 (26.03 to 37.78) | 32.56 (26.06 to 39.06) | 0.63 (-1.93 to 3.20) | 0.66 (-8.09 to 9.40) | 0.02 (-9.10 to 9.14) |
|  |  | Overweight | 30.95 (21.85 to 40.06) | 27.69 (19.89 to 35.50) | 26.48 (18.52 to 34.45) | 20.97 (15.18 to 26.76) | 24.43 (19.53 to 29.34) | 35.12 (27.16 to 43.08) | -1.58 (-3.96 to 0.81) | **10.69 (1.31 to 20.06)** | **12.26 (2.59 to 21.93)** |
|  |  | Obese | 21.44 (15.50 to 27.37) | 24.14 (18.79 to 29.48) | 20.69 (16.25 to 25.12) | 18.19 (13.72 to 22.66) | 24.86 (21.49 to 28.23) | 24.37 (20.36 to 28.37) | 0.36 (-1.28 to 2.00) | -0.49 (-5.72 to 4.74) | -0.85 (-6.33 to 4.64) |
|  | Control among participants being treated | Underweight | N/A | 16.30 (0.00 to 46.46) | 45.96 (6.32 to 85.61) | 55.37 (5.15 to 100.00) | 31.63 (0.43 to 62.84) | 20.88 (0.00 to 43.86) | 4.17 (-10.15 to 18.48) | -10.76 (-49.54 to 28.03) | -14.92 (-56.27 to 26.42) |
|  |  | Normal | 19.68 (7.61 to 31.76) | 22.70 (14.02 to 31.37) | 28.76 (19.34 to 38.19) | 21.95 (14.33 to 29.57) | 27.46 (21.12 to 33.80) | 32.97 (25.40 to 40.54) | 0.90 (-2.14 to 3.93) | 5.51 (-4.34 to 15.35) | 4.61 (-5.69 to 14.91) |
|  |  | Overweight | 21.57 (9.86 to 33.29) | 26.09 (15.55 to 36.62) | 28.06 (17.81 to 38.32) | 23.40 (15.32 to 31.49) | 28.24 (22.05 to 34.43) | 36.10 (27.40 to 44.80) | 0.58 (-2.66 to 3.82) | 7.86 (-2.84 to 18.56) | 7.28 (-3.90 to 18.47) |
|  |  | Obese | 25.89 (16.92 to 34.86) | 21.73 (15.61 to 27.85) | 24.30 (17.97 to 30.63) | 19.00 (13.30 to 24.70) | 24.97 (20.80 to 29.14) | 27.89 (22.71 to 33.07) | 0.42 (-1.64 to 2.48) | 2.92 (-3.75 to 9.58) | 2.50 (-4.48 to 9.47) |

Abbreviations: BMI, body mass index (calculated as weight in kilograms divided by height in meters squared); CI, confidence interval; KNHANES, Korea National Health and Nutrition Examination Survey.

Numbers in bold indicate a significant difference (P < 0.05).

^a^ BMI was divided into four groups according to Asian-Pacific guidelines: underweight (<18.5 kg/m^2^), normal (18.5-22.9 kg/m^2^), overweight (23.0–24.9 kg/m^2^), and obese (≥25 kg/m^2^).

^b^ All βs and β_diff_s were expressed by multiplying 100.

**Table S4.** National trends of the prevalence, awareness, treatment, control among participants with diabetes, and control among participants being treated by educational background among males and females before and during the COVID-19 pandemic (weighted % [95% CI]) based on data obtained from the KNHANES.

| Sex | Rate | Educational background | Before the pandemic | | | | | During the pandemic | Trends in the pre-pandemic,  β (95% CI) ^a^ | Trends in the pandemic,  β (95% CI) ^a^ | Trend differences, βdiff (95% CI) ^a^ |
| --- | --- | --- | --- | --- | --- | --- | --- | --- | --- | --- | --- |
|  |  |  | 1998-2005 | 2007-2009 | 2010-2012 | 2013-2015 | 2016-2019 | 2020-2022 |  |  |  |
| Male | Prevalence | Elementary school or lower | 11.76 (10.60 to 12.91) | 17.45 (14.98 to 19.92) | 21.47 (18.67 to 24.27) | 26.28 (22.71 to 29.85) | 26.18 (23.19 to 29.17) | 28.12 (23.79 to 32.44) | **3.83 (3.05 to 4.61)** | 1.94 (-3.34 to 7.22) | -1.89 (-7.23 to 3.45) |
|  |  | Middle school | 10.35 (9.17 to 11.53) | 16.83 (14.15 to 19.50) | 19.87 (16.75 to 22.99) | 22.08 (18.48 to 25.67) | 27.57 (24.44 to 30.71) | 30.21 (26.08 to 34.34) | **3.92 (3.08 to 4.75)** | 2.64 (-2.56 to 7.84) | -1.28 (-6.54 to 3.99) |
|  |  | High school | 6.34 (5.72 to 6.96) | 9.85 (8.42 to 11.27) | 12.76 (11.02 to 14.50) | 15.63 (13.75 to 17.52) | 19.15 (17.39 to 20.91) | 21.66 (19.48 to 23.83) | **3.14 (2.68 to 3.59)** | 2.50 (-0.31 to 5.32) | -0.63 (-3.48 to 2.22) |
|  |  | College or higher | 4.61 (4.03 to 5.20) | 7.65 (6.41 to 8.89) | 8.30 (7.03 to 9.57) | 9.95 (8.66 to 11.24) | 10.21 (9.19 to 11.23) | 13.18 (11.70 to 14.66) | **1.22 (0.89 to 1.56)** | **2.97 (1.18 to 4.76)** | 1.75 (-0.08 to 3.57) |
|  | Awareness | Elementary school or lower | 83.85 (79.86 to 87.84) | 75.53 (68.07 to 82.99) | 74.48 (67.90 to 81.06) | 72.46 (64.91 to 80.02) | 71.39 (65.93 to 76.85) | 77.95 (70.91 to 85.00) | **-2.47 (-4.41 to -0.54)** | 6.56 (-2.40 to 15.52) | 9.04 (-0.13 to 18.21) |
|  |  | Middle school | 85.51 (81.06 to 89.97) | 83.13 (76.47 to 89.79) | 69.20 (60.18 to 78.22) | 64.77 (55.80 to 73.75) | 75.50 (69.58 to 81.41) | 72.81 (65.35 to 80.27) | **-3.00 (-5.13 to -0.87)** | -2.68 (-12.19 to 6.82) | 0.32 (-9.42 to 10.06) |
|  |  | High school | 82.80 (78.89 to 86.71) | 66.54 (59.32 to 73.76) | 60.35 (53.30 to 67.40) | 58.55 (52.12 to 64.97) | 63.82 (58.83 to 68.82) | 71.71 (66.77 to 76.66) | **-2.97 (-4.84 to -1.09)** | **7.89 (0.86 to 14.91)** | **10.85 (3.58 to 18.12)** |
|  |  | College or higher | 82.96 (78.45 to 87.47) | 64.34 (55.78 to 72.90) | 57.98 (49.45 to 66.51) | 57.98 (51.07 to 64.90) | 53.44 (48.40 to 58.47) | 68.02 (62.92 to 73.12) | **-4.88 (-7.11 to -2.65)** | **14.59 (7.43 to 21.75)** | **19.46 (11.96 to 26.96)** |
|  | Treatment | Elementary school or lower | 44.32 (39.25 to 49.40) | 58.05 (49.34 to 66.75) | 67.38 (60.15 to 74.61) | 66.30 (58.44 to 74.15) | 67.05 (61.25 to 72.86) | 72.98 (65.48 to 80.47) | **4.69 (2.54 to 6.85)** | 5.92 (-3.59 to 15.43) | 1.23 (-8.52 to 10.98) |
|  |  | Middle school | 43.78 (37.63 to 49.92) | 64.58 (55.25 to 73.90) | 62.36 (53.31 to 71.41) | 61.25 (52.24 to 70.27) | 70.24 (63.91 to 76.58) | 69.16 (61.43 to 76.89) | **3.90 (1.40 to 6.40)** | -1.08 (-11.07 to 8.90) | -4.98 (-15.28 to 5.31) |
|  |  | High school | 39.80 (35.03 to 44.57) | 52.36 (44.89 to 59.82) | 50.61 (44.05 to 57.17) | 51.82 (45.47 to 58.18) | 57.53 (52.40 to 62.66) | 67.34 (62.03 to 72.66) | **3.07 (1.14 to 5.01)** | **9.81 (2.44 to 17.18)** | 6.74 (-0.88 to 14.36) |
|  |  | College or higher | 43.78 (37.47 to 50.09) | 47.16 (38.79 to 55.52) | 48.55 (40.35 to 56.75) | 49.45 (42.66 to 56.25) | 48.87 (43.80 to 53.94) | 61.78 (55.98 to 67.58) | 0.86 (-1.38 to 3.10) | **12.91 (5.20 to 20.62)** | **12.05 (4.02 to 20.08)** |
|  | Control among participants with diabetes | Elementary school or lower | 34.21 (25.17 to 43.26) | 28.84 (21.50 to 36.17) | 26.79 (19.76 to 33.82) | 24.55 (18.19 to 30.92) | 33.40 (27.36 to 39.43) | 30.95 (23.40 to 38.50) | 0.52 (-2.02 to 3.07) | -2.45 (-12.13 to 7.23) | -2.97 (-12.98 to 7.04) |
|  |  | Middle school | 39.82 (28.43 to 51.20) | 35.09 (25.92 to 44.25) | 26.86 (18.16 to 35.56) | 25.84 (17.42 to 34.26) | 27.75 (22.02 to 33.48) | 28.89 (21.53 to 36.26) | -2.33 (-5.27 to 0.61) | 1.14 (-8.21 to 10.50) | 3.47 (-6.34 to 13.28) |
|  |  | High school | 33.47 (24.66 to 42.28) | 31.11 (23.59 to 38.64) | 24.85 (17.95 to 31.75) | 23.04 (17.28 to 28.81) | 27.50 (23.23 to 31.77) | 30.19 (24.65 to 35.74) | -1.01 (-3.26 to 1.25) | 2.69 (-4.32 to 9.71) | 3.70 (-3.67 to 11.06) |
|  |  | College or higher | 24.98 (15.69 to 34.28) | 28.97 (20.61 to 37.32) | 24.83 (17.49 to 32.18) | 23.99 (18.18 to 29.81) | 26.71 (22.02 to 31.39) | 28.58 (23.47 to 33.69) | -0.34 (-2.90 to 2.22) | 1.87 (-5.06 to 8.80) | 2.21 (-5.18 to 9.60) |
|  | Control among participants being treated | Elementary school or lower | 29.28 (16.94 to 41.61) | 23.00 (14.97 to 31.04) | 28.14 (19.52 to 36.77) | 24.60 (17.13 to 32.07) | 30.46 (23.07 to 37.84) | 31.04 (22.32 to 39.76) | 1.44 (-1.67 to 4.56) | 0.58 (-10.87 to 12.04) | -0.86 (-12.73 to 11.01) |
|  |  | Middle school | 32.71 (14.51 to 50.90) | 27.20 (16.85 to 37.54) | 25.32 (15.72 to 34.92) | 25.12 (14.92 to 35.33) | 25.95 (19.40 to 32.49) | 26.41 (17.78 to 35.04) | -0.50 (-4.01 to 3.02) | 0.47 (-10.38 to 11.32) | 0.96 (-10.44 to 12.37) |
|  |  | High school | 28.21 (13.08 to 43.34) | 26.05 (16.52 to 35.57) | 19.07 (11.68 to 26.46) | 18.99 (12.37 to 25.61) | 24.13 (19.14 to 29.12) | 32.16 (25.44 to 38.88) | -0.09 (-2.97 to 2.78) | 8.03 (-0.37 to 16.43) | 8.12 (-0.76 to 17.00) |
|  |  | College or higher | 15.31 (3.01 to 27.61) | 27.33 (16.33 to 38.32) | 21.07 (12.61 to 29.53) | 25.48 (17.05 to 33.92) | 26.90 (20.16 to 33.65) | 29.36 (23.21 to 35.51) | 0.92 (-2.60 to 4.45) | 2.46 (-6.64 to 11.56) | 1.53 (-8.23 to 11.29) |
| Female | Prevalence | Elementary school or lower | 12.47 (11.64 to 13.30) | 18.40 (16.61 to 20.18) | 21.02 (19.14 to 22.90) | 25.06 (22.81 to 27.31) | 27.70 (25.80 to 29.60) | 29.29 (27.00 to 31.58) | **3.70 (3.19 to 4.21)** | 1.59 (-1.40 to 4.58) | -2.11 (-5.14 to 0.92) |
|  |  | Middle school | 6.17 (5.28 to 7.06) | 8.03 (6.38 to 9.68) | 13.02 (10.68 to 15.36) | 16.13 (13.46 to 18.81) | 19.10 (16.75 to 21.44) | 20.92 (17.90 to 23.95) | **3.41 (2.81 to 4.01)** | 1.83 (-2.00 to 5.66) | -1.58 (-5.46 to 2.30) |
|  |  | High school | 2.73 (2.33 to 3.14) | 4.96 (4.07 to 5.85) | 6.20 (5.08 to 7.32) | 7.91 (6.70 to 9.12) | 9.91 (8.75 to 11.06) | 12.21 (10.68 to 13.75) | **1.72 (1.42 to 2.02)** | **2.31 (0.37 to 4.24)** | 0.58 (-1.38 to 2.54) |
|  |  | College or higher | 1.40 (0.98 to 1.81) | 2.68 (1.81 to 3.55) | 2.70 (1.83 to 3.57) | 3.97 (3.15 to 4.79) | 3.70 (3.06 to 4.33) | 5.69 (4.61 to 6.76) | **0.53 (0.30 to 0.75)** | **1.99 (0.74 to 3.24)** | **1.47 (0.20 to 2.74)** |
|  | Awareness | Elementary school or lower | 86.41 (83.84 to 88.98) | 80.87 (76.74 to 85.00) | 75.34 (71.12 to 79.57) | 72.61 (68.30 to 76.93) | 77.92 (74.52 to 81.32) | 85.18 (81.82 to 88.54) | **-2.09 (-3.24 to -0.94)** | **7.26 (2.48 to 12.04)** | **9.36 (4.44 to 14.27)** |
|  |  | Middle school | 82.83 (77.42 to 88.23) | 74.33 (64.35 to 84.31) | 65.63 (56.39 to 74.88) | 66.37 (58.25 to 74.50) | 67.03 (60.08 to 73.98) | 74.94 (67.81 to 82.06) | **-3.01 (-5.53 to -0.50)** | 7.91 (-2.05 to 17.87) | **10.92 (0.65 to 21.20)** |
|  |  | High school | 75.52 (69.07 to 81.97) | 67.53 (58.69 to 76.37) | 61.20 (52.13 to 70.27) | 61.58 (54.39 to 68.77) | 64.52 (59.09 to 69.95) | 65.15 (58.60 to 71.70) | -1.62 (-3.95 to 0.70) | 0.63 (-7.85 to 9.11) | 2.25 (-6.54 to 11.05) |
|  |  | College or higher | 70.34 (56.84 to 83.84) | 67.25 (51.72 to 82.79) | 54.45 (37.66 to 71.24) | 51.16 (40.31 to 62.00) | 59.70 (50.98 to 68.42) | 67.55 (59.66 to 75.44) | -2.06 (-6.36 to 2.24) | 7.85 (-3.97 to 19.67) | 9.91 (-2.66 to 22.49) |
|  | Treatment | Elementary school or lower | 52.17 (48.62 to 55.73) | 68.77 (64.23 to 73.30) | 68.68 (64.40 to 72.96) | 66.95 (62.50 to 71.40) | 73.76 (69.99 to 77.53) | 82.16 (78.62 to 85.70) | **3.43 (2.13 to 4.72)** | **8.40 (3.24 to 13.56)** | 4.97 (-0.35 to 10.29) |
|  |  | Middle school | 53.71 (46.40 to 61.03) | 62.67 (51.99 to 73.34) | 59.37 (49.85 to 68.89) | 60.82 (52.29 to 69.35) | 62.12 (55.25 to 68.99) | 72.65 (65.36 to 79.94) | 1.19 (-1.46 to 3.83) | **10.53 (0.49 to 20.57)** | 9.34 (-1.04 to 19.73) |
|  |  | High school | 47.13 (40.01 to 54.26) | 50.87 (41.11 to 60.64) | 52.07 (42.69 to 61.44) | 57.71 (50.39 to 65.04) | 59.53 (54.03 to 65.02) | 62.17 (55.67 to 68.68) | **3.17 (0.72 to 5.62)** | 2.65 (-5.85 to 11.15) | -0.52 (-9.36 to 8.32) |
|  |  | College or higher | 43.69 (29.62 to 57.76) | 37.58 (21.55 to 53.61) | 38.04 (22.98 to 53.11) | 36.59 (25.71 to 47.48) | 54.64 (45.70 to 63.59) | 63.64 (55.38 to 71.90) | **4.67 (0.29 to 9.06)** | 9.00 (-3.20 to 21.20) | 4.32 (-8.64 to 17.29) |
|  | Control among participants with diabetes | Elementary school or lower | 25.64 (20.61 to 30.67) | 27.77 (22.62 to 32.92) | 28.27 (23.33 to 33.21) | 18.95 (14.41 to 23.50) | 27.69 (24.05 to 31.32) | 31.77 (27.03 to 36.50) | -0.64 (-2.27 to 1.00) | 4.08 (-1.91 to 10.07) | 4.72 (-1.50 to 10.93) |
|  |  | Middle school | 23.65 (13.07 to 34.24) | 31.07 (20.85 to 41.29) | 18.43 (10.73 to 26.14) | 20.42 (12.75 to 28.09) | 27.86 (21.93 to 33.80) | 19.18 (12.80 to 25.56) | 0.50 (-2.43 to 3.43) | -8.68 (-17.40 to 0.03) | -9.18 (-18.38 to 0.01) |
|  |  | High school | 32.95 (22.14 to 43.75) | 16.59 (9.03 to 24.15) | 21.51 (13.76 to 29.26) | 23.53 (16.58 to 30.48) | 21.22 (16.52 to 25.91) | 27.85 (21.59 to 34.11) | 0.33 (-2.09 to 2.76) | 6.63 (-1.19 to 14.46) | 6.30 (-1.89 to 14.49) |
|  |  | College or higher | 39.35 (18.41 to 60.30) | 25.47 (8.71 to 42.23) | 14.72 (4.49 to 24.94) | 21.81 (12.91 to 30.71) | 31.38 (22.67 to 40.09) | 32.26 (23.70 to 40.82) | 3.09 (-1.68 to 7.87) | 0.88 (-11.34 to 13.10) | -2.21 (-15.33 to 10.91) |
|  | Control among participants being treated | Elementary school or lower | 23.19 (16.38 to 30.00) | 24.17 (18.90 to 29.45) | 30.41 (24.38 to 36.45) | 18.60 (13.15 to 24.05) | 28.31 (24.11 to 32.50) | 33.48 (28.06 to 38.91) | 0.37 (-1.52 to 2.26) | 5.18 (-1.71 to 12.07) | 4.80 (-2.34 to 11.95) |
|  |  | Middle school | 21.28 (7.45 to 35.10) | 34.36 (20.60 to 48.13) | 23.40 (11.87 to 34.93) | 20.82 (11.51 to 30.13) | 27.94 (20.62 to 35.27) | 24.10 (16.04 to 32.16) | -0.63 (-4.60 to 3.34) | -3.84 (-14.76 to 7.08) | -3.21 (-14.83 to 8.41) |
|  |  | High school | 22.75 (5.86 to 39.64) | 10.94 (1.62 to 20.27) | 20.84 (10.99 to 30.69) | 28.10 (18.04 to 38.17) | 17.84 (12.41 to 23.27) | 26.95 (18.93 to 34.97) | 1.23 (-1.87 to 4.34) | 9.11 (-0.59 to 18.81) | 7.87 (-2.31 to 18.06) |
|  |  | College or higher | 51.07 (5.06 to 97.08) | 12.90 (0.00 to 30.11) | 11.86 (0.00 to 28.88) | 18.25 (4.84 to 31.65) | 35.34 (22.80 to 47.88) | 39.47 (27.45 to 51.48) | **7.78 (0.97 to 14.59)** | 4.13 (-13.18 to 21.44) | -3.65 (-22.25 to 14.95) |

Abbreviations: CI, confidence interval; KNHANES, Korea National Health and Nutrition Examination Survey.

Numbers in bold indicate a significant difference (P < 0.05).

^a^ All βs and β_diff_s were expressed by multiplying 100.

**Table S5.** National trends of the prevalence, awareness, treatment, control among participants with diabetes, and control among participants being treated by household income among males and females before and during the COVID-19 pandemic (weighted % [95% CI]) based on data obtained from the KNHANES.

| Sex | Rate | Household income | Before the pandemic | | | | | During the pandemic | Trends in the pre-pandemic,  β (95% CI) ^a^ | Trends in the pandemic,  β (95% CI) ^a^ | Trend differences, βdiff (95% CI) ^a^ |
| --- | --- | --- | --- | --- | --- | --- | --- | --- | --- | --- | --- |
|  |  |  | 1998-2005 | 2007-2009 | 2010-2012 | 2013-2015 | 2016-2019 | 2020-2022 |  |  |  |
| Male | Prevalence | Lowest quartile | 11.05 (9.98 to 12.12) | 18.23 (15.61 to 20.84) | 21.08 (18.42 to 23.74) | 25.97 (22.58 to 29.36) | 28.78 (26.12 to 31.43) | 26.77 (23.61 to 29.92) | **4.32 (3.60 to 5.03)** | -2.01 (-6.14 to 2.12) | **-6.33 (-10.52 to -2.14)** |
|  |  | Second quartile | 6.70 (5.93 to 7.47) | 11.42 (9.56 to 13.28) | 11.45 (9.79 to 13.10) | 17.27 (15.01 to 19.53) | 17.06 (15.29 to 18.83) | 21.29 (18.73 to 23.85) | **2.59 (2.08 to 3.11)** | **4.23 (1.13 to 7.33)** | 1.63 (-1.51 to 4.78) |
|  |  | Third quartile | 5.94 (5.28 to 6.60) | 9.46 (7.88 to 11.04) | 11.37 (9.59 to 13.16) | 11.49 (9.76 to 13.22) | 13.71 (12.17 to 15.24) | 16.35 (14.34 to 18.36) | **1.68 (1.23 to 2.13)** | **2.65 (0.13 to 5.17)** | 0.97 (-1.59 to 3.53) |
|  |  | Highest quartile | 6.06 (5.41 to 6.71) | 8.79 (7.31 to 10.28) | 11.57 (9.74 to 13.41) | 11.65 (10.09 to 13.22) | 11.93 (10.66 to 13.19) | 14.63 (12.86 to 16.40) | **1.34 (0.95 to 1.73)** | **2.70 (0.54 to 4.86)** | 1.36 (-0.83 to 3.56) |
|  | Awareness | Lowest quartile | 87.88 (84.45 to 91.32) | 77.21 (70.61 to 83.81) | 71.76 (64.68 to 78.84) | 69.73 (62.31 to 77.15) | 68.71 (63.82 to 73.59) | 73.83 (67.45 to 80.21) | **-3.98 (-5.71 to -2.25)** | 5.12 (-2.90 to 13.14) | **9.10 (0.90 to 17.30)** |
|  |  | Second quartile | 83.80 (79.24 to 88.35) | 74.27 (66.52 to 82.02) | 65.57 (57.78 to 73.37) | 62.59 (55.55 to 69.64) | 61.55 (56.08 to 67.02) | 72.44 (66.69 to 78.19) | **-4.85 (-6.95 to -2.75)** | **10.89 (2.94 to 18.83)** | **15.74 (7.52 to 23.96)** |
|  |  | Third quartile | 81.71 (77.22 to 86.20) | 66.00 (57.54 to 74.46) | 56.86 (48.49 to 65.24) | 58.61 (50.38 to 66.84) | 60.64 (54.97 to 66.31) | 69.65 (63.57 to 75.73) | **-3.17 (-5.44 to -0.90)** | **9.01 (0.69 to 17.33)** | **12.18 (3.55 to 20.81)** |
|  |  | Highest quartile | 79.73 (75.24 to 84.22) | 67.96 (60.01 to 75.91) | 63.96 (56.36 to 71.57) | 57.59 (50.13 to 65.05) | 62.80 (57.10 to 68.50) | 69.37 (63.97 to 74.77) | **-3.30 (-5.46 to -1.14)** | 6.57 (-1.24 to 14.38) | **9.87 (1.77 to 17.98)** |
|  | Treatment | Lowest quartile | 44.38 (39.63 to 49.13) | 60.68 (52.96 to 68.40) | 63.05 (55.63 to 70.47) | 64.84 (57.30 to 72.38) | 61.43 (56.27 to 66.58) | 67.56 (60.59 to 74.53) | **2.74 (0.80 to 4.68)** | 6.13 (-2.53 to 14.80) | 3.39 (-5.49 to 12.27) |
|  |  | Second quartile | 40.73 (34.86 to 46.60) | 54.73 (47.01 to 62.46) | 55.68 (48.08 to 63.28) | 55.40 (48.24 to 62.55) | 56.62 (50.89 to 62.35) | 68.19 (62.25 to 74.14) | 2.16 (-0.03 to 4.35) | **11.58 (3.31 to 19.84)** | **9.42 (0.86 to 17.97)** |
|  |  | Third quartile | 43.61 (37.89 to 49.33) | 50.12 (41.21 to 59.02) | 48.61 (40.58 to 56.65) | 51.47 (43.26 to 59.67) | 57.37 (51.56 to 63.19) | 62.57 (55.74 to 69.40) | **2.90 (0.54 to 5.25)** | 5.20 (-3.78 to 14.17) | 2.30 (-6.98 to 11.57) |
|  |  | Highest quartile | 41.02 (35.50 to 46.55) | 53.45 (45.10 to 61.80) | 56.46 (48.85 to 64.08) | 50.29 (42.87 to 57.72) | 57.04 (51.19 to 62.88) | 66.16 (60.66 to 71.67) | 2.02 (-0.24 to 4.28) | **9.13 (1.13 to 17.12)** | 7.11 (-1.20 to 15.41) |
|  | Control among participants with diabetes | Lowest quartile | 32.25 (23.56 to 40.93) | 37.69 (29.70 to 45.68) | 28.00 (21.33 to 34.66) | 26.45 (19.79 to 33.12) | 31.61 (26.57 to 36.65) | 30.83 (24.38 to 37.28) | -1.35 (-3.80 to 1.11) | -0.78 (-8.97 to 7.40) | 0.56 (-7.98 to 9.11) |
|  |  | Second quartile | 33.00 (23.51 to 42.48) | 30.32 (23.01 to 37.63) | 27.80 (19.94 to 35.66) | 18.65 (13.44 to 23.86) | 25.66 (20.91 to 30.41) | 32.73 (26.64 to 38.82) | -2.09 (-4.46 to 0.27) | 7.07 (-0.66 to 14.80) | **9.17 (1.08 to 17.25)** |
|  |  | Third quartile | 31.80 (21.92 to 41.68) | 27.26 (19.11 to 35.40) | 24.30 (16.62 to 31.97) | 30.03 (22.62 to 37.43) | 28.05 (22.94 to 33.16) | 27.04 (21.53 to 32.55) | 0.53 (-2.09 to 3.15) | -1.00 (-8.52 to 6.51) | -1.53 (-9.49 to 6.42) |
|  |  | Highest quartile | 34.11 (24.89 to 43.34) | 28.13 (19.50 to 36.77) | 22.97 (15.97 to 29.96) | 22.22 (15.71 to 28.72) | 27.38 (22.07 to 32.68) | 28.05 (22.06 to 34.03) | -0.41 (-3.06 to 2.23) | 0.67 (-7.35 to 8.69) | 1.08 (-7.36 to 9.53) |
|  | Control among participants being treated | Lowest quartile | 29.26 (17.21 to 41.31) | 33.85 (24.35 to 43.35) | 21.16 (14.39 to 27.93) | 27.89 (20.49 to 35.28) | 31.73 (25.30 to 38.17) | 32.45 (25.11 to 39.78) | 0.46 (-2.66 to 3.57) | 0.71 (-9.05 to 10.47) | 0.26 (-9.99 to 10.50) |
|  |  | Second quartile | 19.45 (7.44 to 31.47) | 28.61 (18.54 to 38.68) | 25.02 (15.79 to 34.25) | 13.62 (8.08 to 19.15) | 26.23 (20.01 to 32.45) | 33.96 (26.54 to 41.39) | -0.90 (-4.16 to 2.35) | 7.73 (-1.95 to 17.41) | 8.63 (-1.58 to 18.84) |
|  |  | Third quartile | 35.33 (17.40 to 53.27) | 20.17 (11.63 to 28.72) | 23.70 (14.21 to 33.19) | 34.31 (23.67 to 44.95) | 24.53 (18.37 to 30.69) | 28.31 (21.47 to 35.14) | 1.24 (-1.94 to 4.42) | 3.78 (-5.43 to 12.99) | 2.54 (-7.20 to 12.29) |
|  |  | Highest quartile | 22.73 (8.89 to 36.58) | 20.13 (10.13 to 30.13) | 22.74 (14.04 to 31.45) | 18.46 (10.27 to 26.65) | 22.94 (16.73 to 29.15) | 27.02 (19.56 to 34.47) | 0.42 (-2.85 to 3.69) | 4.08 (-5.64 to 13.80) | 3.66 (-6.60 to 13.91) |
| Female | Prevalence | Lowest quartile | 9.91 (9.04 to 10.77) | 16.78 (14.71 to 18.84) | 19.47 (17.37 to 21.58) | 22.30 (19.92 to 24.67) | 24.04 (22.16 to 25.92) | 26.17 (23.80 to 28.53) | **3.35 (2.82 to 3.87)** | 2.13 (-0.89 to 5.14) | -1.22 (-4.28 to 1.84) |
|  |  | Second quartile | 6.33 (5.62 to 7.04) | 10.80 (9.28 to 12.31) | 10.58 (9.17 to 12.00) | 13.47 (11.94 to 15.00) | 13.07 (11.74 to 14.40) | 14.48 (12.70 to 16.26) | **1.52 (1.13 to 1.92)** | 1.41 (-0.82 to 3.65) | -0.11 (-2.38 to 2.16) |
|  |  | Third quartile | 4.96 (4.36 to 5.56) | 5.57 (4.44 to 6.69) | 7.98 (6.57 to 9.39) | 8.51 (7.19 to 9.82) | 9.08 (7.89 to 10.27) | 10.25 (8.65 to 11.84) | **1.11 (0.76 to 1.45)** | 1.17 (-0.83 to 3.17) | 0.06 (-1.96 to 2.09) |
|  |  | Highest quartile | 3.82 (3.26 to 4.38) | 5.34 (4.25 to 6.44) | 5.64 (4.50 to 6.78) | 6.59 (5.41 to 7.77) | 6.99 (5.98 to 8.00) | 7.57 (6.27 to 8.88) | **0.73 (0.42 to 1.03)** | 0.58 (-1.07 to 2.23) | -0.15 (-1.82 to 1.53) |
|  | Awareness | Lowest quartile | 84.77 (81.42 to 88.11) | 84.12 (79.09 to 89.15) | 75.31 (70.26 to 80.36) | 74.93 (69.70 to 80.16) | 76.63 (72.81 to 80.45) | 81.90 (78.11 to 85.68) | **-2.25 (-3.62 to -0.89)** | 5.27 (-0.09 to 10.64) | **7.53 (1.99 to 13.07)** |
|  |  | Second quartile | 85.13 (81.17 to 89.10) | 72.63 (66.00 to 79.25) | 69.04 (61.76 to 76.32) | 67.96 (61.83 to 74.10) | 70.26 (65.14 to 75.37) | 68.84 (62.87 to 74.81) | **-2.36 (-4.24 to -0.48)** | -1.42 (-9.28 to 6.44) | 0.95 (-7.14 to 9.03) |
|  |  | Third quartile | 80.99 (76.09 to 85.89) | 69.97 (60.89 to 79.04) | 68.73 (60.97 to 76.48) | 59.96 (52.28 to 67.64) | 65.53 (59.30 to 71.77) | 73.74 (67.82 to 79.67) | **-3.36 (-5.62 to -1.09)** | 8.21 (-0.41 to 16.83) | **11.57 (2.65 to 20.48)** |
|  |  | Highest quartile | 81.58 (75.85 to 87.30) | 74.77 (67.16 to 82.39) | 56.55 (45.65 to 67.44) | 55.83 (46.78 to 64.88) | 64.36 (56.82 to 71.91) | 70.14 (62.22 to 78.06) | **-4.12 (-6.67 to -1.56)** | 5.78 (-5.22 to 16.78) | 9.89 (-1.40 to 21.19) |
|  | Treatment | Lowest quartile | 50.82 (46.14 to 55.50) | 69.80 (63.82 to 75.79) | 68.59 (63.34 to 73.83) | 68.25 (62.61 to 73.89) | 70.63 (66.26 to 74.99) | 77.92 (73.94 to 81.90) | **2.79 (1.18 to 4.39)** | **7.29 (1.40 to 13.18)** | 4.50 (-1.60 to 10.61) |
|  |  | Second quartile | 53.86 (48.11 to 59.62) | 60.79 (53.36 to 68.23) | 61.90 (54.55 to 69.26) | 62.50 (56.06 to 68.94) | 67.42 (62.20 to 72.63) | 66.85 (60.76 to 72.95) | **2.61 (0.57 to 4.65)** | -0.56 (-8.59 to 7.47) | -3.17 (-11.46 to 5.11) |
|  |  | Third quartile | 52.87 (46.64 to 59.10) | 59.59 (50.22 to 68.97) | 58.43 (49.58 to 67.28) | 53.49 (45.67 to 61.31) | 61.72 (55.20 to 68.24) | 70.82 (64.65 to 77.00) | 1.08 (-1.32 to 3.49) | **9.11 (0.13 to 18.09)** | 8.02 (-1.28 to 17.32) |
|  |  | Highest quartile | 46.29 (39.25 to 53.34) | 53.51 (43.38 to 63.65) | 48.66 (38.11 to 59.21) | 49.73 (40.89 to 58.57) | 59.22 (51.56 to 66.87) | 67.12 (59.20 to 75.04) | 2.33 (-0.47 to 5.13) | 7.90 (-3.16 to 18.96) | 5.57 (-5.84 to 16.98) |
|  | Control among participants with diabetes | Lowest quartile | 25.60 (19.02 to 32.19) | 27.40 (21.17 to 33.64) | 30.97 (24.72 to 37.21) | 20.59 (15.34 to 25.84) | 28.42 (24.25 to 32.59) | 30.56 (25.85 to 35.28) | -0.33 (-2.29 to 1.62) | 2.15 (-4.17 to 8.46) | 2.48 (-4.13 to 9.09) |
|  |  | Second quartile | 32.16 (23.02 to 41.31) | 25.21 (18.58 to 31.83) | 21.80 (15.20 to 28.40) | 17.19 (12.16 to 22.21) | 27.73 (22.52 to 32.93) | 25.03 (19.14 to 30.93) | 0.02 (-2.29 to 2.33) | -2.69 (-10.58 to 5.20) | -2.71 (-10.93 to 5.51) |
|  |  | Third quartile | 23.84 (14.80 to 32.87) | 23.81 (13.67 to 33.95) | 18.99 (12.15 to 25.83) | 20.43 (13.57 to 27.28) | 21.96 (16.64 to 27.29) | 31.19 (24.21 to 38.17) | -0.24 (-2.98 to 2.50) | **9.23 (0.44 to 18.01)** | **9.47 (0.26 to 18.67)** |
|  |  | Highest quartile | 27.25 (16.50 to 37.99) | 26.54 (16.91 to 36.18) | 20.73 (12.24 to 29.23) | 26.51 (18.04 to 34.99) | 25.82 (19.18 to 32.45) | 27.70 (19.58 to 35.83) | 0.31 (-2.73 to 3.36) | 1.88 (-8.60 to 12.37) | 1.57 (-9.35 to 12.49) |
|  | Control among participants being treated | Lowest quartile | 23.50 (14.38 to 32.62) | 23.60 (17.11 to 30.09) | 35.77 (27.82 to 43.72) | 20.10 (14.03 to 26.17) | 29.49 (24.47 to 34.51) | 32.49 (26.98 to 37.99) | 0.46 (-1.89 to 2.82) | 3.00 (-4.49 to 10.48) | 2.53 (-5.31 to 10.38) |
|  |  | Second quartile | 29.45 (15.31 to 43.60) | 23.22 (15.26 to 31.18) | 23.55 (15.21 to 31.90) | 21.42 (14.44 to 28.39) | 26.85 (20.77 to 32.92) | 27.77 (20.32 to 35.22) | 0.65 (-2.19 to 3.49) | 0.92 (-8.71 to 10.56) | 0.27 (-9.77 to 10.32) |
|  |  | Third quartile | 24.41 (10.62 to 38.19) | 20.95 (9.29 to 32.62) | 13.41 (6.12 to 20.69) | 22.87 (12.77 to 32.97) | 23.23 (16.34 to 30.12) | 35.14 (26.24 to 44.03) | 1.58 (-1.95 to 5.12) | **11.91 (0.63 to 23.19)** | 10.33 (-1.49 to 22.15) |
|  |  | Highest quartile | 11.30 (0.00 to 23.50) | 23.22 (10.35 to 36.08) | 27.72 (13.97 to 41.46) | 20.89 (9.70 to 32.09) | 21.57 (13.92 to 29.23) | 28.99 (18.52 to 39.45) | -0.38 (-4.35 to 3.59) | 7.41 (-5.54 to 20.36) | 7.80 (-5.75 to 21.34) |

Abbreviations: CI, confidence interval; KNHANES, Korea National Health and Nutrition Examination Survey.

Numbers in bold indicate a significant difference (P < 0.05).

^a^ All βs and β_diff_s were expressed by multiplying 100.

**Table S6.** National trends of the prevalence, awareness, treatment, control among participants with diabetes, and control among participants being treated by smoking status among males and females before and during the COVID-19 pandemic (weighted % [95% CI]) based on data obtained from the KNHANES.

| Sex | Rate | Smoking status | Before the pandemic | | | | | During the pandemic | Trends in the pre-pandemic,  β (95% CI) ^a^ | Trends in the pandemic,  β (95% CI) ^a^ | Trend differences, βdiff (95% CI) ^a^ |
| --- | --- | --- | --- | --- | --- | --- | --- | --- | --- | --- | --- |
|  |  |  | 1998-2005 | 2007-2009 | 2010-2012 | 2013-2015 | 2016-2019 | 2020-2022 |  |  |  |
| Male | Prevalence | Smoker | 10.38 (9.42 to 11.34) | 10.87 (9.92 to 11.83) | 13.34 (12.22 to 14.45) | 15.32 (14.10 to 16.53) | 16.78 (15.75 to 17.81) | 19.25 (17.92 to 20.58) | **1.86 (1.49 to 2.24)** | **2.47 (0.79 to 4.15)** | 0.61 (-1.11 to 2.33) |
|  |  | Non-smoker | 7.49 (5.78 to 9.19) | 11.66 (9.34 to 13.97) | 10.42 (8.26 to 12.58) | 13.45 (11.30 to 15.60) | 12.37 (10.61 to 14.13) | 13.91 (11.81 to 16.01) | **0.75 (0.01 to 1.48)** | 1.54 (-1.20 to 4.29) | 0.80 (-2.04 to 3.64) |
|  | Awareness | Smoker | 64.18 (59.91 to 68.44) | 69.85 (65.50 to 74.21) | 65.61 (61.38 to 69.83) | 60.96 (56.89 to 65.03) | 63.64 (60.64 to 66.63) | 72.63 (69.36 to 75.90) | **-1.66 (-3.03 to -0.29)** | **8.99 (4.57 to 13.41)** | **10.65 (6.02 to 15.28)** |
|  |  | Non-smoker | 64.81 (53.43 to 76.19) | 78.69 (69.36 to 88.02) | 55.88 (44.31 to 67.46) | 67.57 (59.71 to 75.43) | 61.63 (53.89 to 69.36) | 63.17 (55.20 to 71.15) | -3.23 (-6.64 to 0.18) | 1.55 (-9.56 to 12.66) | 4.78 (-6.84 to 16.40) |
|  | Treatment | Smoker | 37.19 (32.58 to 41.79) | 52.94 (48.22 to 57.65) | 57.06 (53.02 to 61.11) | 54.08 (49.90 to 58.25) | 58.27 (55.15 to 61.38) | 67.58 (63.97 to 71.19) | **2.36 (0.91 to 3.80)** | **9.32 (4.56 to 14.07)** | **6.96 (1.99 to 11.93)** |
|  |  | Non-smoker | 49.80 (37.95 to 61.65) | 63.48 (53.52 to 73.44) | 47.23 (35.83 to 58.63) | 61.99 (53.90 to 70.09) | 56.95 (49.39 to 64.50) | 57.84 (49.84 to 65.84) | 0.02 (-3.45 to 3.49) | 0.89 (-10.11 to 11.90) | 0.87 (-10.67 to 12.41) |
|  | Control among participants with diabetes | Smoker | 35.14 (29.61 to 40.66) | 30.03 (25.81 to 34.25) | 25.43 (21.37 to 29.48) | 23.06 (19.56 to 26.55) | 28.77 (25.98 to 31.57) | 29.51 (26.15 to 32.86) | -0.62 (-1.99 to 0.76) | 0.74 (-3.63 to 5.11) | 1.36 (-3.23 to 5.94) |
|  |  | Non-smoker | 21.03 (9.11 to 32.95) | 34.42 (24.14 to 44.70) | 26.94 (16.46 to 37.43) | 28.63 (20.96 to 36.31) | 24.61 (18.53 to 30.68) | 29.11 (21.26 to 36.95) | -2.17 (-5.48 to 1.15) | 4.50 (-5.44 to 14.44) | 6.66 (-3.82 to 17.14) |
|  | Control among participants being treated | Smoker | 27.64 (19.39 to 35.88) | 26.54 (21.08 to 31.99) | 22.73 (18.39 to 27.08) | 22.23 (17.84 to 26.62) | 26.40 (22.90 to 29.90) | 30.49 (26.33 to 34.64) | 0.14 (-1.64 to 1.93) | 4.09 (-1.35 to 9.52) | 3.94 (-1.78 to 9.66) |
|  |  | Non-smoker | 22.79 (5.18 to 40.40) | 23.35 (12.73 to 33.96) | 26.24 (12.49 to 39.98) | 26.66 (16.66 to 36.66) | 25.69 (17.87 to 33.51) | 28.26 (18.72 to 37.81) | 0.75 (-3.15 to 4.64) | 2.58 (-9.78 to 14.93) | 1.83 (-11.12 to 14.78) |
| Female | Prevalence | Smoker | 9.84 (7.34 to 12.34) | 10.67 (8.23 to 13.11) | 9.13 (7.20 to 11.05) | 10.25 (7.89 to 12.61) | 9.27 (7.48 to 11.06) | 10.25 (8.09 to 12.41) | -0.26 (-1.05 to 0.53) | 0.98 (-1.82 to 3.78) | 1.24 (-1.67 to 4.15) |
|  |  | Non-smoker | 7.95 (7.24 to 8.65) | 8.85 (8.07 to 9.62) | 10.63 (9.76 to 11.49) | 12.02 (11.15 to 12.89) | 12.73 (11.93 to 13.53) | 13.55 (12.53 to 14.57) | **1.28 (1.00 to 1.57)** | 0.82 (-0.49 to 2.13) | -0.46 (-1.80 to 0.88) |
|  | Awareness | Smoker | 58.46 (44.86 to 72.06) | 74.17 (63.71 to 84.64) | 76.42 (67.20 to 85.63) | 69.95 (58.93 to 80.97) | 68.74 (59.58 to 77.90) | 65.26 (55.49 to 75.03) | -0.89 (-4.67 to 2.89) | -3.48 (-16.89 to 9.93) | -2.58 (-16.51 to 11.35) |
|  |  | Non-smoker | 64.03 (59.46 to 68.61) | 77.20 (73.37 to 81.04) | 68.90 (65.07 to 72.73) | 66.69 (63.25 to 70.13) | 70.92 (68.15 to 73.68) | 75.50 (72.56 to 78.45) | -0.87 (-2.11 to 0.37) | **4.59 (0.55 to 8.63)** | **5.46 (1.23 to 9.68)** |
|  | Treatment | Smoker | 49.85 (36.64 to 63.06) | 61.56 (50.87 to 72.24) | 65.32 (54.81 to 75.82) | 55.47 (43.35 to 67.59) | 63.43 (53.91 to 72.96) | 58.89 (48.95 to 68.83) | 0.51 (-3.40 to 4.42) | -4.54 (-18.32 to 9.23) | -5.05 (-19.37 to 9.26) |
|  |  | Non-smoker | 48.73 (44.06 to 53.40) | 62.94 (58.71 to 67.18) | 61.55 (57.57 to 65.54) | 61.28 (57.82 to 64.74) | 66.39 (63.41 to 69.36) | 72.77 (69.69 to 75.85) | **2.05 (0.72 to 3.38)** | **6.38 (2.11 to 10.65)** | 4.34 (-0.14 to 8.81) |
|  | Control among participants with diabetes | Smoker | 22.55 (9.40 to 35.69) | 25.18 (15.50 to 34.85) | 30.27 (18.22 to 42.32) | 28.68 (16.86 to 40.49) | 27.45 (18.11 to 36.78) | 25.48 (16.16 to 34.79) | 0.78 (-3.01 to 4.57) | -1.97 (-15.19 to 11.25) | -2.75 (-16.50 to 11.00) |
|  |  | Non-smoker | 27.01 (22.49 to 31.53) | 26.06 (21.72 to 30.41) | 23.76 (19.92 to 27.60) | 19.80 (16.58 to 23.02) | 26.39 (23.75 to 29.03) | 29.05 (25.70 to 32.40) | -0.12 (-1.43 to 1.19) | 2.66 (-1.62 to 6.93) | 2.78 (-1.69 to 7.25) |
|  | Control among participants being treated | Smoker | 12.53 (0.00 to 26.18) | 27.00 (16.02 to 37.97) | 33.59 (17.16 to 50.03) | 30.05 (13.87 to 46.23) | 29.70 (17.63 to 41.77) | 22.95 (11.86 to 34.03) | 1.30 (-3.49 to 6.08) | -6.75 (-23.16 to 9.66) | -8.05 (-25.14 to 9.04) |
|  |  | Non-smoker | 23.96 (17.49 to 30.43) | 22.39 (17.55 to 27.24) | 26.08 (21.04 to 31.12) | 20.30 (16.23 to 24.37) | 26.17 (23.03 to 29.31) | 31.96 (27.88 to 36.04) | 0.61 (-0.97 to 2.20) | **5.79 (0.64 to 10.94)** | 5.18 (-0.22 to 10.57) |

Abbreviations: CI, confidence interval; KNHANES, Korea National Health and Nutrition Examination Survey.

Numbers in bold indicate a significant difference (P < 0.05).

^a^ All βs and β_diff_s were expressed by multiplying 100.

**Table S7.** Weighted odds ratios of prevalence, awareness, treatment, control among participants with diabetes, and control among participants being treated by age among males and females based on data obtained from the KNHANES.

| Sex | Rate | Age, years | Overall  (1998–2022) | | Before the pandemic  (1998–2019) | | During the pandemic  (2020–2022) | | Ratio of ORs during the pandemic compared to before the pandemic (reference) | |
| --- | --- | --- | --- | --- | --- | --- | --- | --- | --- | --- |
|  |  |  | Weighted ORs  (95% CI) | P-value | Weighted ORs  (95% CI) | P-value | Weighted ORs  (95% CI) | P-value | Weighted ratio of ORs  (95% CI) | P-value |
| Male | Prevalence | 30-39 | 1.00 (ref) |  | 1.00 (ref) |  | 1.00 (ref) |  | 1.00 (ref) |  |
|  |  | 40–49 | **3.11 (2.59 to 3.72)** | **<.001** | **3.19 (2.62 to 3.90)** | **<.001** | **2.81 (1.86 to 4.25)** | **<.001** | 0.88 (0.56 to 1.39) | 0.585 |
|  |  | 50–59 | **6.54 (5.53 to 7.72)** | **<.001** | **6.45 (5.37 to 7.74)** | **<.001** | **6.57 (4.48 to 9.64)** | **<.001** | 1.02 (0.67 to 1.56) | 0.931 |
|  |  | 60–69 | **10.02 (8.49 to 11.82)** | **<.001** | **10.56 (8.80 to 12.67)** | **<.001** | **8.03 (5.52 to 11.70)** | **<.001** | 0.76 (0.50 to 1.15) | 0.199 |
|  |  | ≥70 | **9.96 (8.43 to 11.76)** | **<.001** | **10.03 (8.35 to 12.04)** | **<.001** | **9.15 (6.24 to 13.42)** | **<.001** | 0.91 (0.60 to 1.40) | 0.672 |
|  | Awareness | 30-39 | 1.00 (ref) |  | 1.00 (ref) |  | 1.00 (ref) |  | 1.00 (ref) |  |
|  |  | 40–49 | **1.61 (1.15 to 2.27)** | **0.006** | **1.83 (1.26 to 2.67)** | **0.002** | 1.18 (0.54 to 2.57) | 0.682 | 0.64 (0.27 to 1.53) | 0.317 |
|  |  | 50–59 | **3.29 (2.39 to 4.54)** | **<.001** | **4.05 (2.84 to 5.77)** | **<.001** | 1.74 (0.83 to 3.65) | 0.146 | **0.43 (0.19 to 0.98)** | **0.044** |
|  |  | 60–69 | **5.26 (3.80 to 7.27)** | **<.001** | **7.00 (4.89 to 10.01)** | **<.001** | **2.23 (1.06 to 4.68)** | **0.034** | **0.32 (0.14 to 0.72)** | **0.006** |
|  |  | ≥70 | **6.08 (4.36 to 8.48)** | **<.001** | **7.23 (5.03 to 10.38)** | **<.001** | **3.53 (1.62 to 7.67)** | **0.002** | 0.49 (0.21 to 1.15) | 0.100 |
|  | Treatment | 30-39 | 1.00 (ref) |  | 1.00 (ref) |  | 1.00 (ref) |  | 1.00 (ref) |  |
|  |  | 40–49 | **1.50 (1.06 to 2.13)** | **0.024** | **1.57 (1.05 to 2.34)** | **0.027** | 1.46 (0.70 to 3.06) | 0.319 | 0.93 (0.40 to 2.16) | 0.865 |
|  |  | 50–59 | **3.09 (2.21 to 4.32)** | **<.001** | **3.54 (2.44 to 5.13)** | **<.001** | **2.09 (1.01 to 4.33)** | **0.047** | 0.59 (0.26 to 1.34) | 0.207 |
|  |  | 60–69 | **4.86 (3.48 to 6.80)** | **<.001** | **6.15 (4.22 to 8.97)** | **<.001** | **2.48 (1.21 to 5.08)** | **0.014** | **0.40 (0.18 to 0.91)** | **0.028** |
|  |  | ≥70 | **6.07 (4.33 to 8.51)** | **<.001** | **6.89 (4.73 to 10.04)** | **<.001** | **4.20 (2.02 to 8.75)** | **<.001** | 0.61 (0.27 to 1.39) | 0.240 |
|  | Control among participants with diabetes | 30-39 | 1.00 (ref) |  | 1.00 (ref) |  | 1.00 (ref) |  | 1.00 (ref) |  |
|  |  | 40–49 | 1.11 (0.74 to 1.67) | 0.603 | 1.06 (0.69 to 1.64) | 0.791 | 1.32 (0.47 to 3.70) | 0.595 | 1.25 (0.41 to 3.81) | 0.701 |
|  |  | 50–59 | 1.28 (0.86 to 1.89) | 0.224 | 1.15 (0.75 to 1.75) | 0.518 | 1.82 (0.67 to 4.95) | 0.244 | 1.58 (0.53 to 4.69) | 0.410 |
|  |  | 60–69 | 1.30 (0.89 to 1.91) | 0.178 | 1.10 (0.73 to 1.65) | 0.660 | 2.18 (0.81 to 5.88) | 0.124 | 1.99 (0.68 to 5.82) | 0.209 |
|  |  | ≥70 | **1.62 (1.11 to 2.38)** | **0.014** | 1.44 (0.95 to 2.18) | 0.086 | 2.38 (0.90 to 6.34) | 0.082 | 1.66 (0.57 to 4.80) | 0.352 |
|  | Control among participants being treated | 30-39 | 1.00 (ref) |  | 1.00 (ref) |  | 1.00 (ref) |  | 1.00 (ref) |  |
|  |  | 40–49 | 2.21 (0.98 to 5.02) | 0.057 | 1.21 (0.51 to 2.86) | 0.668 | N/A |  | N/A |  |
|  |  | 50–59 | **2.20 (1.00 to 4.82)** | **0.050** | 1.21 (0.54 to 2.73) | 0.642 | N/A |  | N/A |  |
|  |  | 60–69 | **2.86 (1.32 to 6.19)** | **0.008** | 1.52 (0.68 to 3.37) | 0.307 | N/A |  | N/A |  |
|  |  | ≥70 | **3.69 (1.70 to 8.00)** | **<.001** | 2.06 (0.92 to 4.59) | 0.078 | N/A |  | N/A |  |
| Female | Prevalence | 30-39 | 1.00 (ref) |  | 1.00 (ref) |  | 1.00 (ref) |  | 1.00 (ref) |  |
|  |  | 40–49 | **2.41 (1.99 to 2.92)** | **<.001** | **2.57 (2.09 to 3.17)** | **<.001** | **1.81 (1.14 to 2.87)** | **0.011** | 0.71 (0.43 to 1.17) | 0.176 |
|  |  | 50–59 | **5.42 (4.53 to 6.48)** | **<.001** | **5.49 (4.51 to 6.67)** | **<.001** | **4.99 (3.24 to 7.70)** | **<.001** | 0.91 (0.57 to 1.46) | 0.698 |
|  |  | 60–69 | **11.52 (9.70 to 13.69)** | **<.001** | **12.13 (10.07 to 14.60)** | **<.001** | **9.30 (6.07 to 14.23)** | **<.001** | 0.77 (0.48 to 1.22) | 0.262 |
|  |  | ≥70 | **17.64 (14.86 to 20.95)** | **<.001** | **17.91 (14.86 to 21.59)** | **<.001** | **16.05 (10.54 to 24.42)** | **<.001** | 0.90 (0.57 to 1.42) | 0.639 |
|  | Awareness | 30-39 | 1.00 (ref) |  | 1.00 (ref) |  | 1.00 (ref) |  | 1.00 (ref) |  |
|  |  | 40–49 | 1.18 (0.80 to 1.75) | 0.412 | 1.26 (0.83 to 1.93) | 0.282 | 0.89 (0.34 to 2.33) | 0.806 | 0.70 (0.24 to 2.01) | 0.510 |
|  |  | 50–59 | **1.78 (1.24 to 2.55)** | **0.002** | **1.84 (1.25 to 2.71)** | **0.002** | 1.56 (0.63 to 3.85) | 0.332 | 0.85 (0.32 to 2.26) | 0.742 |
|  |  | 60–69 | **3.08 (2.16 to 4.40)** | **<.001** | **3.06 (2.08 to 4.50)** | **<.001** | **3.07 (1.28 to 7.33)** | **0.012** | 1.00 (0.39 to 2.60) | 0.995 |
|  |  | ≥70 | **4.51 (3.17 to 6.41)** | **<.001** | **4.43 (3.02 to 6.50)** | **<.001** | **4.71 (1.99 to 11.17)** | **<.001** | 1.06 (0.41 to 2.74) | 0.899 |
|  | Treatment | 30-39 | 1.00 (ref) |  | 1.00 (ref) |  | 1.00 (ref) |  | 1.00 (ref) |  |
|  |  | 40–49 | **1.68 (1.10 to 2.58)** | **0.017** | **2.09 (1.31 to 3.35)** | **0.002** | 0.83 (0.30 to 2.26) | 0.711 | 0.40 (0.13 to 1.20) | 0.101 |
|  |  | 50–59 | **2.82 (1.91 to 4.17)** | **<.001** | **3.30 (2.14 to 5.09)** | **<.001** | 1.68 (0.68 to 4.13) | 0.262 | 0.51 (0.19 to 1.38) | 0.186 |
|  |  | 60–69 | **4.88 (3.32 to 7.20)** | **<.001** | **5.59 (3.63 to 8.63)** | **<.001** | **3.10 (1.29 to 7.41)** | **0.011** | 0.55 (0.21 to 1.47) | 0.235 |
|  |  | ≥70 | **6.50 (4.44 to 9.51)** | **<.001** | **7.39 (4.83 to 11.31)** | **<.001** | **4.34 (1.84 to 10.24)** | **<.001** | 0.59 (0.22 to 1.53) | 0.276 |
|  | Control among participants with diabetes | 30-39 | 1.00 (ref) |  | 1.00 (ref) |  | 1.00 (ref) |  | 1.00 (ref) |  |
|  |  | 40–49 | **0.56 (0.35 to 0.88)** | **0.012** | 0.61 (0.37 to 1.01) | 0.056 | 0.43 (0.15 to 1.24) | 0.118 | 0.71 (0.22 to 2.28) | 0.565 |
|  |  | 50–59 | 0.72 (0.48 to 1.09) | 0.120 | 0.75 (0.48 to 1.18) | 0.218 | 0.63 (0.26 to 1.54) | 0.313 | 0.84 (0.31 to 2.29) | 0.737 |
|  |  | 60–69 | **0.65 (0.44 to 0.96)** | **0.031** | 0.71 (0.46 to 1.11) | 0.137 | 0.47 (0.20 to 1.11) | 0.086 | 0.66 (0.25 to 1.74) | 0.404 |
|  |  | ≥70 | 0.88 (0.60 to 1.29) | 0.504 | 0.93 (0.60 to 1.42) | 0.724 | 0.72 (0.31 to 1.69) | 0.449 | 0.78 (0.30 to 2.02) | 0.604 |
|  | Control among participants being treated | 30-39 | 1.00 (ref) |  | 1.00 (ref) |  | 1.00 (ref) |  | 1.00 (ref) |  |
|  |  | 40–49 | **0.32 (0.14 to 0.74)** | **0.008** | 0.41 (0.16 to 1.08) | 0.072 | 0.30 (0.06 to 1.43) | 0.130 | 0.73 (0.12 to 4.55) | 0.736 |
|  |  | 50–59 | 0.72 (0.35 to 1.49) | 0.377 | 0.96 (0.41 to 2.26) | 0.922 | 0.50 (0.14 to 1.80) | 0.289 | 0.52 (0.11 to 2.45) | 0.410 |
|  |  | 60–69 | 0.65 (0.32 to 1.33) | 0.241 | 0.95 (0.41 to 2.20) | 0.913 | 0.36 (0.10 to 1.26) | 0.109 | 0.37 (0.08 to 1.70) | 0.203 |
|  |  | ≥70 | 0.92 (0.45 to 1.86) | 0.806 | 1.29 (0.56 to 2.94) | 0.551 | 0.55 (0.16 to 1.92) | 0.347 | 0.43 (0.10 to 1.91) | 0.266 |

Abbreviations: CI, confidence interval; KNHANES, Korea National Health and Nutrition Examination Survey; OR, odds ratio.

Numbers in bold indicate a significant difference (P < 0.05).

**Table S8.** Weighted odds ratios of prevalence, awareness, treatment, control among participants with diabetes, and control among participants being treated by region of residence among males and females based on data obtained from the KNHANES.

| Sex | Rate | Region of residence | Overall  (1998–2022) | | Before the pandemic  (1998–2019) | | During the pandemic  (2020–2022) | | Ratio of ORs during the pandemic compared to before the pandemic (reference) | |
| --- | --- | --- | --- | --- | --- | --- | --- | --- | --- | --- |
|  |  |  | Weighted ORs  (95% CI) | P-value | Weighted ORs  (95% CI) | P-value | Weighted ORs  (95% CI) | P-value | Weighted ratio of ORs  (95% CI) | P-value |
| Male | Prevalence | Urban | 1.00 (ref) |  | 1.00 (ref) |  | 1.00 (ref) |  | 1.00 (ref) |  |
|  |  | Rural | **1.23 (1.12 to 1.34)** | **<.001** | **1.26 (1.14 to 1.39)** | **<.001** | 1.17 (0.98 to 1.39) | 0.076 | 0.93 (0.76 to 1.14) | 0.492 |
|  | Awareness | Urban | 1.00 (ref) |  | 1.00 (ref) |  | 1.00 (ref) |  | 1.00 (ref) |  |
|  |  | Rural | 1.06 (0.90 to 1.25) | 0.489 | 1.07 (0.89 to 1.30) | 0.469 | 1.07 (0.75 to 1.51) | 0.714 | 1.00 (0.67 to 1.48) | 0.982 |
|  | Treatment | Urban | 1.00 (ref) |  | 1.00 (ref) |  | 1.00 (ref) |  | 1.00 (ref) |  |
|  |  | Rural | 1.07 (0.92 to 1.26) | 0.381 | 1.09 (0.91 to 1.30) | 0.363 | 1.09 (0.79 to 1.50) | 0.595 | 1.00 (0.70 to 1.45) | 0.984 |
|  | Control among participants with diabetes | Urban | 1.00 (ref) |  | 1.00 (ref) |  | 1.00 (ref) |  | 1.00 (ref) |  |
|  |  | Rural | 1.10 (0.93 to 1.30) | 0.248 | 1.07 (0.88 to 1.30) | 0.496 | 1.24 (0.91 to 1.69) | 0.182 | 1.16 (0.80 to 1.67) | 0.441 |
|  | Control among participants being treated | Urban | 1.00 (ref) |  | 1.00 (ref) |  | 1.00 (ref) |  | 1.00 (ref) |  |
|  |  | Rural | 1.10 (0.90 to 1.35) | 0.368 | 1.10 (0.86 to 1.40) | 0.467 | 1.16 (0.81 to 1.67) | 0.408 | 1.06 (0.69 to 1.64) | 0.783 |
| Female | Prevalence | Urban | 1.00 (ref) |  | 1.00 (ref) |  | 1.00 (ref) |  | 1.00 (ref) |  |
|  |  | Rural | **1.33 (1.22 to 1.46)** | **<.001** | **1.28 (1.16 to 1.42)** | **<.001** | **1.59 (1.31 to 1.93)** | **<.001** | 1.24 (1.00 to 1.54) | 0.055 |
|  | Awareness | Urban | 1.00 (ref) |  | 1.00 (ref) |  | 1.00 (ref) |  | 1.00 (ref) |  |
|  |  | Rural | 0.98 (0.84 to 1.15) | 0.798 | 0.97 (0.82 to 1.16) | 0.752 | 1.02 (0.72 to 1.44) | 0.927 | 1.05 (0.71 to 1.54) | 0.824 |
|  | Treatment | Urban | 1.00 (ref) |  | 1.00 (ref) |  | 1.00 (ref) |  | 1.00 (ref) |  |
|  |  | Rural | 1.01 (0.87 to 1.17) | 0.934 | 1.00 (0.85 to 1.18) | 0.992 | 1.05 (0.76 to 1.46) | 0.764 | 1.05 (0.73 to 1.52) | 0.783 |
|  | Control among participants with diabetes | Urban | 1.00 (ref) |  | 1.00 (ref) |  | 1.00 (ref) |  | 1.00 (ref) |  |
|  |  | Rural | 1.01 (0.85 to 1.20) | 0.895 | 0.99 (0.80 to 1.21) | 0.905 | 1.08 (0.79 to 1.49) | 0.630 | 1.09 (0.75 to 1.60) | 0.642 |
|  | Control among participants being treated | Urban | 1.00 (ref) |  | 1.00 (ref) |  | 1.00 (ref) |  | 1.00 (ref) |  |
|  |  | Rural | 1.06 (0.86 to 1.30) | 0.614 | 1.01 (0.79 to 1.29) | 0.964 | 1.17 (0.80 to 1.71) | 0.429 | 1.16 (0.73 to 1.83) | 0.524 |

Abbreviations: CI, confidence interval; KNHANES, Korea National Health and Nutrition Examination Survey; OR, odds ratio.

Numbers in bold indicate a significant difference (P < 0.05).

**Table S9.** Weighted odds ratios of prevalence, awareness, treatment, control among participants with diabetes, and control among participants being treated by BMI group among males and females based on data obtained from the KNHANES.

| Sex | Rate | BMI group ^a^ | Overall  (1998–2022) | | Before the pandemic  (1998–2019) | | During the pandemic  (2020–2022) | | Ratio of ORs during the pandemic compared to before the pandemic (reference) | |
| --- | --- | --- | --- | --- | --- | --- | --- | --- | --- | --- |
|  |  |  | Weighted ORs  (95% CI) | P-value | Weighted ORs  (95% CI) | P-value | Weighted ORs  (95% CI) | P-value | Weighted ratio of ORs  (95% CI) | P-value |
| Male | Prevalence | Underweight | 1.00 (ref) |  | 1.00 (ref) |  | 1.00 (ref) |  | 1.00 (ref) |  |
|  |  | Normal | **1.63 (1.17 to 2.26)** | **0.004** | **1.51 (1.09 to 2.09)** | **0.014** | 2.26 (0.75 to 6.77) | 0.146 | 1.50 (0.48 to 4.71) | 0.489 |
|  |  | Overweight | **1.85 (1.33 to 2.58)** | **<.001** | **1.73 (1.24 to 2.41)** | **0.001** | 2.41 (0.81 to 7.16) | 0.113 | 1.40 (0.45 to 4.36) | 0.564 |
|  |  | Obese | **2.62 (1.89 to 3.62)** | **<.001** | **2.39 (1.73 to 3.31)** | **<.001** | **3.51 (1.20 to 10.32)** | **0.023** | 1.47 (0.48 to 4.53) | 0.504 |
|  | Awareness | Underweight | 1.00 (ref) |  | 1.00 (ref) |  | 1.00 (ref) |  | 1.00 (ref) |  |
|  |  | Normal | 1.19 (0.59 to 2.39) | 0.631 | 1.33 (0.66 to 2.70) | 0.428 | 0.26 (0.04 to 1.54) | 0.138 | 0.20 (0.03 to 1.32) | 0.094 |
|  |  | Overweight | 0.98 (0.48 to 1.97) | 0.949 | 1.15 (0.56 to 2.35) | 0.700 | **0.17 (0.03 to 1.00)** | **0.049** | **0.15 (0.02 to 1.00)** | **0.050** |
|  |  | Obese | 0.65 (0.33 to 1.30) | 0.226 | 0.76 (0.38 to 1.53) | 0.440 | **0.11 (0.02 to 0.63)** | **0.013** | **0.15 (0.02 to 0.96)** | **0.045** |
|  | Treatment | Underweight | 1.00 (ref) |  | 1.00 (ref) |  | 1.00 (ref) |  | 1.00 (ref) |  |
|  |  | Normal | 1.47 (0.77 to 2.78) | 0.240 | 1.69 (0.90 to 3.20) | 0.105 | 0.36 (0.07 to 1.83) | 0.218 | 0.21 (0.04 to 1.22) | 0.082 |
|  |  | Overweight | 1.38 (0.73 to 2.62) | 0.323 | 1.67 (0.88 to 3.16) | 0.117 | 0.27 (0.05 to 1.36) | 0.112 | **0.16 (0.03 to 0.92)** | **0.040** |
|  |  | Obese | 1.00 (0.53 to 1.89) | 0.999 | 1.22 (0.65 to 2.29) | 0.541 | **0.18 (0.04 to 0.87)** | **0.033** | **0.14 (0.03 to 0.80)** | **0.027** |
|  | Control among participants with diabetes | Underweight | 1.00 (ref) |  | 1.00 (ref) |  | 1.00 (ref) |  | 1.00 (ref) |  |
|  |  | Normal | 0.66 (0.33 to 1.32) | 0.243 | 0.79 (0.42 to 1.46) | 0.448 | 0.32 (0.05 to 2.00) | 0.223 | 0.41 (0.06 to 2.83) | 0.365 |
|  |  | Overweight | 0.52 (0.26 to 1.04) | 0.065 | 0.60 (0.32 to 1.12) | 0.106 | 0.27 (0.04 to 1.70) | 0.164 | 0.45 (0.07 to 3.18) | 0.427 |
|  |  | Obese | 0.53 (0.27 to 1.07) | 0.076 | 0.67 (0.36 to 1.25) | 0.208 | 0.22 (0.04 to 1.33) | 0.098 | 0.32 (0.05 to 2.18) | 0.245 |
|  | Control among participants being treated | Underweight | 1.00 (ref) |  | 1.00 (ref) |  | 1.00 (ref) |  | 1.00 (ref) |  |
|  |  | Normal | 0.42 (0.16 to 1.10) | 0.076 | 0.50 (0.21 to 1.21) | 0.123 | 0.29 (0.04 to 2.13) | 0.222 | 0.57 (0.06 to 5.07) | 0.616 |
|  |  | Overweight | **0.37 (0.14 to 0.98)** | **0.046** | 0.42 (0.17 to 1.02) | 0.054 | 0.30 (0.04 to 2.20) | 0.233 | 0.70 (0.08 to 6.29) | 0.753 |
|  |  | Obese | **0.37 (0.14 to 0.96)** | **0.042** | 0.49 (0.21 to 1.17) | 0.109 | 0.19 (0.03 to 1.42) | 0.106 | 0.40 (0.04 to 3.51) | 0.405 |
| Female | Prevalence | Underweight | 1.00 (ref) |  | 1.00 (ref) |  | 1.00 (ref) |  | 1.00 (ref) |  |
|  |  | Normal | **2.00 (1.50 to 2.66)** | **<.001** | **2.16 (1.56 to 2.99)** | **<.001** | 1.72 (0.97 to 3.04) | 0.064 | 0.79 (0.41 to 1.53) | 0.494 |
|  |  | Overweight | **3.71 (2.78 to 4.95)** | **<.001** | **4.30 (3.11 to 5.95)** | **<.001** | **2.56 (1.43 to 4.59)** | **0.002** | 0.60 (0.31 to 1.16) | 0.128 |
|  |  | Obese | **7.11 (5.35 to 9.44)** | **<.001** | **8.16 (5.92 to 11.25)** | **<.001** | **5.05 (2.87 to 8.88)** | **<.001** | 0.62 (0.32 to 1.19) | 0.148 |
|  | Awareness | Underweight | 1.00 (ref) |  | 1.00 (ref) |  | 1.00 (ref) |  | 1.00 (ref) |  |
|  |  | Normal | 0.72 (0.34 to 1.50) | 0.375 | 0.62 (0.26 to 1.50) | 0.292 | 0.97 (0.25 to 3.70) | 0.961 | 1.55 (0.31 to 7.69) | 0.592 |
|  |  | Overweight | 0.51 (0.24 to 1.08) | 0.080 | **0.41 (0.17 to 1.00)** | **0.050** | 1.17 (0.30 to 4.51) | 0.823 | 2.86 (0.57 to 14.45) | 0.204 |
|  |  | Obese | **0.36 (0.17 to 0.75)** | **0.006** | **0.33 (0.13 to 0.79)** | **0.013** | 0.43 (0.11 to 1.59) | 0.205 | 1.31 (0.27 to 6.44) | 0.736 |
|  | Treatment | Underweight | 1.00 (ref) |  | 1.00 (ref) |  | 1.00 (ref) |  | 1.00 (ref) |  |
|  |  | Normal | 0.79 (0.43 to 1.43) | 0.431 | 0.73 (0.37 to 1.45) | 0.364 | 1.04 (0.32 to 3.39) | 0.947 | 1.43 (0.37 to 5.62) | 0.605 |
|  |  | Overweight | 0.65 (0.35 to 1.21) | 0.175 | 0.58 (0.29 to 1.16) | 0.120 | 1.36 (0.40 to 4.64) | 0.628 | 2.36 (0.57 to 9.70) | 0.235 |
|  |  | Obese | **0.51 (0.28 to 0.94)** | **0.031** | 0.52 (0.26 to 1.04) | 0.066 | 0.51 (0.15 to 1.71) | 0.273 | 0.97 (0.24 to 3.91) | 0.967 |
|  | Control among participants with diabetes | Underweight | 1.00 (ref) |  | 1.00 (ref) |  | 1.00 (ref) |  | 1.00 (ref) |  |
|  |  | Normal | 1.04 (0.53 to 2.05) | 0.917 | 0.80 (0.36 to 1.79) | 0.587 | 1.75 (0.50 to 6.06) | 0.380 | 2.18 (0.50 to 9.57) | 0.303 |
|  |  | Overweight | 0.88 (0.45 to 1.74) | 0.719 | 0.63 (0.28 to 1.41) | 0.263 | 1.96 (0.57 to 6.76) | 0.288 | 3.10 (0.71 to 13.54) | 0.133 |
|  |  | Obese | 0.72 (0.37 to 1.40) | 0.325 | 0.56 (0.26 to 1.24) | 0.154 | 1.17 (0.34 to 3.94) | 0.806 | 2.07 (0.48 to 8.84) | 0.326 |
|  | Control among participants being treated | Underweight | 1.00 (ref) |  | 1.00 (ref) |  | 1.00 (ref) |  | 1.00 (ref) |  |
|  |  | Normal | 0.85 (0.39 to 1.87) | 0.691 | 0.58 (0.23 to 1.45) | 0.245 | 1.86 (0.43 to 8.00) | 0.402 | 3.21 (0.57 to 17.97) | 0.184 |
|  |  | Overweight | 0.91 (0.41 to 2.00) | 0.815 | 0.61 (0.24 to 1.54) | 0.298 | 2.14 (0.51 to 9.04) | 0.300 | 3.49 (0.63 to 19.34) | 0.153 |
|  |  | Obese | 0.71 (0.33 to 1.53) | 0.378 | 0.51 (0.20 to 1.25) | 0.139 | 1.46 (0.36 to 6.03) | 0.597 | 2.90 (0.54 to 15.57) | 0.215 |

Abbreviations: CI, confidence interval; KNHANES, Korea National Health and Nutrition Examination Survey; OR, odds ratio.

Numbers in bold indicate a significant difference (P < 0.05).

^a^ BMI was divided into four groups according to Asian-Pacific guidelines: underweight (<18.5 kg/m^2^), normal (18.5-22.9 kg/m^2^), overweight (23.0–24.9 kg/m^2^), and obese (≥25 kg/m^2^).

**Table S10.** Weighted odds ratios of prevalence, awareness, treatment, control among participants with diabetes, and control among participants being treated by educational background among males and females based on data obtained from the KNHANES.

| Sex | Rate | Educational background | Overall  (1998–2022) | | Before the pandemic  (1998–2019) | | During the pandemic  (2020–2022) | | Ratio of ORs during the pandemic compared to before the pandemic (reference) | |
| --- | --- | --- | --- | --- | --- | --- | --- | --- | --- | --- |
|  |  |  | Weighted ORs  (95% CI) | P-value | Weighted ORs  (95% CI) | P-value | Weighted ORs  (95% CI) | P-value | Weighted ratio of ORs  (95% CI) | P-value |
| Male | Prevalence | Elementary school or lower | 1.00 (ref) |  | 1.00 (ref) |  | 1.00 (ref) |  | 1.00 (ref) |  |
|  |  | Middle school | 0.99 (0.88 to 1.11) | 0.838 | 0.96 (0.85 to 1.08) | 0.472 | 1.11 (0.83 to 1.48) | 0.490 | 1.16 (0.85 to 1.58) | 0.359 |
|  |  | High school | **0.63 (0.57 to 0.70)** | **<.001** | **0.59 (0.53 to 0.66)** | **<.001** | **0.71 (0.55 to 0.91)** | **0.007** | 1.19 (0.91 to 1.57) | 0.206 |
|  |  | College or higher | **0.38 (0.34 to 0.42)** | **<.001** | **0.35 (0.32 to 0.39)** | **<.001** | **0.39 (0.30 to 0.50)** | **<.001** | 1.10 (0.84 to 1.43) | 0.487 |
|  | Awareness | Elementary school or lower | 1.00 (ref) |  | 1.00 (ref) |  | 1.00 (ref) |  | 1.00 (ref) |  |
|  |  | Middle school | 0.94 (0.75 to 1.18) | 0.618 | 0.99 (0.77 to 1.26) | 0.905 | 0.76 (0.44 to 1.31) | 0.320 | 0.77 (0.42 to 1.40) | 0.394 |
|  |  | High school | **0.65 (0.53 to 0.79)** | **<.001** | **0.60 (0.49 to 0.75)** | **<.001** | 0.72 (0.44 to 1.16) | 0.176 | 1.19 (0.70 to 2.02) | 0.516 |
|  |  | College or higher | **0.53 (0.44 to 0.65)** | **<.001** | **0.48 (0.39 to 0.60)** | **<.001** | **0.60 (0.38 to 0.96)** | **0.031** | 1.25 (0.75 to 2.08) | 0.388 |
|  | Treatment | Elementary school or lower | 1.00 (ref) |  | 1.00 (ref) |  | 1.00 (ref) |  | 1.00 (ref) |  |
|  |  | Middle school | 0.99 (0.80 to 1.22) | 0.917 | 1.01 (0.80 to 1.28) | 0.923 | 0.83 (0.49 to 1.40) | 0.486 | 0.82 (0.46 to 1.45) | 0.499 |
|  |  | High school | **0.69 (0.57 to 0.83)** | **<.001** | **0.63 (0.52 to 0.77)** | **<.001** | 0.76 (0.49 to 1.20) | 0.239 | 1.21 (0.74 to 1.98) | 0.446 |
|  |  | College or higher | **0.57 (0.48 to 0.69)** | **<.001** | **0.51 (0.42 to 0.63)** | **<.001** | **0.60 (0.39 to 0.93)** | **0.021** | 1.17 (0.72 to 1.89) | 0.528 |
|  | Control among participants with diabetes | Elementary school or lower | 1.00 (ref) |  | 1.00 (ref) |  | 1.00 (ref) |  | 1.00 (ref) |  |
|  |  | Middle school | 0.97 (0.78 to 1.21) | 0.808 | 0.99 (0.77 to 1.26) | 0.915 | 0.91 (0.54 to 1.52) | 0.708 | 0.92 (0.52 to 1.62) | 0.771 |
|  |  | High school | 0.92 (0.76 to 1.12) | 0.408 | 0.89 (0.72 to 1.10) | 0.290 | 0.97 (0.61 to 1.52) | 0.878 | 1.08 (0.65 to 1.80) | 0.753 |
|  |  | College or higher | 0.89 (0.73 to 1.09) | 0.269 | 0.87 (0.70 to 1.10) | 0.246 | 0.89 (0.59 to 1.35) | 0.593 | 1.02 (0.64 to 1.64) | 0.929 |
|  | Control among participants being treated | Elementary school or lower | 1.00 (ref) |  | 1.00 (ref) |  | 1.00 (ref) |  | 1.00 (ref) |  |
|  |  | Middle school | 0.91 (0.69 to 1.20) | 0.499 | 0.94 (0.69 to 1.28) | 0.689 | 0.80 (0.43 to 1.48) | 0.472 | 0.85 (0.43 to 1.69) | 0.639 |
|  |  | High school | 0.88 (0.69 to 1.13) | 0.312 | 0.77 (0.58 to 1.02) | 0.064 | 1.05 (0.62 to 1.79) | 0.848 | 1.37 (0.75 to 2.50) | 0.303 |
|  |  | College or higher | 0.96 (0.74 to 1.23) | 0.724 | 0.92 (0.68 to 1.25) | 0.591 | 0.92 (0.56 to 1.51) | 0.751 | 1.00 (0.56 to 1.79) | 0.994 |
| Female | Prevalence | Elementary school or lower | 1.00 (ref) |  | 1.00 (ref) |  | 1.00 (ref) |  | 1.00 (ref) |  |
|  |  | Middle school | **0.59 (0.53 to 0.65)** | **<.001** | **0.57 (0.51 to 0.63)** | **<.001** | **0.64 (0.52 to 0.79)** | **<.001** | 1.13 (0.89 to 1.43) | 0.306 |
|  |  | High school | **0.29 (0.27 to 0.32)** | **<.001** | **0.27 (0.25 to 0.30)** | **<.001** | **0.34 (0.28 to 0.40)** | **<.001** | **1.24 (1.01 to 1.52)** | **0.039** |
|  |  | College or higher | **0.13 (0.12 to 0.15)** | **<.001** | **0.12 (0.10 to 0.14)** | **<.001** | **0.15 (0.12 to 0.18)** | **<.001** | 1.23 (0.94 to 1.60) | 0.130 |
|  | Awareness | Elementary school or lower | 1.00 (ref) |  | 1.00 (ref) |  | 1.00 (ref) |  | 1.00 (ref) |  |
|  |  | Middle school | **0.63 (0.51 to 0.77)** | **<.001** | **0.63 (0.51 to 0.79)** | **<.001** | **0.52 (0.32 to 0.84)** | **0.008** | 0.82 (0.48 to 1.40) | 0.472 |
|  |  | High school | **0.50 (0.42 to 0.59)** | **<.001** | **0.53 (0.44 to 0.64)** | **<.001** | **0.33 (0.22 to 0.49)** | **<.001** | **0.61 (0.39 to 0.96)** | **0.031** |
|  |  | College or higher | **0.44 (0.35 to 0.55)** | **<.001** | **0.41 (0.31 to 0.53)** | **<.001** | **0.36 (0.23 to 0.57)** | **<.001** | 0.89 (0.53 to 1.51) | 0.663 |
|  | Treatment | Elementary school or lower | 1.00 (ref) |  | 1.00 (ref) |  | 1.00 (ref) |  | 1.00 (ref) |  |
|  |  | Middle school | **0.69 (0.58 to 0.83)** | **<.001** | **0.69 (0.56 to 0.84)** | **<.001** | **0.58 (0.37 to 0.90)** | **0.016** | 0.84 (0.52 to 1.38) | 0.494 |
|  |  | High school | **0.54 (0.46 to 0.64)** | **<.001** | **0.56 (0.47 to 0.67)** | **<.001** | **0.36 (0.25 to 0.52)** | **<.001** | **0.63 (0.42 to 0.96)** | **0.032** |
|  |  | College or higher | **0.43 (0.34 to 0.53)** | **<.001** | **0.36 (0.28 to 0.46)** | **<.001** | **0.38 (0.25 to 0.59)** | **<.001** | 1.06 (0.64 to 1.76) | 0.808 |
|  | Control among participants with diabetes | Elementary school or lower | 1.00 (ref) |  | 1.00 (ref) |  | 1.00 (ref) |  | 1.00 (ref) |  |
|  |  | Middle school | **0.81 (0.66 to 1.00)** | **0.049** | 0.92 (0.73 to 1.16) | 0.487 | **0.51 (0.32 to 0.82)** | **0.005** | **0.55 (0.33 to 0.94)** | **0.028** |
|  |  | High school | **0.82 (0.67 to 0.99)** | **0.043** | **0.78 (0.62 to 0.97)** | **0.027** | 0.83 (0.57 to 1.22) | 0.338 | 1.07 (0.68 to 1.66) | 0.775 |
|  |  | College or higher | 1.06 (0.82 to 1.36) | 0.671 | 0.97 (0.72 to 1.32) | 0.866 | 1.02 (0.65 to 1.60) | 0.921 | 1.05 (0.61 to 1.80) | 0.859 |
|  | Control among participants being treated | Elementary school or lower | 1.00 (ref) |  | 1.00 (ref) |  | 1.00 (ref) |  | 1.00 (ref) |  |
|  |  | Middle school | 0.89 (0.70 to 1.15) | 0.371 | 1.00 (0.75 to 1.33) | 0.993 | 0.63 (0.38 to 1.04) | 0.072 | 0.63 (0.35 to 1.13) | 0.119 |
|  |  | High school | **0.75 (0.58 to 0.96)** | **0.022** | **0.72 (0.54 to 0.96)** | **0.024** | 0.73 (0.45 to 1.19) | 0.207 | 1.02 (0.58 to 1.80) | 0.939 |
|  |  | College or higher | 1.26 (0.88 to 1.80) | 0.202 | 1.00 (0.65 to 1.56) | 0.984 | 1.30 (0.74 to 2.28) | 0.370 | 1.29 (0.63 to 2.65) | 0.488 |

Abbreviations: CI, confidence interval; KNHANES, Korea National Health and Nutrition Examination Survey; OR, odds ratio.

Numbers in bold indicate a significant difference (P < 0.05).

**Table S11.** Weighted odds ratios of prevalence, awareness, treatment, control among participants with diabetes, and control among participants being treated by household income among males and females based on data obtained from the KNHANES.

| Sex | Rate | Household income | Overall  (1998–2022) | | Before the pandemic  (1998–2019) | | During the pandemic  (2020–2022) | | Ratio of ORs during the pandemic compared to before the pandemic (reference) | |
| --- | --- | --- | --- | --- | --- | --- | --- | --- | --- | --- |
|  |  |  | Weighted ORs  (95% CI) | P-value | Weighted ORs  (95% CI) | P-value | Weighted ORs  (95% CI) | P-value | Weighted ratio of ORs  (95% CI) | P-value |
| Male | Prevalence | Lowest quartile | 1.00 (ref) |  | 1.00 (ref) |  | 1.00 (ref) |  | 1.00 (ref) |  |
|  |  | Second quartile | **0.58 (0.53 to 0.64)** | **<.001** | **0.54 (0.49 to 0.61)** | **<.001** | **0.74 (0.59 to 0.93)** | **0.008** | **1.36 (1.06 to 1.75)** | **0.015** |
|  |  | Third quartile | **0.46 (0.41 to 0.50)** | **<.001** | **0.43 (0.38 to 0.48)** | **<.001** | **0.54 (0.43 to 0.66)** | **<.001** | 1.25 (0.99 to 1.58) | 0.066 |
|  |  | Highest quartile | **0.42 (0.38 to 0.47)** | **<.001** | **0.40 (0.36 to 0.45)** | **<.001** | **0.47 (0.38 to 0.58)** | **<.001** | 1.17 (0.91 to 1.49) | 0.216 |
|  | Awareness | Lowest quartile | 1.00 (ref) |  | 1.00 (ref) |  | 1.00 (ref) |  | 1.00 (ref) |  |
|  |  | Second quartile | **0.79 (0.65 to 0.96)** | **0.016** | **0.74 (0.60 to 0.92)** | **0.006** | 0.93 (0.60 to 1.46) | 0.757 | 1.26 (0.77 to 2.06) | 0.367 |
|  |  | Third quartile | **0.67 (0.55 to 0.81)** | **<.001** | **0.62 (0.50 to 0.77)** | **<.001** | 0.81 (0.54 to 1.23) | 0.329 | 1.32 (0.83 to 2.10) | 0.248 |
|  |  | Highest quartile | **0.72 (0.60 to 0.87)** | **<.001** | **0.68 (0.55 to 0.84)** | **<.001** | 0.80 (0.53 to 1.21) | 0.297 | 1.18 (0.74 to 1.87) | 0.493 |
|  | Treatment | Lowest quartile | 1.00 (ref) |  | 1.00 (ref) |  | 1.00 (ref) |  | 1.00 (ref) |  |
|  |  | Second quartile | **0.83 (0.70 to 0.99)** | **0.038** | **0.76 (0.63 to 0.93)** | **0.006** | 1.03 (0.68 to 1.57) | 0.892 | 1.35 (0.85 to 2.15) | 0.202 |
|  |  | Third quartile | **0.72 (0.60 to 0.87)** | **<.001** | **0.68 (0.56 to 0.83)** | **<.001** | 0.80 (0.53 to 1.22) | 0.303 | 1.18 (0.74 to 1.88) | 0.487 |
|  |  | Highest quartile | **0.80 (0.67 to 0.95)** | **0.011** | **0.73 (0.60 to 0.89)** | **0.002** | 0.94 (0.63 to 1.40) | 0.758 | 1.29 (0.82 to 2.01) | 0.269 |
|  | Control among participants with diabetes | Lowest quartile | 1.00 (ref) |  | 1.00 (ref) |  | 1.00 (ref) |  | 1.00 (ref) |  |
|  |  | Second quartile | 0.84 (0.69 to 1.01) | 0.063 | **0.76 (0.61 to 0.93)** | **0.010** | 1.09 (0.73 to 1.62) | 0.664 | 1.45 (0.92 to 2.27) | 0.109 |
|  |  | Third quartile | 0.86 (0.70 to 1.04) | 0.120 | 0.86 (0.69 to 1.08) | 0.203 | 0.83 (0.55 to 1.26) | 0.380 | 0.96 (0.60 to 1.54) | 0.875 |
|  |  | Highest quartile | **0.80 (0.66 to 0.98)** | **0.029** | **0.77 (0.62 to 0.97)** | **0.024** | 0.88 (0.58 to 1.33) | 0.527 | 1.13 (0.71 to 1.82) | 0.601 |
|  | Control among participants being treated | Lowest quartile | 1.00 (ref) |  | 1.00 (ref) |  | 1.00 (ref) |  | 1.00 (ref) |  |
|  |  | Second quartile | 0.84 (0.67 to 1.07) | 0.166 | **0.74 (0.56 to 0.98)** | **0.034** | 1.07 (0.68 to 1.68) | 0.767 | 1.45 (0.85 to 2.47) | 0.171 |
|  |  | Third quartile | 0.86 (0.67 to 1.10) | 0.224 | 0.86 (0.64 to 1.15) | 0.299 | 0.82 (0.50 to 1.35) | 0.434 | 0.96 (0.54 to 1.70) | 0.883 |
|  |  | Highest quartile | **0.72 (0.56 to 0.93)** | **0.013** | **0.68 (0.50 to 0.91)** | **0.011** | 0.77 (0.47 to 1.28) | 0.310 | 1.14 (0.63 to 2.04) | 0.667 |
| Female | Prevalence | Lowest quartile | 1.00 (ref) |  | 1.00 (ref) |  | 1.00 (ref) |  | 1.00 (ref) |  |
|  |  | Second quartile | **0.51 (0.47 to 0.56)** | **<.001** | **0.52 (0.47 to 0.57)** | **<.001** | **0.48 (0.39 to 0.58)** | **<.001** | 0.92 (0.74 to 1.14) | 0.465 |
|  |  | Third quartile | **0.33 (0.30 to 0.36)** | **<.001** | **0.33 (0.30 to 0.37)** | **<.001** | **0.32 (0.26 to 0.40)** | **<.001** | 0.98 (0.77 to 1.23) | 0.838 |
|  |  | Highest quartile | **0.25 (0.23 to 0.28)** | **<.001** | **0.25 (0.23 to 0.28)** | **<.001** | **0.23 (0.19 to 0.29)** | **<.001** | 0.91 (0.71 to 1.17) | 0.455 |
|  | Awareness | Lowest quartile | 1.00 (ref) |  | 1.00 (ref) |  | 1.00 (ref) |  | 1.00 (ref) |  |
|  |  | Second quartile | **0.64 (0.54 to 0.76)** | **<.001** | **0.69 (0.57 to 0.84)** | **<.001** | **0.49 (0.34 to 0.71)** | **<.001** | 0.71 (0.46 to 1.08) | 0.110 |
|  |  | Third quartile | **0.59 (0.49 to 0.71)** | **<.001** | **0.57 (0.46 to 0.70)** | **<.001** | **0.62 (0.41 to 0.93)** | **0.022** | 1.10 (0.69 to 1.74) | 0.692 |
|  |  | Highest quartile | **0.51 (0.41 to 0.62)** | **<.001** | **0.50 (0.39 to 0.63)** | **<.001** | **0.52 (0.33 to 0.82)** | **0.005** | 1.05 (0.63 to 1.74) | 0.861 |
|  | Treatment | Lowest quartile | 1.00 (ref) |  | 1.00 (ref) |  | 1.00 (ref) |  | 1.00 (ref) |  |
|  |  | Second quartile | **0.74 (0.62 to 0.87)** | **<.001** | **0.78 (0.65 to 0.94)** | **0.009** | **0.57 (0.41 to 0.81)** | **0.002** | 0.73 (0.49 to 1.08) | 0.118 |
|  |  | Third quartile | **0.66 (0.55 to 0.79)** | **<.001** | **0.63 (0.51 to 0.77)** | **<.001** | 0.69 (0.47 to 1.01) | 0.058 | 1.09 (0.71 to 1.69) | 0.688 |
|  |  | Highest quartile | **0.54 (0.45 to 0.66)** | **<.001** | **0.52 (0.42 to 0.64)** | **<.001** | **0.58 (0.38 to 0.88)** | **0.011** | 1.12 (0.70 to 1.79) | 0.644 |
|  | Control among participants with diabetes | Lowest quartile | 1.00 (ref) |  | 1.00 (ref) |  | 1.00 (ref) |  | 1.00 (ref) |  |
|  |  | Second quartile | **0.81 (0.67 to 0.97)** | **0.025** | 0.83 (0.67 to 1.02) | 0.077 | 0.76 (0.52 to 1.11) | 0.159 | 0.92 (0.59 to 1.42) | 0.701 |
|  |  | Third quartile | **0.81 (0.66 to 1.00)** | **0.045** | **0.73 (0.57 to 0.92)** | **0.009** | 1.03 (0.70 to 1.52) | 0.883 | 1.42 (0.90 to 2.25) | 0.134 |
|  |  | Highest quartile | 0.90 (0.72 to 1.13) | 0.364 | 0.90 (0.70 to 1.17) | 0.444 | 0.87 (0.55 to 1.38) | 0.555 | 0.96 (0.57 to 1.63) | 0.887 |
|  | Control among participants being treated | Lowest quartile | 1.00 (ref) |  | 1.00 (ref) |  | 1.00 (ref) |  | 1.00 (ref) |  |
|  |  | Second quartile | 0.82 (0.66 to 1.02) | 0.078 | 0.83 (0.65 to 1.07) | 0.153 | 0.80 (0.51 to 1.24) | 0.318 | 0.96 (0.58 to 1.60) | 0.876 |
|  |  | Third quartile | 0.82 (0.63 to 1.06) | 0.129 | **0.68 (0.50 to 0.92)** | **0.013** | 1.13 (0.71 to 1.79) | 0.614 | 1.67 (0.96 to 2.90) | 0.072 |
|  |  | Highest quartile | 0.80 (0.60 to 1.08) | 0.142 | 0.76 (0.54 to 1.07) | 0.111 | 0.85 (0.48 to 1.50) | 0.572 | 1.12 (0.57 to 2.17) | 0.746 |

Abbreviations: CI, confidence interval; KNHANES, Korea National Health and Nutrition Examination Survey; OR, odds ratio.

Numbers in bold indicate a significant difference (P < 0.05).

**Table S12.** Weighted odds ratios of prevalence, awareness, treatment, control among participants with diabetes, and control among participants being treated by smoking status among males and females based on data obtained from the KNHANES.

| Sex | Rate | Smoking status | Overall  (1998–2022) | | Before the pandemic  (1998–2019) | | During the pandemic  (2020–2022) | | Ratio of ORs during the pandemic compared to before the pandemic (reference) | |
| --- | --- | --- | --- | --- | --- | --- | --- | --- | --- | --- |
|  |  |  | Weighted ORs  (95% CI) | P-value | Weighted ORs  (95% CI) | P-value | Weighted ORs  (95% CI) | P-value | Weighted ratio of ORs  (95% CI) | P-value |
| Male | Prevalence | Non-smoker | 1.00 (ref) |  | 1.00 (ref) |  | 1.00 (ref) |  | 1.00 (ref) |  |
|  |  | Smoker | **1.27 (1.16 to 1.39)** | **<.001** | **1.22 (1.10 to 1.36)** | **<.001** | **1.48 (1.22 to 1.78)** | **<.001** | 1.21 (0.97 to 1.50) | 0.090 |
|  | Awareness | Non-smoker | 1.00 (ref) |  | 1.00 (ref) |  | 1.00 (ref) |  | 1.00 (ref) |  |
|  |  | Smoker | 1.09 (0.91 to 1.32) | 0.354 | 0.97 (0.79 to 1.21) | 0.801 | **1.55 (1.06 to 2.25)** | **0.023** | **1.59 (1.03 to 2.45)** | **0.035** |
|  | Treatment | Non-smoker | 1.00 (ref) |  | 1.00 (ref) |  | 1.00 (ref) |  | 1.00 (ref) |  |
|  |  | Smoker | 1.06 (0.89 to 1.26) | 0.536 | 0.94 (0.77 to 1.14) | 0.526 | **1.52 (1.07 to 2.16)** | **0.020** | **1.62 (1.08 to 2.43)** | **0.019** |
|  | Control among participants with diabetes | Non-smoker | 1.00 (ref) |  | 1.00 (ref) |  | 1.00 (ref) |  | 1.00 (ref) |  |
|  |  | Smoker | 0.98 (0.81 to 1.19) | 0.829 | 0.97 (0.78 to 1.20) | 0.746 | 1.02 (0.68 to 1.54) | 0.926 | 1.06 (0.66 to 1.68) | 0.815 |
|  | Control among participants being treated | Non-smoker | 1.00 (ref) |  | 1.00 (ref) |  | 1.00 (ref) |  | 1.00 (ref) |  |
|  |  | Smoker | 1.01 (0.78 to 1.30) | 0.967 | 0.95 (0.71 to 1.27) | 0.736 | 1.11 (0.66 to 1.87) | 0.685 | 1.17 (0.65 to 2.11) | 0.605 |
| Female | Prevalence | Non-smoker | 1.00 (ref) |  | 1.00 (ref) |  | 1.00 (ref) |  | 1.00 (ref) |  |
|  |  | Smoker | **0.82 (0.73 to 0.91)** | **<.001** | **0.84 (0.75 to 0.95)** | **0.007** | **0.73 (0.57 to 0.93)** | **0.012** | 0.86 (0.66 to 1.14) | 0.297 |
|  | Awareness | Non-smoker | 1.00 (ref) |  | 1.00 (ref) |  | 1.00 (ref) |  | 1.00 (ref) |  |
|  |  | Smoker | 0.95 (0.76 to 1.19) | 0.633 | 1.08 (0.83 to 1.40) | 0.557 | **0.61 (0.38 to 0.97)** | **0.038** | **0.56 (0.33 to 0.96)** | **0.036** |
|  | Treatment | Non-smoker | 1.00 (ref) |  | 1.00 (ref) |  | 1.00 (ref) |  | 1.00 (ref) |  |
|  |  | Smoker | 0.82 (0.66 to 1.01) | 0.058 | 0.92 (0.73 to 1.17) | 0.508 | **0.54 (0.34 to 0.84)** | **0.006** | **0.58 (0.35 to 0.96)** | **0.036** |
|  | Control among participants with diabetes | Non-smoker | 1.00 (ref) |  | 1.00 (ref) |  | 1.00 (ref) |  | 1.00 (ref) |  |
|  |  | Smoker | 1.10 (0.86 to 1.42) | 0.445 | 1.21 (0.91 to 1.61) | 0.189 | 0.84 (0.49 to 1.42) | 0.506 | 0.69 (0.38 to 1.26) | 0.228 |
|  | Control among participants being treated | Non-smoker | 1.00 (ref) |  | 1.00 (ref) |  | 1.00 (ref) |  | 1.00 (ref) |  |
|  |  | Smoker | 1.10 (0.81 to 1.51) | 0.533 | 1.33 (0.94 to 1.89) | 0.106 | 0.63 (0.33 to 1.23) | 0.177 | 0.48 (0.22 to 1.00) | 0.052 |

Abbreviations: CI, confidence interval; KNHANES, Korea National Health and Nutrition Examination Survey; OR, odds ratio.

Numbers in bold indicate a significant difference (P < 0.05).
